# Supplementary material for: Efficient Catalytic Synthesis of Condensed Isoxazole Derivatives via Intramolecular Oxidative Cycloaddition of Aldoximes
Source: Molecules. 2022 Jun 16;27(12):3860. doi: 10.3390/molecules27123860 (PMC9229713; doi:10.3390/molecules27123860)

Supporting Information

Efficient Catalytic Synthesis of Condensed Isoxazole Derivatives via Intramolecular Oxidative Cycloaddition of Aldoximes

Irina A. Mironova,[1] Valentine G. Nenajdenko,[2] Pavel S. Postnikov,[1] Akio Saito,[3] Mekhman S. Yusubov,*[1] and Akira Yoshimura*[4]

[1] Research School of Chemistry and Applied Biomedical Sciences, Tomsk Polytechnic University, 634050 Tomsk, Russia; irina190793@mail.ru (I.A.M.); pavelpostnikov@gmail.com (P.S.P.)
[2] Department of Chemistry, M. V. Lomonosov Moscow State University, 119991 Moscow, Russia; nenajdenko@gmail.com
[3] Division of Applied Chemistry, Institute of Engineering, Tokyo University of Agriculture and Technology, Tokyo 184-8588, Japan; akio-sai@cc.tuat.ac.jp
[4] Faculty of Pharmaceutical Sciences, Aomori University, 2-3-1 Kobata, Aomori 030-0943, Japan
* Correspondence: yusubov@mail.ru (M.S.Y.); ayoshimura@aomori-u.ac.jp (A.Y.)

Table of Contents pp

1. General experimental remarks S2
2. ESI-Mass study S2
3. ^{1}H NMR Spectroscopy study S3
4. General procedure for the synthesis of aldoximes **1** from 2-hydroxybenzaldehydes S4
5. X-Ray single crystal data of compound **3c** S16
6. References S17
7. NMR Spectra S18

1. *General experimental remarks*

All commercial reagents were ACS grade reagents and used without further purification from freshly opened containers. All solvents were distilled in prior to use. Melting points were determined in an open capillary tube with Buchi M-580 melting point apparatus. Infrared spectra were recorded as ATR on a P Agilent Cary 630 FT-IR spectrophotometer. NMR spectra were recorded on a Bruker BioSpin NMR spectrometer at 400 or 600 MHz (^{1}H NMR), 101 or 150 MHz (^{13}C NMR), 376 MHz (^{19}F NMR). Chemical shifts are reported in parts per million (ppm). High-resolution mass spectrometric measurements were performed using a Shimadzu LCMS-9030 Q-TOF mass spectrometer, coupled with LC-30 UHPLC system. X-ray crystal analysis was performed by Rigaku XtaLAB Synergy, Single source at home/near, HyPix using CuKα radiation (λ = 1.54184 Å) at 105 K. Please see the supporting information or the cif file for more detailed crystallography information. (*E*)-2-(Prop-2-yn-1-yloxy)benzaldehyde *O*-methyl oxime **5** was prepared according to the reported procedure.[1]

2. *ESI-Mass Study*

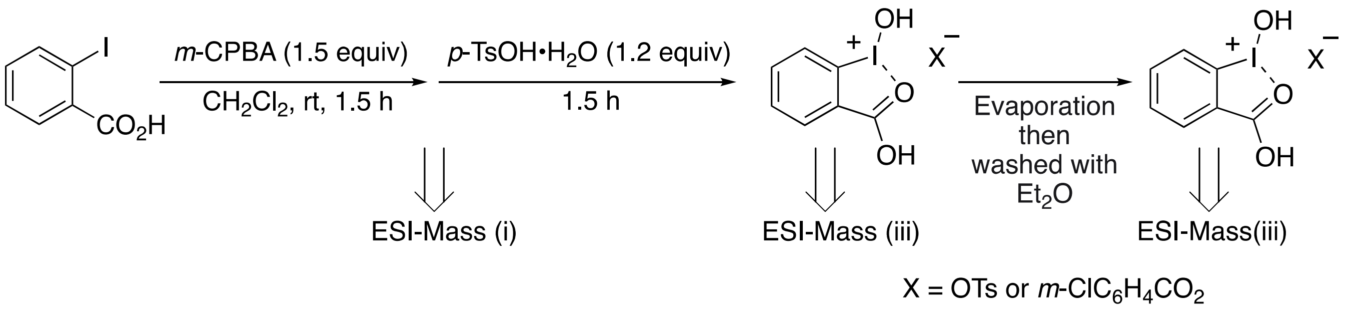

Scheme S1. ESI-Mass study of the generation of active species for intramolecular oxidative cycloaddition of aldoximes

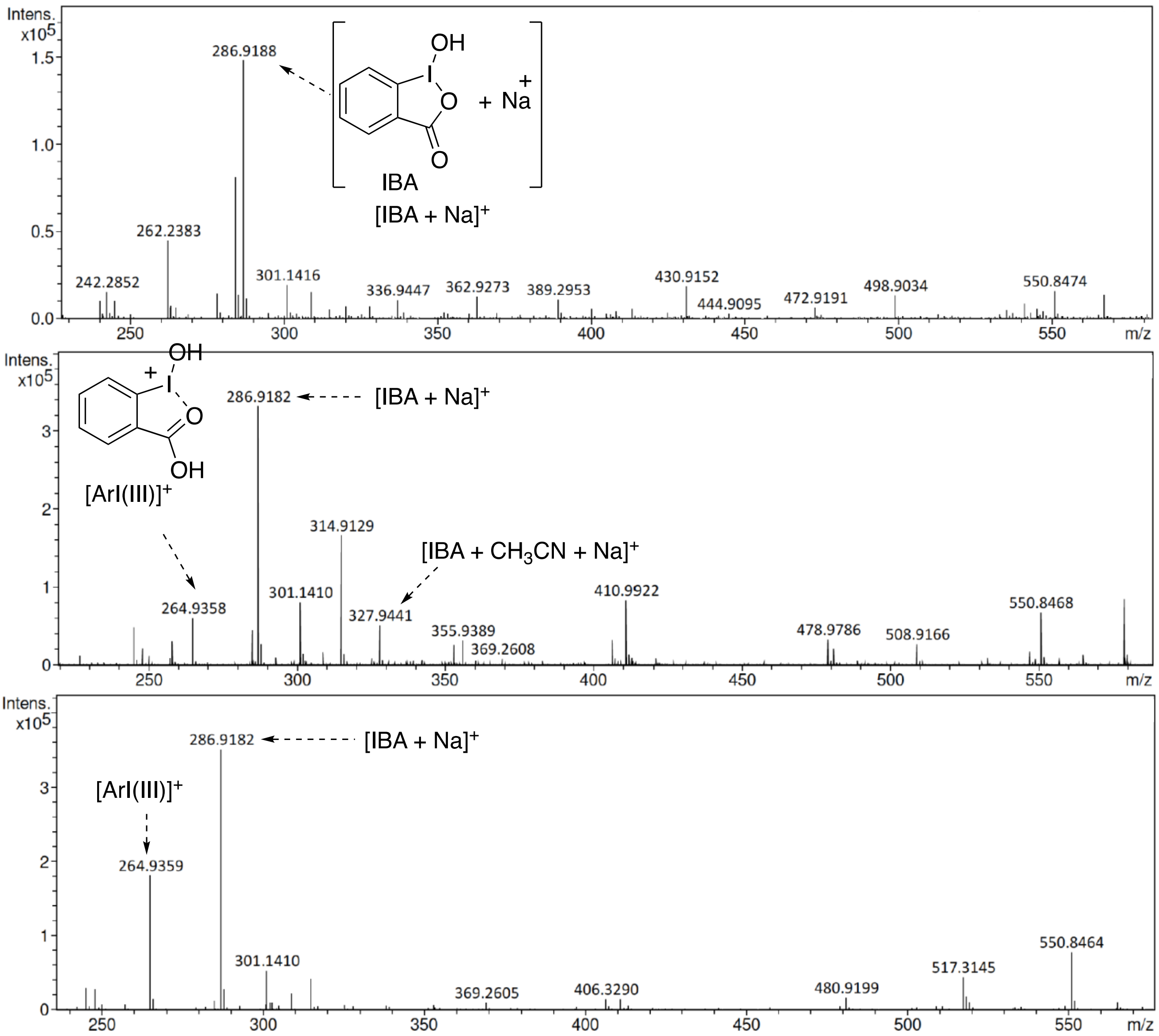

Figure S1. ESI-Mass study of the generation of active species for intramolecular oxidative cycloaddition of aldoximes

3. ¹H NMR Spectroscopy Study

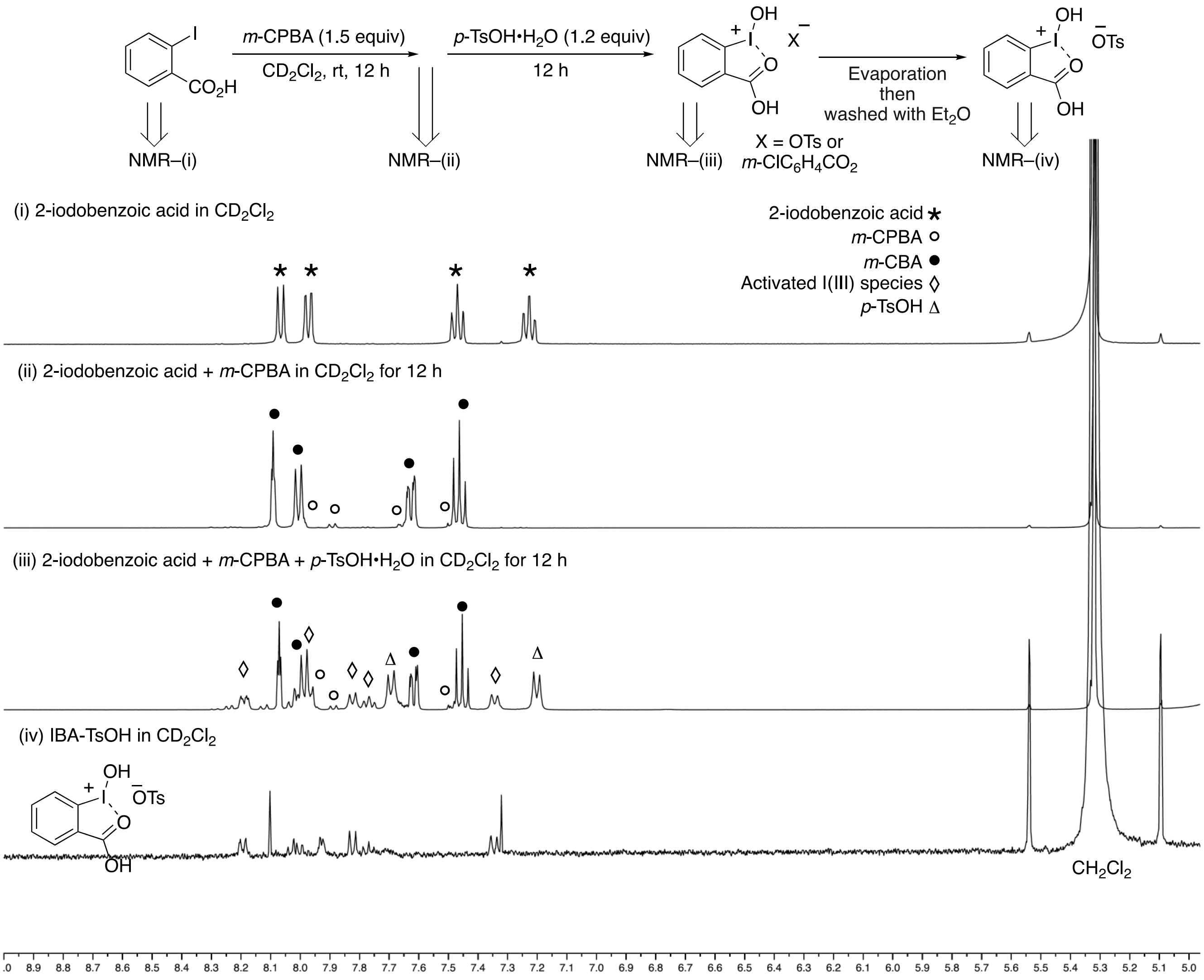

Figure S2. ^{1}H NMR spectroscopy study of the generation of active species for intramolecular oxidative cycloaddition of aldoximes

4. General procedure for the synthesis of aldoximes 1 from 2-hydroxybenzaldehydes

In a 50 mL round-bottom flask, dissolve 2-hydroxybenzaldehydes (2.0 mmol) in acetonitrile (20 mL). To this solution is added propargyl bromide (2.4 mmol, 286 mg) or allyl bromide (2.4 mmol, 290 mg), followed by anhydrous K_2CO_3 (4.0 mmol, 553 mg) and the reaction mixture was stirred under reflux for 8 h. The mixture was cooled to ambient temperature and water (20 ml) was added to the reaction mixture. The resulted mixture was extracted by dichloromethane, dried over $MgSO_4$ and evaporated under reduced pressure. Then, the crude 2-alkoxybenzaldehyde was dissolved in a mixed solution (MeOH-H_2O = 3.5 : 1 v/v)

followed by hydroxylamine hydrochloride (2.8 mmol, 195 mg) and AcONa (4.0 mmol, 328 mg) were added. The mixture was stirred at room temperature for 24 h. After completion of the reaction, water (10 mL) was added, and the mixture was extracted with dichloromethane. The organic layer was dried with $MgSO_4$ and concentrated under reduced pressure. The residue was purified by column chromatography (dichloromethane-hexane = 1 : 1) to afford the pure products **1**.

(*E*)-2-(Prop-2-yn-1-yloxy)benzaldehyde oxime (1a)[2]

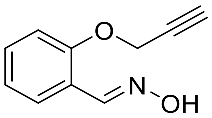

Reaction of 2-hydroxybenzaldehyde (2.0 mmol, 244 mg), propargyl bromide (2.4 mmol, 286 mg) and hydroxylamine hydrochloride (2.8 mmol, 195 mg) according to the general procedure afforded 284 mg (overall yield in 2 steps 81%) of product **1a**, isolated as colorless solid: mp 86.0 °C (lit.[2], mp 86 °C); IR (ATR) cm^{-1}: 3256, 3147, 3017, 2935, 2113, 1600, 1491, 1459, 1449, 1221, 1026, 747 657; ^{1}H NMR (400 MHz, CD_3OD): δ 8.37 (s, 1H), 7.71 (dd, J = 7.6, 1.6 Hz, 1H), 7.34-7.27 (m, 1H), 7.08 (d, J = 8.4 Hz, 1H), 6.98-6.90 (m, 1H), 4.77 (d, J = 2.4 Hz, 2H), 2.95 (t, J = 2.4 Hz, 1H).; ^{13}C NMR (101 MHz, CD_3OD): δ 156.8, 145.8, 131.7, 127.1, 123.2, 122.5, 114.2, 79.5, 77.1, 57.2.

(*E*)-5-Fluoro-2-(prop-2-yn-1-yloxy)benzaldehyde oxime (1b)

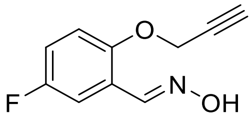

Reaction of 5-fluoro-2-hydroxybenzaldehyde (2.0 mmol, 280 mg), propargyl bromide (2.4 mmol, 286 mg) and hydroxylamine hydrochloride (2.8 mmol, 195 mg) according to the general afforded procedure 355 mg (overall yield in 2 steps 92%) of product **1b**, isolated as colorless solid: mp 106.7-107.8 °C; IR (ATR) cm^{-1}: 3303, 3147, 3022, 2933, 2124, 1590, 1491, 1432, 1260, 1182, 1017, 799, 660.; ^{1}H NMR (400 MHz, $CDCl_3$): δ 8.47 (s, 1H), 7.46 (dd, J = 9.0, 2.6 Hz, 1H), 7.11-6.95 (m, 2H), 4.73 (d, J = 1.6 Hz, 2H), 2.56-2.50 (m, 1H).; ^{13}C NMR (101 MHz, $CDCl_3$): δ 157.6 (d, $^{1}J_{CF}$ = 241.4 Hz), 151.9 (d, $^{4}J_{CF}$ = 2.0 Hz), 145.7 (d, $^{4}J_{CF}$ = 1.0 Hz), 123.0 (d, $^{3}J_{CF}$ = 8.1 Hz), 117.6 (d, $^{2}J_{CF}$ = 23.7 Hz), 114.7

(d, $^3J_{CF}$ = 8.2 Hz), 113.1 (d, $^2J_{CF}$ = 24.7 Hz), 78.0, 76.4, 57.3.: ^{19}F NMR (376 MHz, $CDCl_3$): δ -121.5; HRMS (ESI-positive mode): calcd for $C_{10}H_9FNO_2$ ($[M+H]^+$): 194.0612, found: 194.0614.

(*E*)-5-Chloro-2-(prop-2-yn-1-yloxy)benzaldehyde oxime (1c)[3]

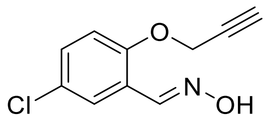

Reaction of 5-chloro-2-hydroxybenzaldehyde (2.0 mmol, 313 mg), propargyl bromide (2.4 mmol, 286 mg) and hydroxylamine hydrochloride (2.8 mmol, 195 mg) according to the general procedure afforded 306 mg (overall yield in 2 steps 73%) of product **1c**, isolated as colorless solid: mp 120.0-121.0 °C; IR (ATR) cm^{-1}: 3248, 3191, 2983, 2118, 1594, 1489, 1453, 1227, 1106, 1005, 740, 684; ^{1}H NMR (400 MHz, $CDCl_3$): δ 8.43 (s, 1H), 7.71 (d, *J* = 2.4 Hz, 1H), 7.31 (dd, *J* = 8.8, 2.4 Hz, 1H), 6.98 (d, *J* = 8.8 Hz, 1H), 4.74 (d, *J* = 2.4 Hz, 2H), 2.54 (t, *J* = 2.4 Hz, 1H); ^{13}C NMR (101 MHz, $CDCl_3$): δ 154.1, 145.5, 130.8, 127.2, 126.7, 122.9, 114.4, 77.8, 76.5, 56.8.; HRMS (ESI-positive mode): calcd for $C_{10}H_9{}^{35}ClNO_2$ ($[M+H]^+$): 210.0316, found: 210.0319.

(*E*)-5-Bromo-2-(prop-2-yn-1-yloxy)benzaldehyde oxime (1d)[4]

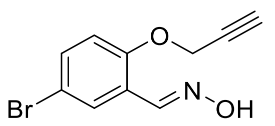

Reaction of 5-bromo-2-hydroxybenzaldehyde (2.0 mmol, 402 mg), propargyl bromide (2.4 mmol, 286 mg) and hydroxylamine hydrochloride (2.8 mmol, 195 mg) according to the general procedure 295 mg (overall yield in 2 steps 58%) of product **1d**, isolated as colorless solid: mp 127.0-128.0 °C; IR (ATR) cm^{-1}: 3243, 3186, 2979, 2920, 2117, 1590, 1486, 1453, 1225, 1005, 799, 671.; ^{1}H NMR (400 MHz, $CDCl_3$): δ 8.43 (s, 1H), 7.87 (d, *J* = 2.4 Hz, 1H), 7.45 (dd, *J* = 8.8, 2.4 Hz, 1H), 6.93 (d, *J* = 8.8 Hz, 1H), 4.74 (d, *J* = 2.3 Hz, 2H), 2.54 (t, *J* = 2.3 Hz, 1H).; ^{13}C NMR (101 MHz, $CDCl_3$): δ 154.7, 145.4, 133.7, 129.6, 123.3, 114.7, 114.5, 77.7, 76.6, 56.7.; HRMS (ESI-positive mode): calcd for $C_{10}H_9{}^{79}BrNO_2$ ($[M+H]^+$): 253.9811, found: 253.9812.

(*E*)-3,5-Dibromo-2-(prop-2-yn-1-yloxy)benzaldehyde oxime (1e)

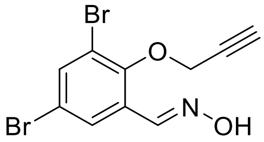

Reaction of 3,5-dibromo-2-hydroxybenzaldehyde (2.0 mmol, 560 mg), propargyl bromide (2.4 mmol, 286 mg) and hydroxylamine hydrochloride (2.8 mmol, 195 mg) according to the general procedure afforded 373 mg (overall yield in 2 steps 56%) of product **1e**, isolated as colorless solid: mp 122.0-123.0 °C; IR (ATR) cm^{-1}: 3490, 3289, 3236, 3067, 3001, 2926, 2119, 1544, 1438, 1221, 1088, 716, 665; ^{1}H NMR (400 MHz, $CDCl_3$): δ 8.46 (s, 1H), 7.92 (d, *J* = 2.4 Hz, 1H), 7.72 (d, *J* = 2.4 Hz, 1H), 4.74 (d, *J* = 2.4 Hz, 2H), 2.56 (t, *J* = 2.4 Hz, 1H).; ^{13}C NMR (101 MHz, $CDCl_3$): δ 152.4, 145.6, 137.0, 130.2, 128.6, 118.7, 118.6, 77.6, 61.7.; HRMS (ESI-positive mode): calcd for $C_{10}H_8{}^{79}Br_2NO_2$ ($[M+H]^+$): 331.8916, found: 331.8921.

(*E*)-5-Nitro-2-(prop-2-yn-1-yloxy)benzaldehyde oxime (1f)

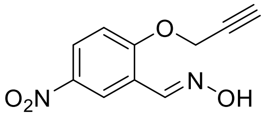

Reaction of 2-hydroxy-5-nitrobenzaldehyde (2.0 mmol, 334 mg), propargyl bromide (2.4 mmol, 286 mg) and hydroxylamine hydrochloride (2.8 mmol, 195 mg) according to the general procedure afforded 366 mg (overall yield in 2 steps 83%) of product **1f**, isolated as yellowish solid: mp 157.6-158.5 °C; IR (ATR) cm^{-1}: 3282, 3193, 2994, 2949, 2123, 1582, 1495, 1338, 1232, 1006, 839, 746, 682.; ^{1}H NMR (400 MHz, CD_3OD): δ 8.59 (d, *J* = 2.8 Hz, 1H), 8.34 (s, 1H), 8.23 (dd, *J* = 9.2, 2.8 Hz, 1H), 7.29 (d, *J* = 9.2 Hz, 1H), 4.97 (d, *J* = 2.3 Hz, 2H), 3.10 (t, *J* = 2.3 Hz, 1H).; ^{13}C NMR: (101 MHz, CD_3OD): δ 160.8, 143.5, 143.3, 126.7, 124.2, 122.3, 114.2, 78.3, 78.3, 57.9.; HRMS (ESI-positive mode): calcd for $C_{10}H_9N_2O_4$ ($[M+H]^+$): 221.0557, found: 221.0558.

(*E*)-5-Methyl-2-(prop-2-yn-1-yloxy)benzaldehyde oxime (1g)[5]

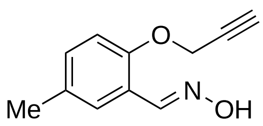

Reaction of 2-hydroxy-5-methylbenzaldehyde (2.0 mmol, 272 mg), propargyl bromide (2.4 mmol, 286 mg) and hydroxylamine hydrochloride (2.8 mmol, 195 mg) according to the general procedure afforded 352 mg (overall yield in 2 steps 93%) of product **1g**, isolated as colorless solid: mp 102.8-103.9 °C (lit. [5], mp 107-108 °C); IR (ATR) cm^{-1}: 3262, 3195, 2975, 2924, 2118, 1580, 1500, 1457, 1218, 1006, 926, 797, 697.; ^{1}H NMR (400 MHz, $CDCl_3$): δ 8.49 (s, 1H), 7.52 (d, *J* = 2.1 Hz, 1H), 7.17 (dd, *J* = 8.7, 2.1 Hz, 1H), 6.95 (d, *J* = 8.7 Hz, 1H), 4.73 (d, *J* = 2.4 Hz, 2H), 2.52 (t, *J* = 2.4 Hz, 1H), 2.30 (s, 3H).; ^{13}C NMR (101 MHz, $CDCl_3$): δ 153.7, 146.7, 131.8, 131.3, 127.5, 121.0, 113.1, 78.4, 76.0, 56.6, 20.6.

(*E*)-2-(Prop-2-yn-1-yloxy)-1-naphthaldehyde oxime (1h)[6]

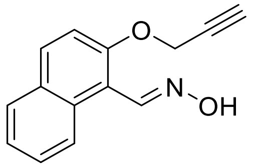

Reaction of 2-hydroxy-1-naphthaldehyde (2.0 mmol, 344 mg), propargyl bromide (2.4 mmol, 286 mg) and hydroxylamine hydrochloride (2.8 mmol, 195 mg) according to the general procedure afforded 342 mg (overall yield in 2 steps 76%) of product **1h**, isolated as colorless solid: mp 127.5-128.5 °C (lit.[6], mp 133-134 °C); IR (ATR) cm^{-1}: 3242, 3181, 2934, 2117, 1592, 1514, 1457, 1265, 1213, 1092, 1023, 749, 687.; ^{1}H NMR (400 MHz, CD_3OD): δ 8.87 (d, *J* = 8.6 Hz, 1H), 8.75 (s, 1H), 7.89 (d, *J* = 9.2 Hz, 1H), 7.81 (d, *J* = 8.4 Hz, 1H), 7.51-7.46 (m, 1H), 7.45 (d, *J* = 8.4 Hz, 1H), 7.41-7.35 (m, 1H), 4.93 (d, *J* = 2.4 Hz, 3H), 2.99 (t, *J* = 2.4 Hz, 1H).; ^{13}C NMR (101 MHz, CD_3OD): δ 156.2, 147.5, 132.8, 132.5, 131.2, 129.3, 128.5, 127.2, 125.4, 116.7, 115.7, 79.6, 77.3, 58.2.

(*E*)-2-Chloro-6-(prop-2-yn-1-yloxy)benzaldehyde oxime (1i)

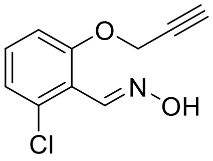

Reaction of 2-chloro-6-hydroxybenzaldehyde (2.0 mmol, 313 mg), propargyl bromide (2.4 mmol, 286 mg) and hydroxylamine hydrochloride (2.8 mmol, 195 mg) according to the general procedure afforded 268 mg (overall yield in 2 steps 64%) of product **1i**, isolated as colorless solid: mp 157.0-158.0 °C (decomp.); IR (ATR) cm^{-1}: 3290, 3103, 3010, 2921, 2854, 2115, 1569, 1449, 1275, 1208, 1025, 767, 666.; ^{1}H NMR (400 MHz, DMSO-d_6) δ 11.5 (s, 1H), 8.15 (s, 1H), 7.38 (t, *J* = 8.2 Hz, 1H), 7.18-7.11 (m, 2H), 4.89 (d, *J* = 2.4 Hz, 2H), 3.64 (t, *J* = 2.4 Hz, 1H). ^{13}C NMR (101 MHz, DMSO-d_6) δ 156.6, 142.7, 132.7, 130.5, 122.8, 120.3, 112.1, 78.9, 78.8, 56.5.; HRMS (ESI-positive mode): calcd for $C_{10}H_9{}^{35}ClNO_2$ ($[M+H]^+$): 210.0316, found: 210.0319.

(*E*)-3-Chloro-2-(prop-2-yn-1-yloxy)benzaldehyde oxime (1j)

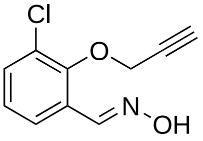

Reaction of 3-chloro-2-hydroxybenzaldehyde (2.0 mmol, 313 mg), propargyl bromide (2.4 mmol, 286 mg) and hydroxylamine hydrochloride (2.8 mmol, 195 mg) according to the general procedure afforded 277 mg (overall yield in 2 steps 66%) of product **1j**, isolated as colorless solid: mp 98.5-100.3 °C; IR (ATR) cm^{-1}: 3291, 3249, 3075, 3015, 2937, 2868, 2123, 1560, 1438, 1217, 1075, 777, 691.; ^{1}H NMR (400 MHz, $CDCl_3$): δ 8.56 (s, 1H), 7.71 (dd, *J* = 8.0, 1.6 Hz, 1H), 7.42 (dd, *J* = 8.0, 1.6 Hz, 1H), 7.11 (t, *J* = 8.0 Hz, 1H), 4.77 (d, *J* = 2.4 Hz, 2H), 2.55 (t, *J* = 2.4 Hz, 1H).; ^{13}C NMR (101 MHz, $CDCl_3$): δ 152.2, 146.6, 132.0, 128.6, 128.5, 125.9, 125.0, 77.9, 77.1, 61.4.; HRMS (ESI-positive mode): calcd for $C_{10}H_9{}^{35}ClNO_2$ ($[M+H]^+$): 210.0316, found: 210.0317.

(*E*)-2-(Allyloxy)benzaldehyde oxime (1k)[7]

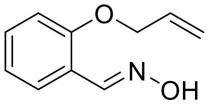

Reaction of 2-hydroxybenzaldehyde (2.0 mmol, 244 mg), allyl bromide (2.4 mmol, 290 mg) and hydroxylamine hydrochloride (2.8 mmol, 195 mg) according to the general procedure afforded 248 mg (overall yield in 2 steps 70%) of product **1k**, isolated as yellowish oil; IR (ATR) cm^{-1}: 3235, 3078, 3005, 2868, 1599, 1489, 1449, 1245, 994, 947, 751.; ^{1}H NMR (400 MHz, $CDCl_3$): δ 8.59 (s, 1H), 7.72 (d, *J* = 7.7, 1H), 7.37-7.29 (m, 1H), 6.97 (t, *J* = 7.7 Hz, 1H), 6.90 (d, *J* = 8.4 Hz, 1H), 6.14-5.98 (m, 1H), 5.42 (dd, *J* = 17.2, 1.2 Hz, 1H), 5.30 (d, *J* = 10.8 Hz, 1H), 4.59 (d, *J* = 5.2 Hz, 2H).; ^{13}C NMR (101 MHz, $CDCl_3$): δ 156.7, 146.6, 132.9, 131.3, 127.0, 121.1, 120.8, 117.8, 112.5, 69.2.

(*E*)-2-(Allyloxy)-5-fluorobenzaldehyde oxime (1l)

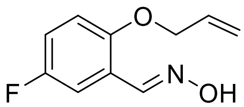

Reaction of 5-fluoro-2-hydroxybenzaldehyde (2.0 mmol, 280 mg), allyl bromide (2.4 mmol, 290 mg) and hydroxylamine hydrochloride (2.8 mmol, 195 mg) according to the general procedure afforded 355 mg (overall yield in 2 steps 91%) of product **1l**, isolated as colorless oil; IR (ATR) cm^{-1}: 3292, 3080, 3009, 2920, 2871, 1625, 1588, 1490, 1425, 1258, 1187, 1020, 937, 759.; ^{1}H NMR (400 MHz, $CDCl_3$): δ 8.64 (s, 1H), 8.51 (d, *J* = 1.6 Hz, 1H), 7.43 (dd, *J* = 9.0, 3.0 Hz, 1H), 7.06-6.98 (m, 1H), 6.84 (dd, *J* = 9.0, 4.2 Hz, 1H), 6.09-5.97 (m, 1H), 5.40 (dq, *J* = 17.2, 1.6 Hz, 1H), 5.34-5.26 (m, 1H), 4.55 (dt, *J* = 5.2, 1.6 Hz, 2H).; ^{13}C NMR (101 MHz, $CDCl_3$): δ 157.1 (d, $^1J_{CF}$ = 240.3 Hz), 153.0 (d, $^4J_{CF}$ = 2.0 Hz), 145.9 (d, $^4J_{CF}$ = 2.4 Hz), 132.8, 122.2 (d, $^3J_{CF}$ = 7.9 Hz), 118.1, 117.6 (d, $^2J_{CF}$ = 23.6 Hz), 114.0 (d, $^3J_{CF}$ = 8.2 Hz), 113.0 (d, $^2J_{CF}$ = 24.6 Hz), 70.1.; ^{19}F NMR (376 MHz, $CDCl_3$): δ -122.6. HRMS (ESI-positive mode): calcd for $C_{10}H_9{}^{35}ClNO_2$ ($[M+H]^+$): 196.0768, found: 196.0772.

(*E*)-2-(Allyloxy)-5-chlorobenzaldehyde oxime (1m)[8]

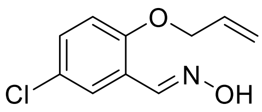

Reaction of 5-chloro-2-hydroxybenzaldehyde (2.0 mmol, 313 mg), allyl bromide (2.4 mmol, 290 mg) and hydroxylamine hydrochloride (2.8 mmol, 195 mg) according to the general procedure afforded 394 mg (overall yield in 2 steps 93%) of product **1m**, isolated as colorless solid: mp 80.4-80.9 °C (lit.[8], mp 100.7-101.7 °C); IR (ATR) cm^{-1}: 3400, 3167, 3080, 3003, 2861, 1594, 1477, 1451, 1255, 1129, 1002, 928, 758.; ^1H NMR (400 MHz, $CDCl_3$): δ 8.48 (s, 1H), 7.70 (d, *J* = 2.6 Hz, 1H), 7.27 (dd, *J* = 8.8, 2.6 Hz, 1H), 6.83 (d, *J* = 8.8 Hz, 1H), 6.13-5.94 (m, 1H), 5.40 (dd, *J* = 17.2, 1.5 Hz, 1H), 5.31 (dd, *J* = 10.6, 1.5 Hz, 1H), 4.57 (d, *J* = 5.2 Hz, 2H).; ^{13}C NMR (101 MHz, $CDCl_3$): δ 155.2, 145.6, 132.5, 130.9, 126.4, 126.3, 122.3, 118.3, 113.9, 69.67.

(*E*)-2-(Allyloxy)-5-bromobenzaldehyde oxime (1n)[8]

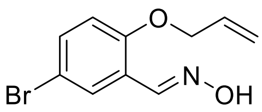

Reaction of 5-bromo-2-hydroxybenzaldehyde (2.0 mmol, 402 mg), allyl bromide (2.4 mmol, 290 mg) and hydroxylamine hydrochloride (2.8 mmol, 195 mg) according to the general procedure afforded 405 mg (overall yield in 2 steps 79%) of product **1n**, isolated as yellow solid: mp 84.3-85.3 °C (lit.[8], mp 87.0-88.5 °C); IR (ATR) cm^{-1}: 3242, 3138, 3080, 3019, 2915, 1589, 1484, 1454, 1249, 1018, 948, 796.; ^1H NMR (400 MHz, $CDCl_3$): δ 8.47 (s, 1H), 7.84 (d, *J* = 2.4 Hz, 1H), 7.41 (dd, *J* = 8.8, 2.4 Hz, 1H), 6.78 (d, *J* = 8.8 Hz, 1H), 6.08-5.96 (m, 1H), 5.40 (dq, *J* = 17.2, 1.6 Hz, 1H), 5.34-5.28 (m, 1H), 4.56 (dt, *J* = 5.2, 1.6 Hz, 2H).; ^{13}C NMR (101 MHz, $CDCl_3$): δ 155.7, 145.5, 133.8, 132.5, 129.4, 122.8, 118.3, 114.3, 113.6, 69.6.

(*E*)-2-(Allyloxy)-5-nitrobenzaldehyde oxime (1o)[9]

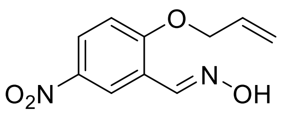

Reaction of 2-hydroxy-5-nitrobenzaldehyde (2.0 mmol, 334 mg), allyl bromide (2.4 mmol, 290 mg) and hydroxylamine hydrochloride (2.8 mmol, 195 mg) according to the general procedure afforded 258 mg (overall yield in 2 steps 58%) of product **1o**, isolated as colorless solid: mp 89.0-90.0 °C; IR (ATR) cm^{-1}: 3424, 3246, 3090, 3019, 2902, 2860, 1611, 1578, 1510, 1421, 1331, 1265, 1076, 926, 747.; ^1H NMR (400 MHz, $CDCl_3$): δ 8.65 (d, *J* = 2.8 Hz, 1H), 8.50 (s, 1H), 8.23 (dd, *J* = 9.2, 2.8 Hz, 1H), 6.97 (d, *J* = 9.2 Hz, 1H), 6.14-5.94 (m, 1H), 5.44 (d, *J* = 16.8 Hz, 1H), 5.38 (d, *J* = 10.4 Hz, 1H), 4.70 (d, *J* = 5.2 Hz, 2H).; ^{13}C NMR (101 MHz, $CDCl_3$): δ 160.9, 144.8, 141.7, 131.6, 126.7, 122.7, 121.8, 119.2, 112.2, 70.0.

(*E*)-2-(Allyloxy)-5-methylbenzaldehyde oxime (1p)[10]

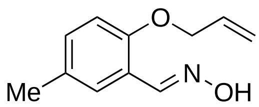

Reaction of 2-hydroxy-5-methylbenzaldehyde (2.0 mmol, 272 mg), allyl bromide (2.4 mmol, 290 mg) and hydroxylamine hydrochloride (2.8 mmol, 195 mg) according to the general procedure afforded 325 mg (overall yield in 2 steps 58%) of product **1p**, isolated as colorless solid: mp 67.2-68.2 °C; IR (ATR) cm^{-1}: 3216, 3081, 3019, 2923, 2872, 1610, 1497, 1426, 1250, 1025, 948, 799, 727.; ^1H NMR (400 MHz, $CDCl_3$): δ 8.54 (s, 1H), 7.53 (s, 1H), 7.14 (d, *J* = 8.2 Hz, 1H), 6.80 (d, *J* = 8.2 Hz, 1H), 6.13-5.96 (m, 1H), 5.41 (dt, *J* = 17.2, 1.8 Hz, 1H), 5.29 (dt, *J* = 10.8, 1.7 Hz, 1H), 4.56 (d, *J* = 4.8 Hz, 2H), 2.29 (s, 3H).; ^{13}C NMR (101 MHz, $CDCl_3$): δ 154.8, 146.8, 133.1, 132.0, 130.4, 127.2, 120.5, 117.8, 112.7, 69.5, 20.6.

(*E*)-2-(Allyloxy)-1-naphthaldehyde oxime (1q)[6]

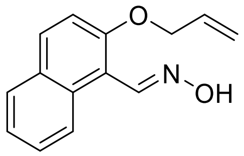

Reaction of 2-hydroxy-1-naphthaldehyde (2.0 mmol, 344 mg), allyl bromide (2.4 mmol, 290 mg) and

hydroxylamine hydrochloride (2.8 mmol, 195 mg) according to the general procedure afforded 327 mg (overall yield in 2 steps 72%) of product **1q**, isolated as colorless solid: mp 110.3-111.0 °C (lit.[6], mp 133.0-134.0 °C); IR (ATR) cm^{-1}: 3227, 3077, 2998, 2923, 2869, 1591, 1508, 1427, 1243, 1056, 1002, 946, 751.; ^1H NMR (400 MHz, $CDCl_3$): δ 8.95 (s, 1H), 8.78 (d, *J* = 8.4 Hz, 1H), 7.89 (d, *J* = 8.8 Hz, 1H), 7.82 (d, *J* = 8.0 Hz, 1H), 7.64-7.55 (m, 1H), 7.47-7.39 (m, 1H), 7.29 (d, *J* = 8.0 Hz, 1H), 6.21-6.07 (m, 1H), 5.48 (d, *J* = 17.6, 1H), 5.36 (d, *J* = 10.8, 1H), 4.81 (d, *J* = 4.8 Hz, 2H).; ^{13}C NMR (101 MHz, $CDCl_3$): δ 156.3, 147.8, 133.0, 132.4, 131.8, 129.3, 128.5, 128.1, 125.4, 124.4, 118.2, 114.2, 113.7, 70.5.

(*E*)-2-(Allyloxy)-6-chlorobenzaldehyde (1r)

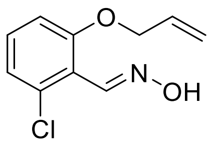

Reaction of 2-chloro-6-hydroxybenzaldehyde (2.0 mmol, 313 mg), allyl bromide (2.4 mmol, 290 mg) and hydroxylamine hydrochloride (2.8 mmol, 195 mg) according to the general procedure afforded 364 mg (overall yield in 2 steps 86%) of product **1r**, isolated as colorless solid: mp 84.0-85.0 °C; IR (ATR) cm^{-1}: 3178, 2951, 2896, 1587, 1568, 1459, 1419, 1269, 1207, 1021, 970, 932, 770.; ^1H NMR (400 MHz, $CDCl_3$): δ 8.57 (s, 1H), 7.35-7.23 (m, 1H), 7.07 (d, *J* = 8.0 Hz, 1H), 6.88 (d, *J* = 8.4 Hz, 1H), 6.15-5.95 (m, 1H), 5.42 (d, *J* = 17.2 Hz, 1H), 5.33 (d, *J* = 10.4 Hz, 1H), 4.81-4.67 (m, 2H).; ^{13}C NMR (101 MHz, $CDCl_3$): δ 158.5, 152.9, 145.1, 135.6, 132.2, 131.8, 122.9, 118.8, 111.5, 70.3; HRMS (ESI-positive mode): calcd for $C_{10}H_{10}{}^{35}ClNO_2$ ($[M+H]^+$): 212.0472, found: 212.0476.

(*E*)-2-(Allyloxy)-3-chlorobenzaldehyde oxime (1s)

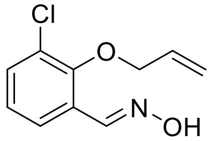

Reaction of 3-chloro-2-hydroxybenzaldehyde (2.0 mmol, 313 mg), allyl bromide (2.4 mmol, 290 mg) and hydroxylamine hydrochloride (2.8 mmol, 195 mg) according to the general procedure afforded 402 mg (overall

yield in 2 steps 86%) of product **1s**, isolated as colorless solid: mp 75.6-76.6 °C; IR (ATR) cm^{-1}: 3245, 3152, 3069, 3017, 2912, 2868, 1590, 1477, 1436, 1262, 1224, 1015, 964, 740.; ^1H NMR (400 MHz, $CDCl_3$): δ 8.43 (s, 1H), 7.73-7.67 (m, 1H), 7.43 (dd, *J* = 8.0, 1.6 Hz, 1H), 7.08 (t, *J* = 8.0 Hz, 1H), 6.19-6.01 (m, 1H), 5.44 (dq, *J* = 17.2, 1.2 Hz, 1H), 5.31 (dq, *J* = 10.4, 1.2 Hz, 1H), 4.51 (dt, *J* = 6.0, 1.2 Hz, 2H).; ^{13}C NMR (101 MHz, $CDCl_3$): δ 153.5, 146.2, 132.7, 132.1, 128.7, 127.6, 125.3, 125.1, 119.2, 75.6.; HRMS (ESI-positive mode): calcd for $C_{10}H_{10}{}^{35}ClNO_2$ ($[M+H]^+$): 212.0472, found: 212.0476.

(*E*)-2-((3-Phenylprop-2-yn-1-yl)oxy)benzaldehyde oxime (1t)

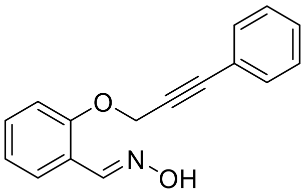

Reaction of 2-hydroxybenzaldehyde (2.0 mmol, 244 mg), (3-chloroprop-1-yn-1-yl)benzene (2.4 mmol, 362 mg) and hydroxylamine hydrochloride (2.8 mmol, 195 mg) according to the general procedure afforded 402 mg (overall yield in 2 steps 80%) of product **1t**, isolated as pale solid: mp 77.6-78.6 °C; IR (ATR) cm^{-1}: 3239, 3052, 2968, 2879, 2238, 1598, 1489, 1454, 1281, 1222, 1128, 1007, 970, 749, 689.; ^1H NMR (400 MHz, $CDCl_3$) δ 8.94 (s, 1H), 8.58 (s, 1H), 7.73 (dd, *J* = 7.8, 1.8 Hz, 1H), 7.47-7.42 (m, 2H), 7.42-7.36 (m, 1H), 7.36-7.27 (m, 3H), 7.14 (dd, *J* = 8.4, 1.2 Hz, 1H), 7.03 (m, 1H), 4.98 (s, 2H).; ^{13}C NMR (101 MHz, $CDCl_3$): δ 156.0, 146.7, 131.9, 131.2, 128.9, 128.4, 127.2, 122.2, 121.8, 121.4, 113.2, 87.7, 83.6, 57.3.; HRMS (ESI-positive mode): calcd for $C_{16}H_{13}NO_2$ ($[M+H]^+$): 252.1018, found: 252.1026.

(*E*)-2-(Cinnamyloxy)benzaldehyde oxime (1u)[11]

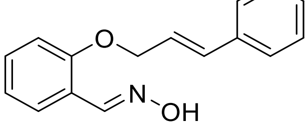

Reaction of 2-hydroxybenzaldehyde (2.0 mmol, 244 mg), (*E*)-(3-bromoprop-1-en-1-yl)benzene (2.4 mmol, 473 mg)

and hydroxylamine hydrochloride (2.8 mmol, 195 mg) according to the general procedure afforded 441 mg (overall yield in 2 steps 87%) of product **1u**, isolated as colorless solid: mp 129.7-130.7 °C (lit.[11] mp 104.0-106.0 °C); IR (ATR) cm^{-1}: 3285, 3076, 3013, 2913, 2861, 1597, 1490, 1446, 1295, 1236, 1107, 1001, 960, 751; ^{1}H NMR (400 MHz, DMSO-d$_6$) δ 11.26 (s, 1H), 8.38 (s, 1H), 7.68 (dd, *J* = 7.6, 1.6 Hz, 1H), 7.50 (d, *J* = 7.2 Hz, 2H), 7.40-7.32 (m, 3H), 7.28 (t, *J* = 7.4 Hz, 1H), 7.14 (d, *J* = 8.4 Hz, 1H), 6.97 (t, *J* = 7.6 Hz, 1H), 6.77 (d, *J* = 16.0 Hz, 1H), 6.54 (dt, *J* = 16.0, 5.8 Hz, 1H), 4.78 (dd, *J* = 5.8, 1.6 Hz, 2H).^{13}C NMR (101 MHz, DMSO-d$_6$) δ 155.9, 143.5, 136.1, 132.6, 130.7, 128.7, 128.0, 126.6, 125.5, 124.8, 121.3, 120.9, 113.2, 68.7.

(*E*)-2-(Cyclohex-2-en-1-yloxy)benzaldehyde oxime (1v)

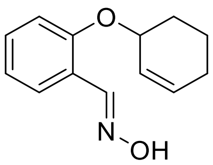

Reaction of 2-hydroxybenzaldehyde (2.0 mmol, 244 mg), 3-bromocyclohex-1-ene (2.4 mmol, 233 mg) and hydroxylamine hydrochloride (2.8 mmol, 195 mg) according to the general procedure afforded 195 mg (overall yield in 2 steps 45%) of product **1v**, isolated as colorless solid: mp 79.7-81.3 °C; IR (ATR) cm^{-1}: 3247, 3148, 3028, 2945, 2921, 2865, 1596, 1577, 1488, 1454, 1439, 1236, 1057, 947, 735; ^{1}H NMR (400 MHz, CDCl$_3$): δ 8.57 (s, 1H), 7.75 (dd, *J* = 7.6, 1.6 Hz, 1H), 7.36-7.28 (m, 1H), 6.99-6.92 (m, 2H), 6.02-5.93 (m, 1H), 5.91-5.82 (m, 1H), 4.88-4.77 (m, 1H), 2.21-2.09 (m, 1H), 2.09-1.80 (m, 4H), 1.71-1.60 (m, 1H).; ^{13}C NMR (101 MHz, CDCl$_3$) δ 156.3, 146.9, 132.5, 131.2, 126.7, 126.0, 121.9, 121.0, 114.1, 72.0, 28.6, 25.2, 19.1.; HRMS (ESI-positive mode): calcd for $C_{13}H_{15}NO_2$ ([M+H])$^+$: 218.1175, found: 218.1179.

5. X-Ray single crystal data of compound 3c

8-Chloro-4*H*-chromeno[4,3-*c*]isoxazole (3c)[2]

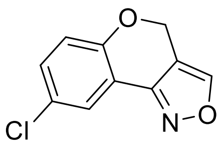

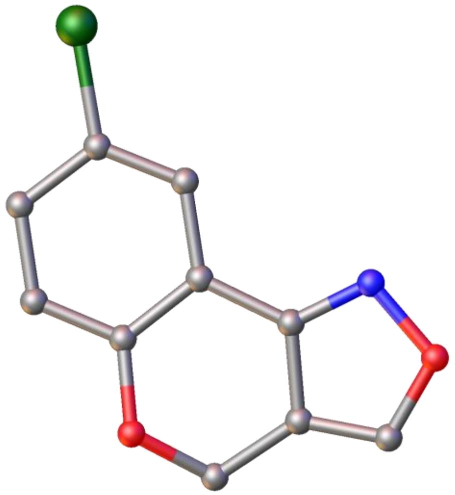

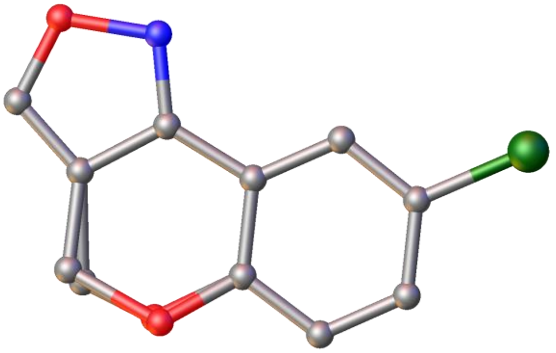

Single crystals of product **3c** suitable for X-ray crystallographic analysis were obtained by slow evaporation of acetonitrile solution. For details on crystal structure of compound **3c** see the CIF file in Supporting Information. Selected crystallographic data for **3c**: size 0.15 mm x 0.13 mm x 0.12 mm, orthorhombic, space group Pca21 (no. 29), a = 13.4924(6) Å, b = 3.89250(18) Å, c = 32.3452(14) Å, V = 1698.75(13) Å3, Z = 4, T = 105(9) K, μ(Cu Kα) = 3.732 mm^{-1}, Dcalc = 1.624 g/cm^3, 5818 reflections measured (5.464°≤ 2Θ ≤ 139.996°), 2510 unique (Rint = 0.0587, Rsigma = 0.0620) which were used in all calculations. The final R1 was 0.0644 (I > 2σ(I)) and wR2 was 0.1678 (all data). Using Olex2,[12] the structure was solved with the ShelXT[13] structure solution program using Intrinsic Phasing and refined with the ShelXL[14] refinement package using Least Squares minimization. Supplementary crystallographic data for this paper have been deposited at Cambridge Crystallographic Data Centre (CCDC number 21216235) and can be obtained free of charge via www.ccdc.cam.ac.uk/data_request/cif .

6. References:

1. S. E. Booth, P. R. Jenkins, C. J. Swain and J. B. Sweeney, *J. Chem. Soc., Perkin Trans. 1*, 1994, 3499-3508.
2. R. Fusco, L. Garanti and G. Zecchi, *Chim. Ind. (Milan)*, 1975, **57**, 16.
3. M. J. Raihan, V. Kavala, C.-W. Kuo, B. R. Raju and C.-F. Yao, *Green Chem.*, 2010, **12**, 1090-1096.
4. S. Bhosale, S. Kurhade, U. V. Prasad, V. P. Palle and D. Bhuniya, *Tetrahedron Lett.*, 2009, **50**, 3948-3951.
5. B. Roy and R. N. De, *Monatsh. Chem.*, 2010, **141**, 763-771.
6. T. Liaskopoulos, S. Skoulika, P. G. Tsoungas and G. Varvounis, *Synthesis*, 2008, 711-718.
7. C. Lambruschini, A. Basso, L. Moni, A. Pinna, R. Riva and L. Banfi, *Eur. J. Org. Chem.*, 2018, **2018**, 5445-5455.
8. J. I. Lee, H. S. Lee and B. H. Kim, *Synth. Commun.*, 1996, **26**, 3201-3215.

9. T. Shimizu, Y. Hayashi and K. Teramura, *Bull. Chem. Soc. Jpn.*, 1985, **58**, 397-398.
10. B. Roy, R. N. De and S. Hazra, *Lett. Org. Chem.*, 2011, **8**, 391-400.
11. K. Bala and H. C. Hailes, *Synthesis*, 2005, DOI: 10.1055/s-2005-918471, 3423-3427.
12. O. V. Dolomanov, L. J. Bourhis, R. J. Gildea, J. A. K. Howard and H. Puschmann, *J. Appl. Crystallogr.*, 2009, **42**, 339-341.
13. G. M. Sheldrick, *Acta Crystallogr., Sect. A: Found. Adv.*, 2015, **71**, 3-8.
14. G. M. Sheldrick, *Acta Crystallogr., Sect. C: Struct. Chem.*, 2015, **71**, 3-8.

^{1}H NMR (400 MHz, CD_3OD);

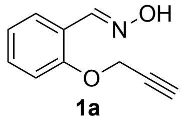

1a

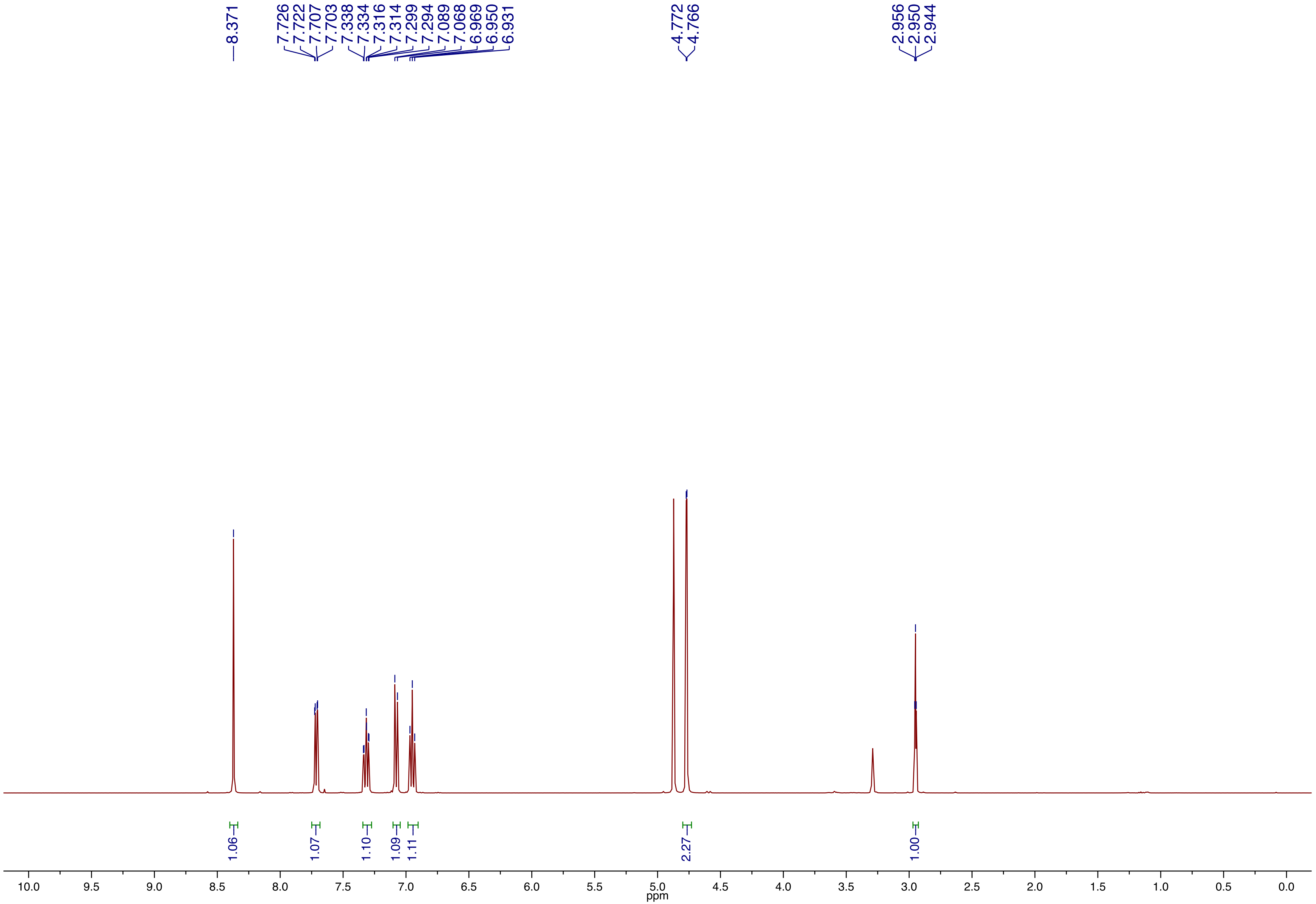

^{13}C NMR (101 MHz, CD_3OD)

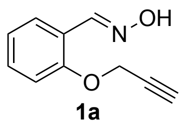

1a

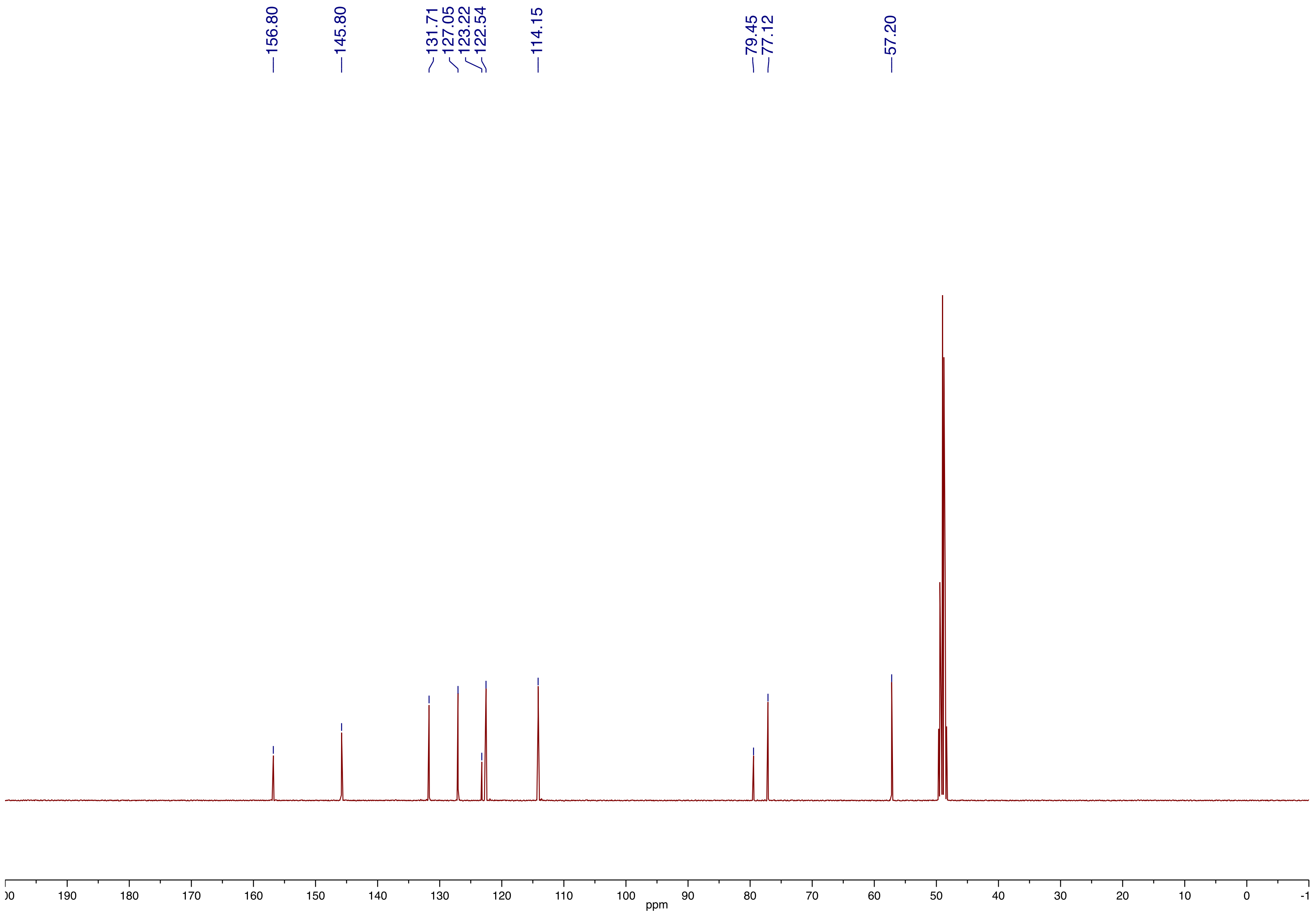

^{1}H NMR (400 MHz, $CDCl_3$)

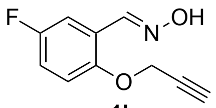

1b

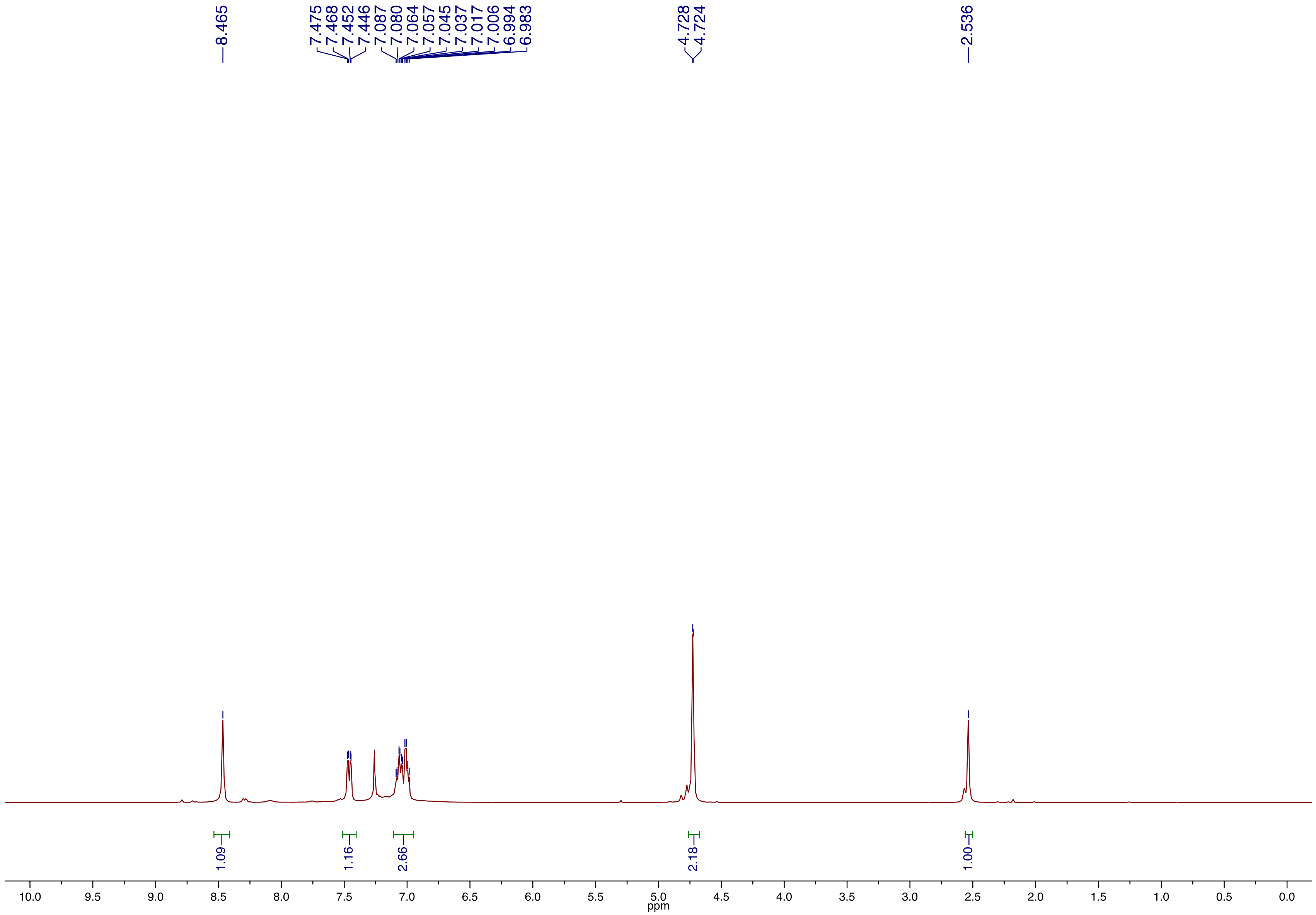

^{13}C NMR (101 MHz, $CDCl_3$)

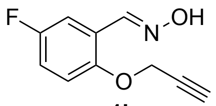

1b

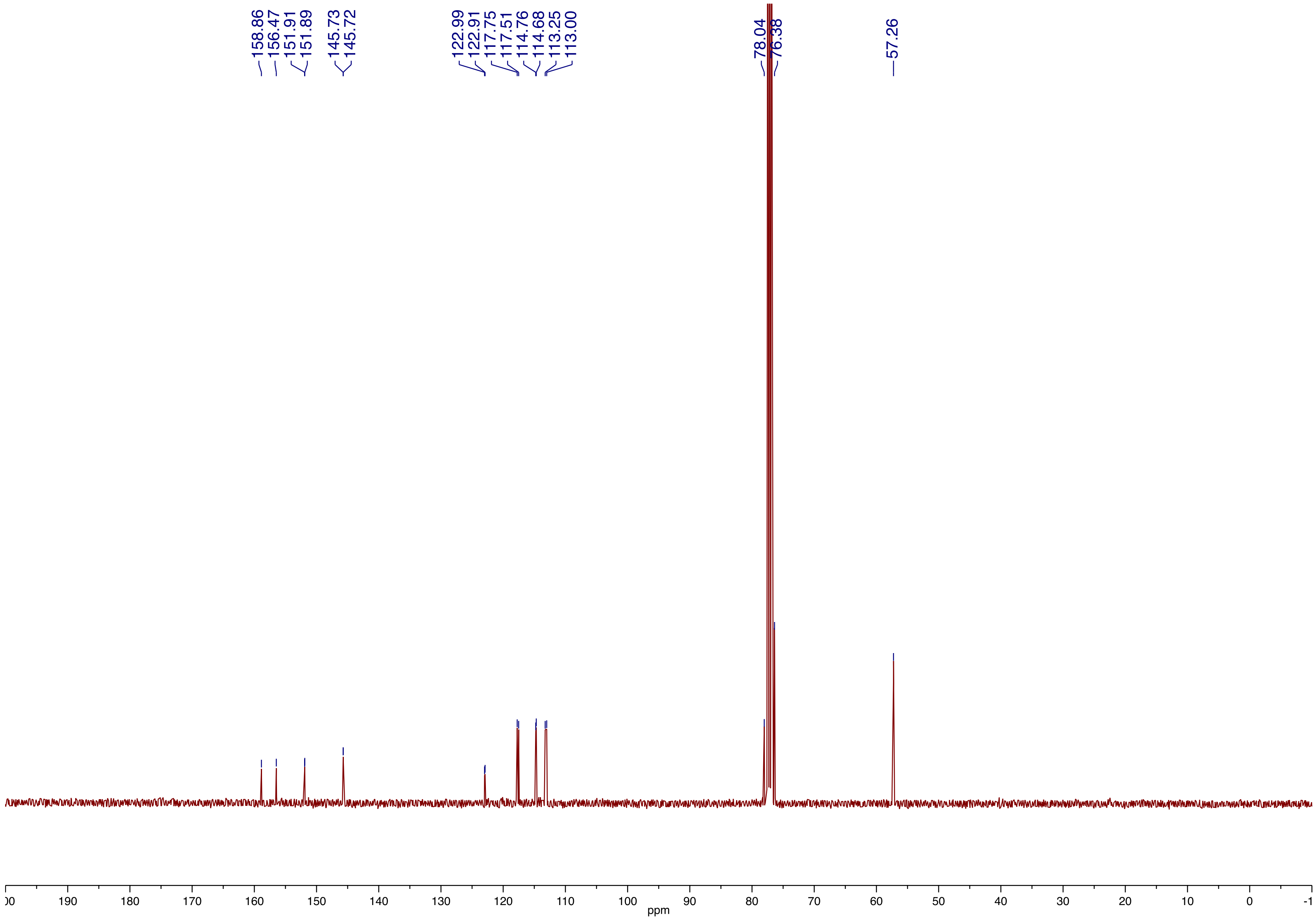

19F NMR (376 MHz, $CDCl_3$)

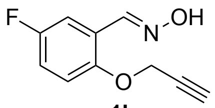

1b

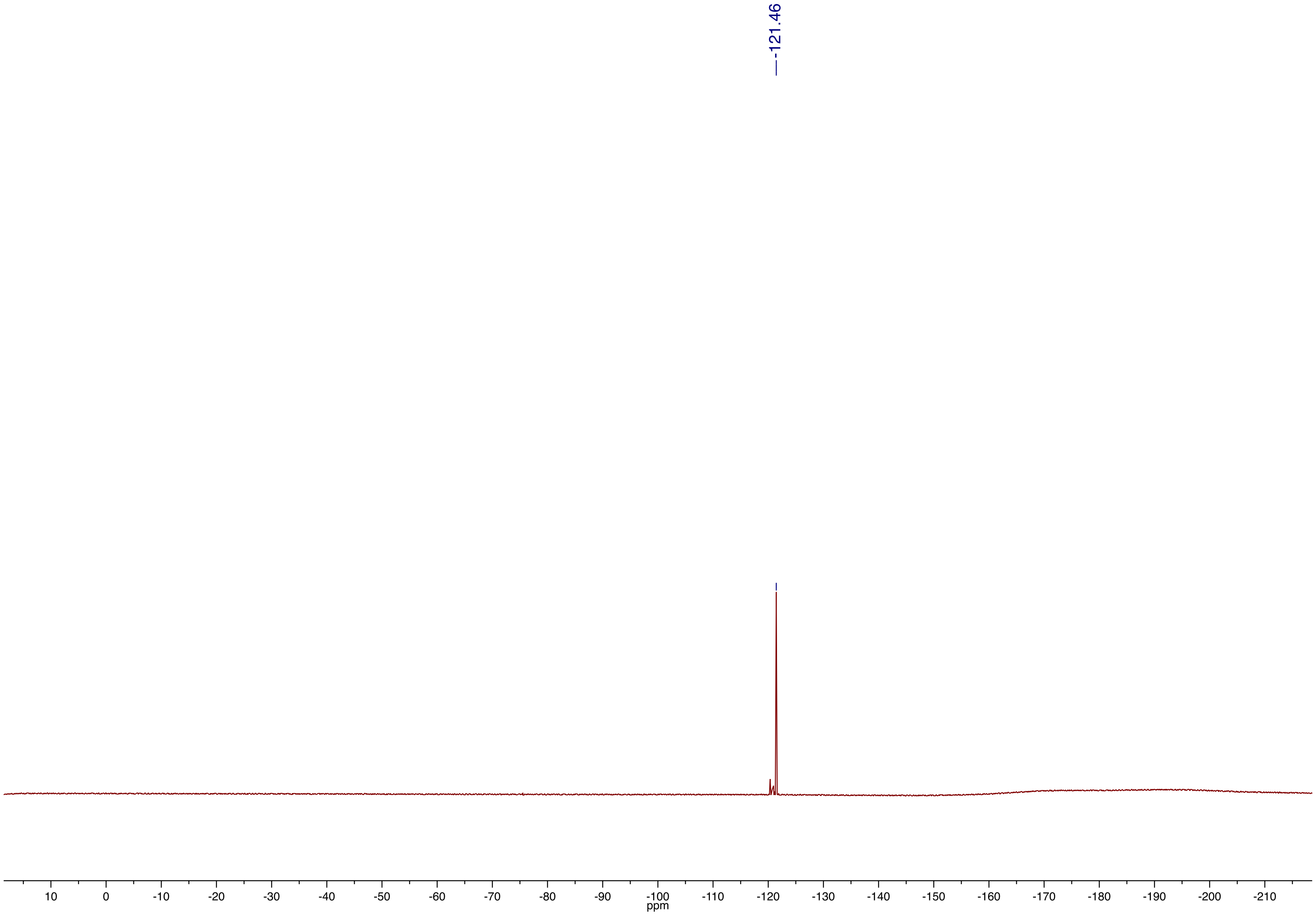

¹H NMR (400 MHz, $CDCl_3$)

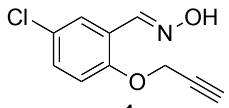

1c

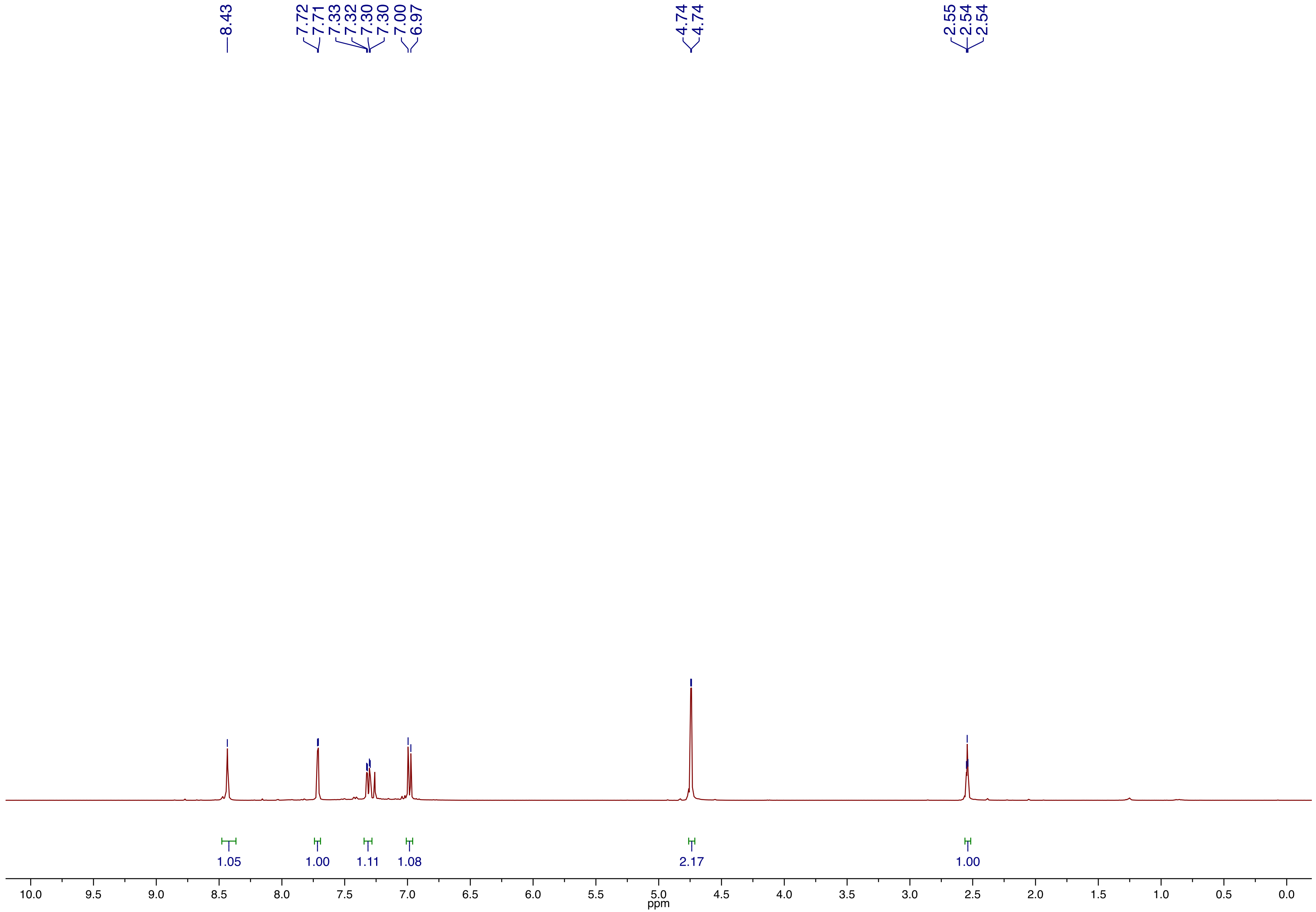

^{13}C NMR (101 MHz, $CDCl_3$)

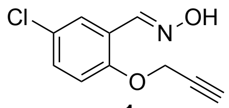

1c

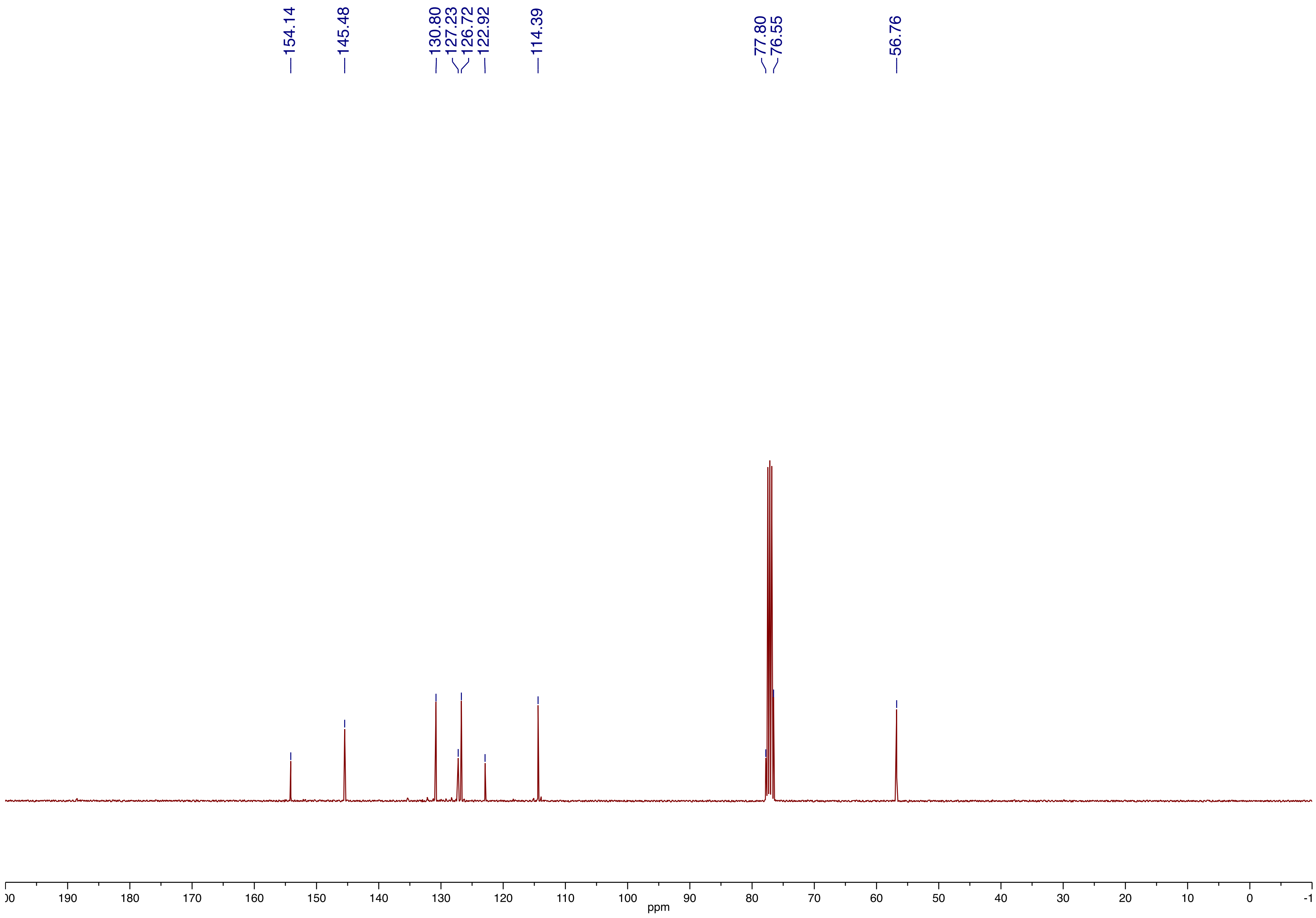

^{1}H NMR (500 MHz, $CDCl_3$)

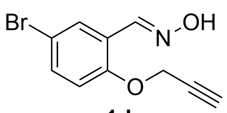

1d

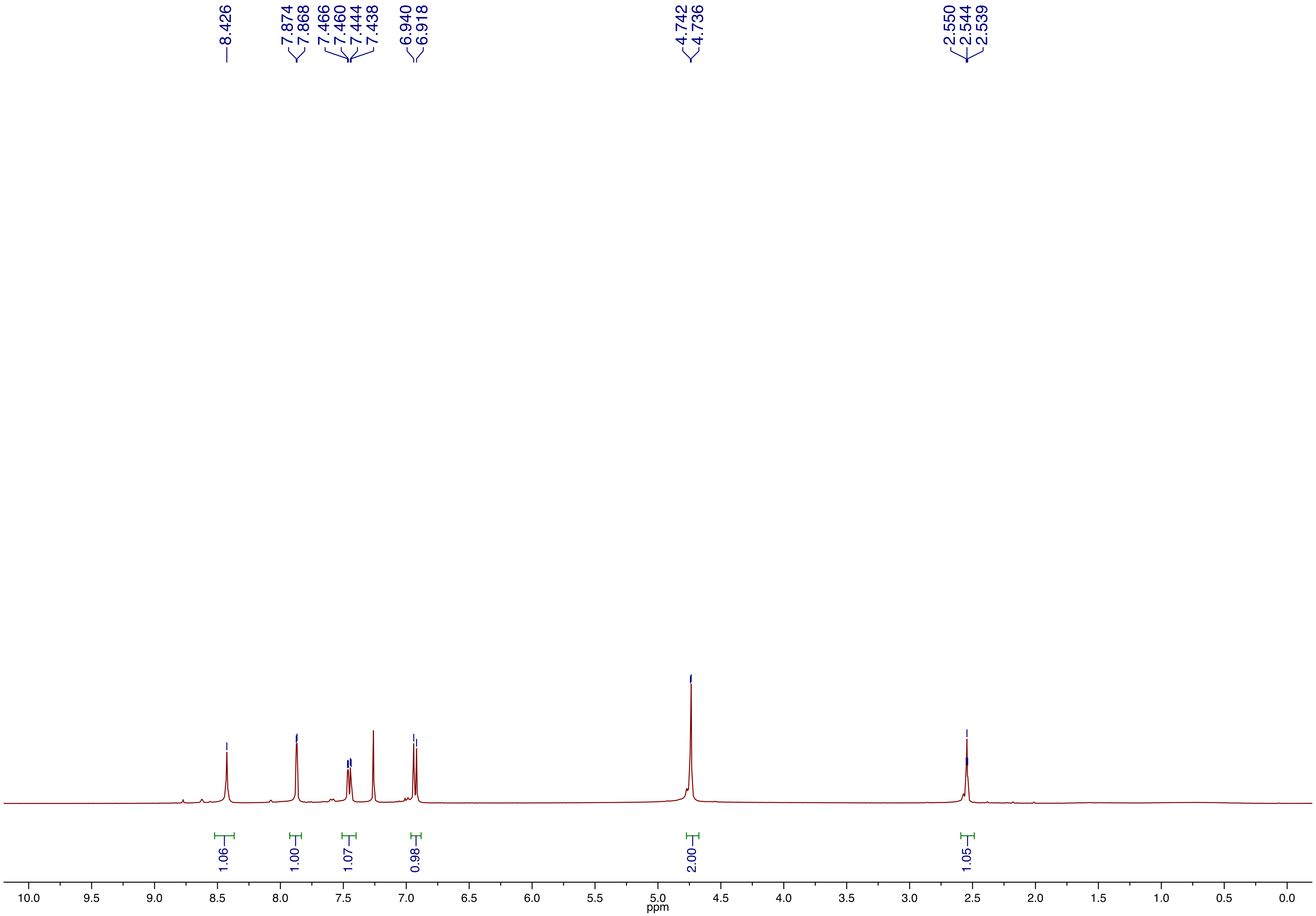

^{13}C NMR (101 MHz, $CDCl_3$)

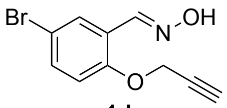

1d

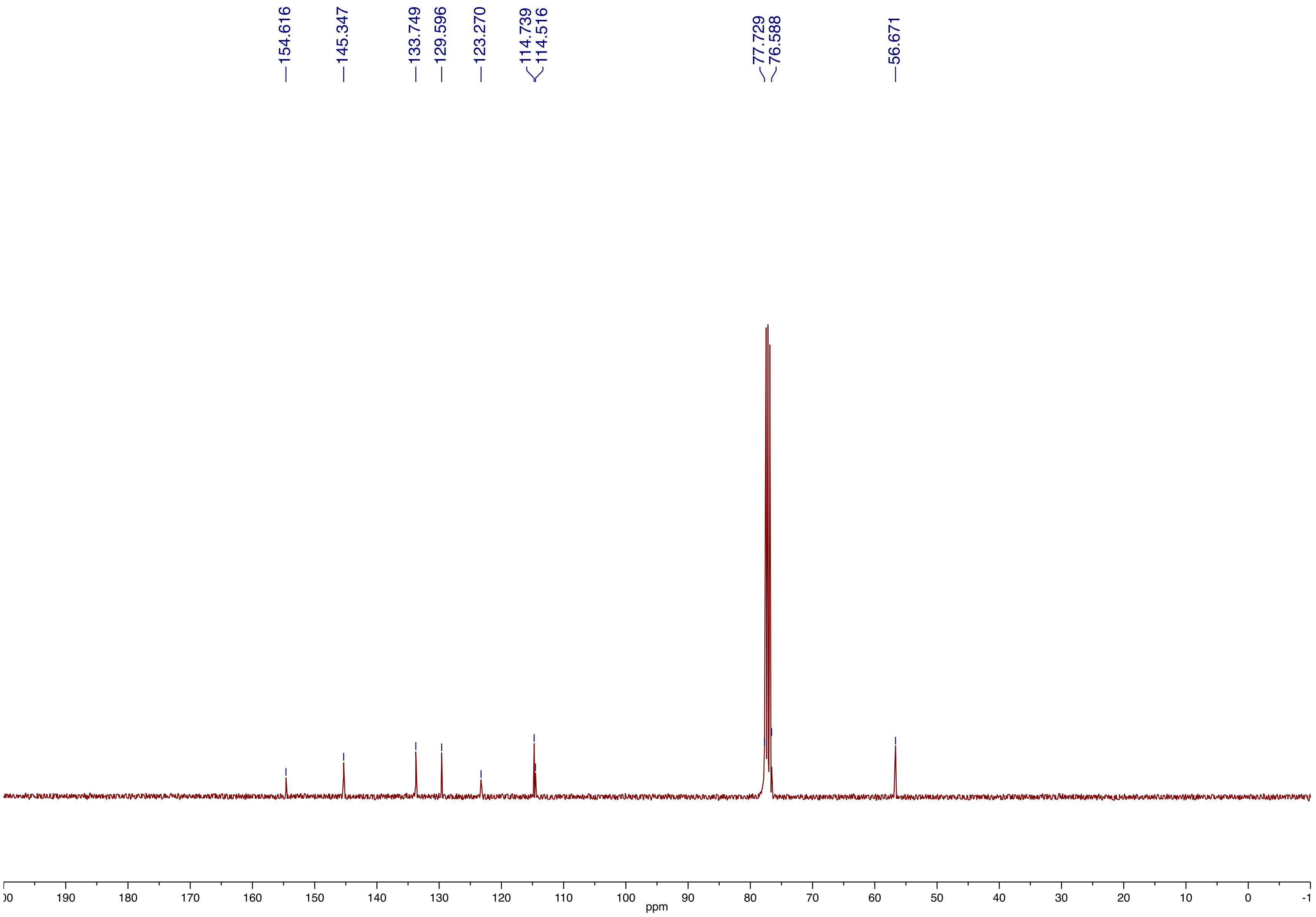

^{1}H NMR (400 MHz, $CDCl_3$)

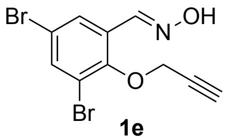

1e

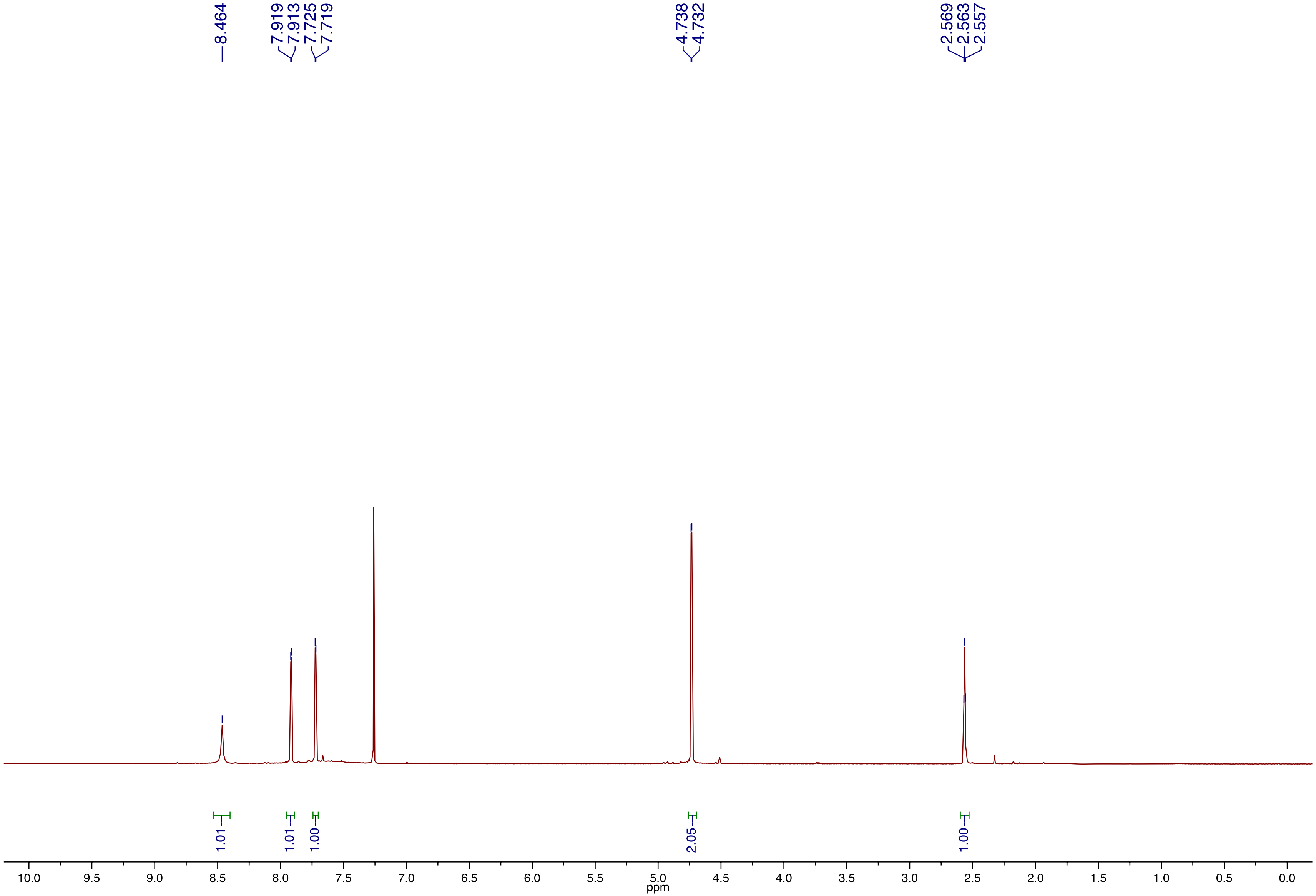

^{13}C NMR (101 MHz, $CDCl_3$)

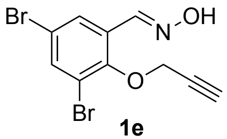

1e

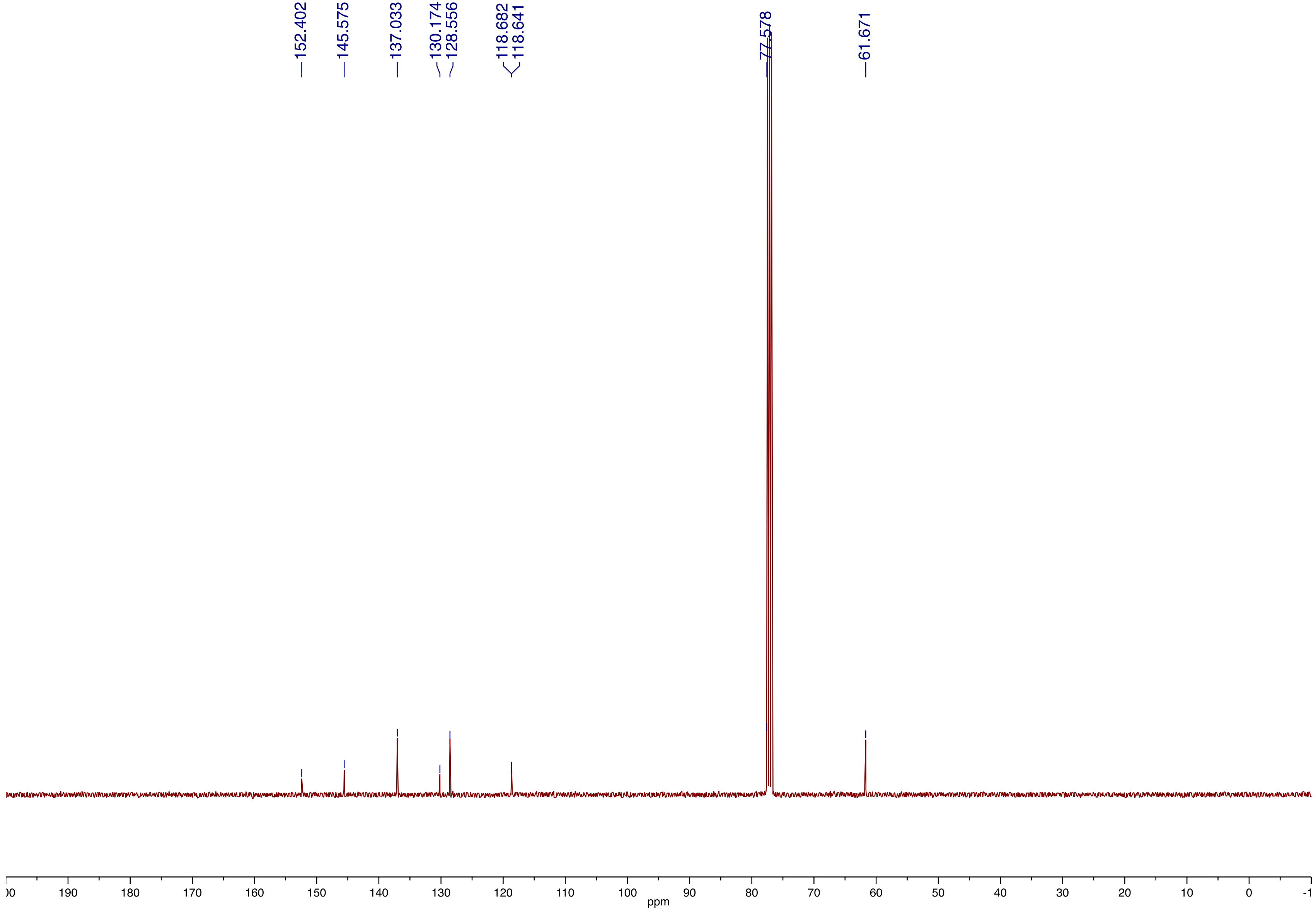

^{1}H NMR (400 MHz, CD_3OD)

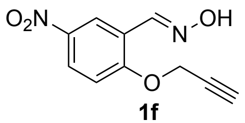

1f

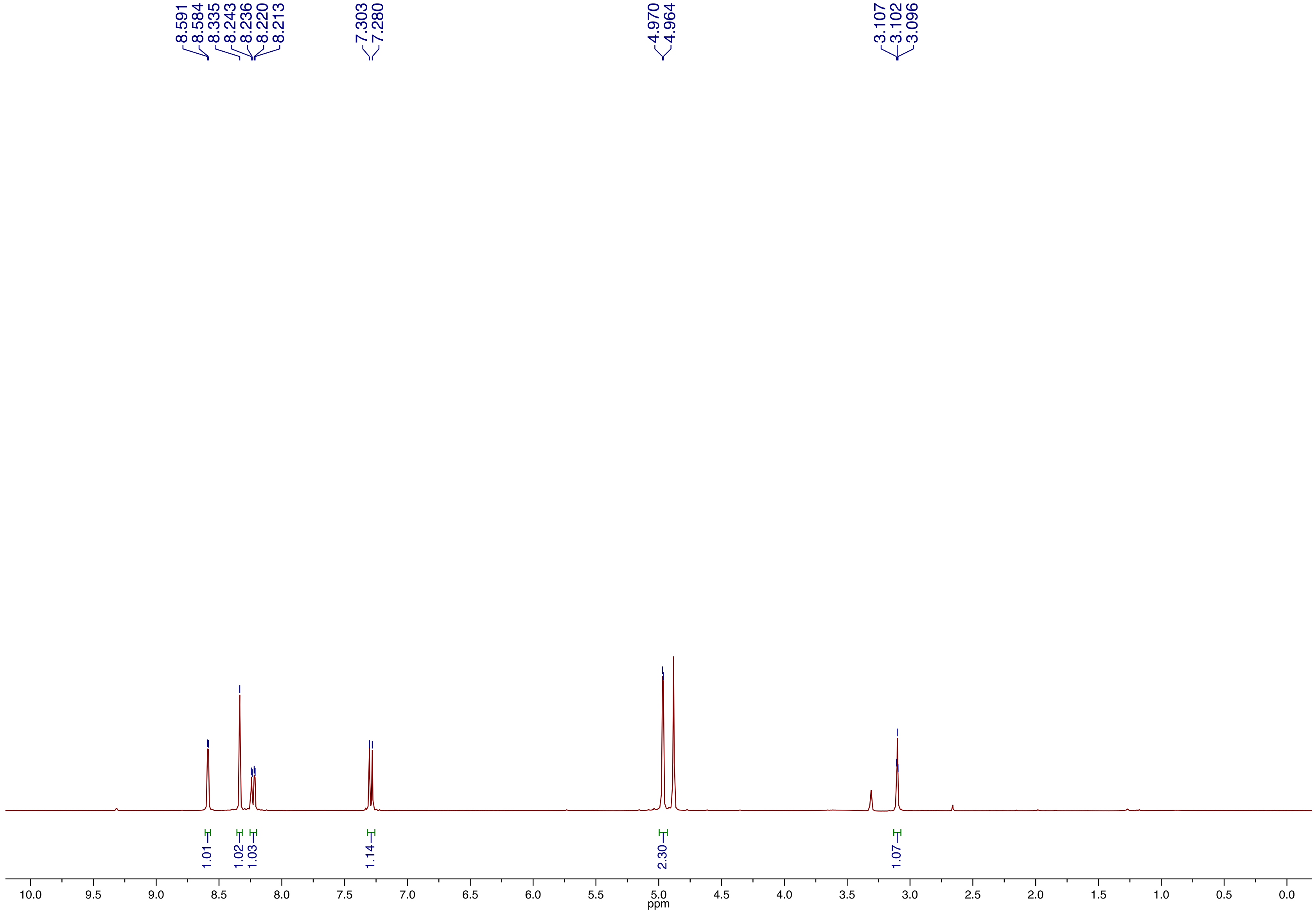

^{13}C NMR (101 MHz, CD_3OD)

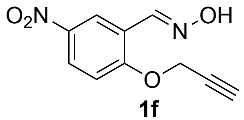

1f

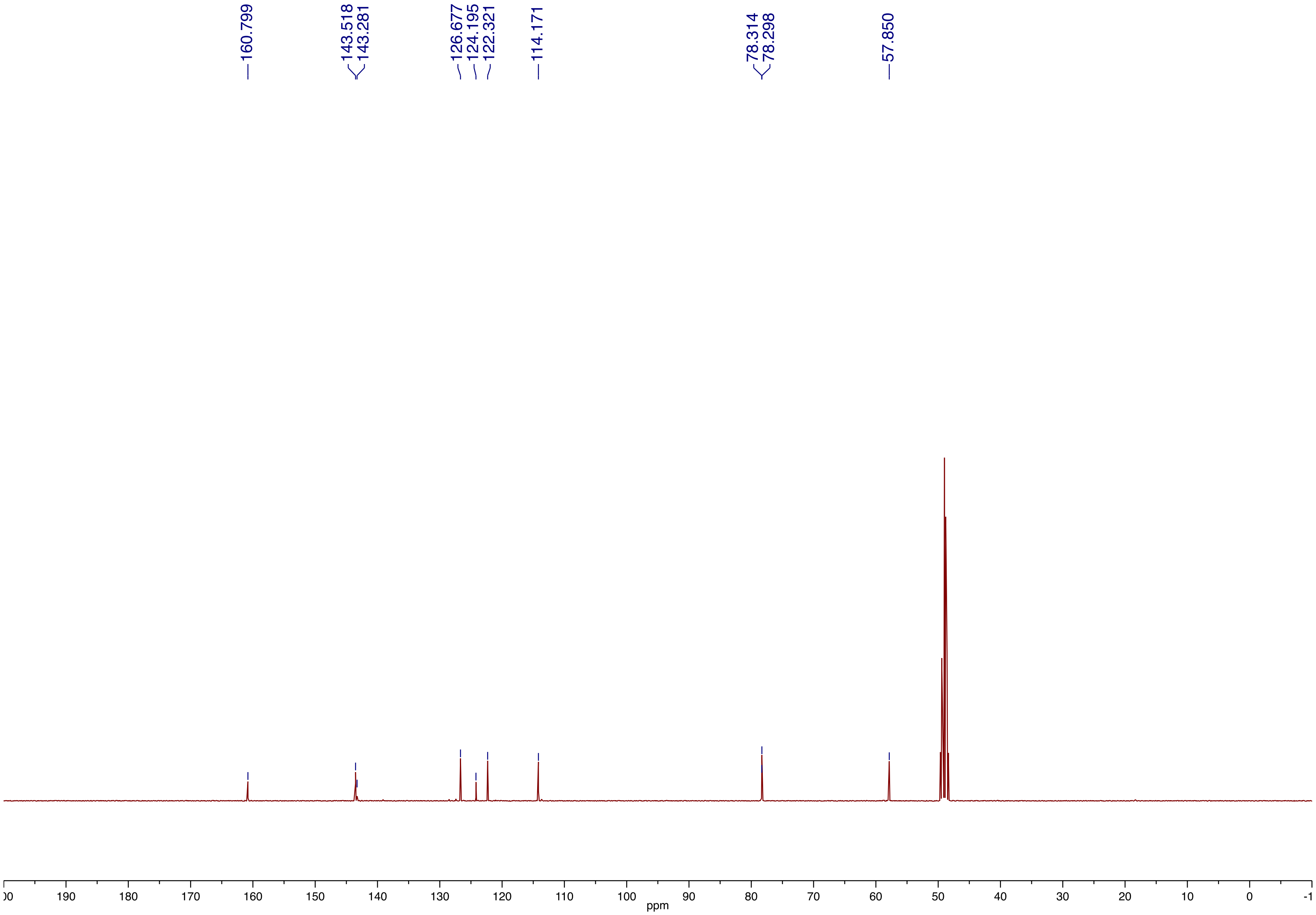

^{1}H NMR (400 MHz, $CDCl_3$)

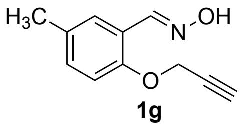

1g

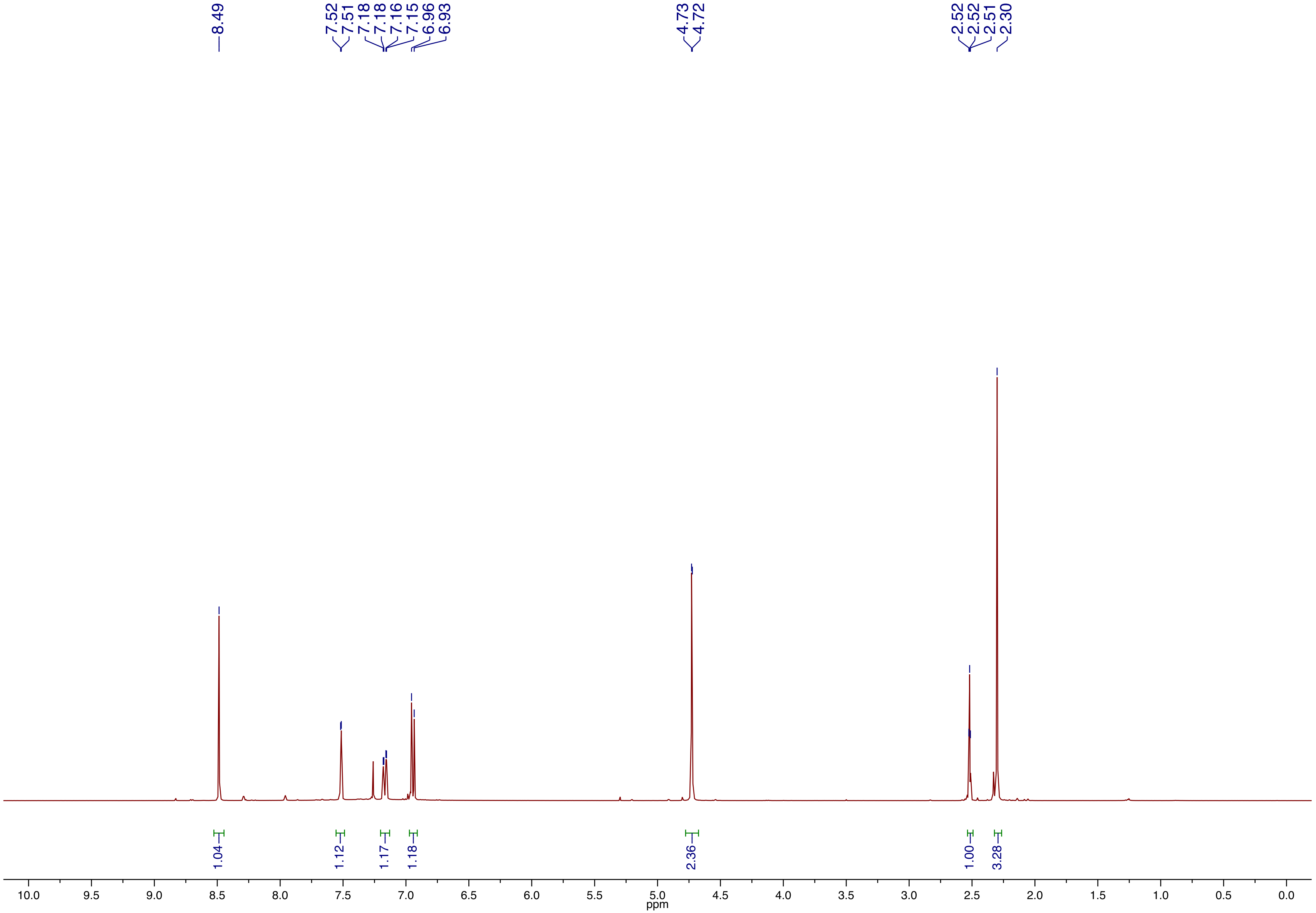

^{13}C NMR (101 MHz, $CDCl_3$)

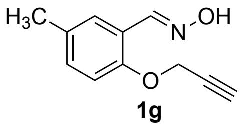

1g

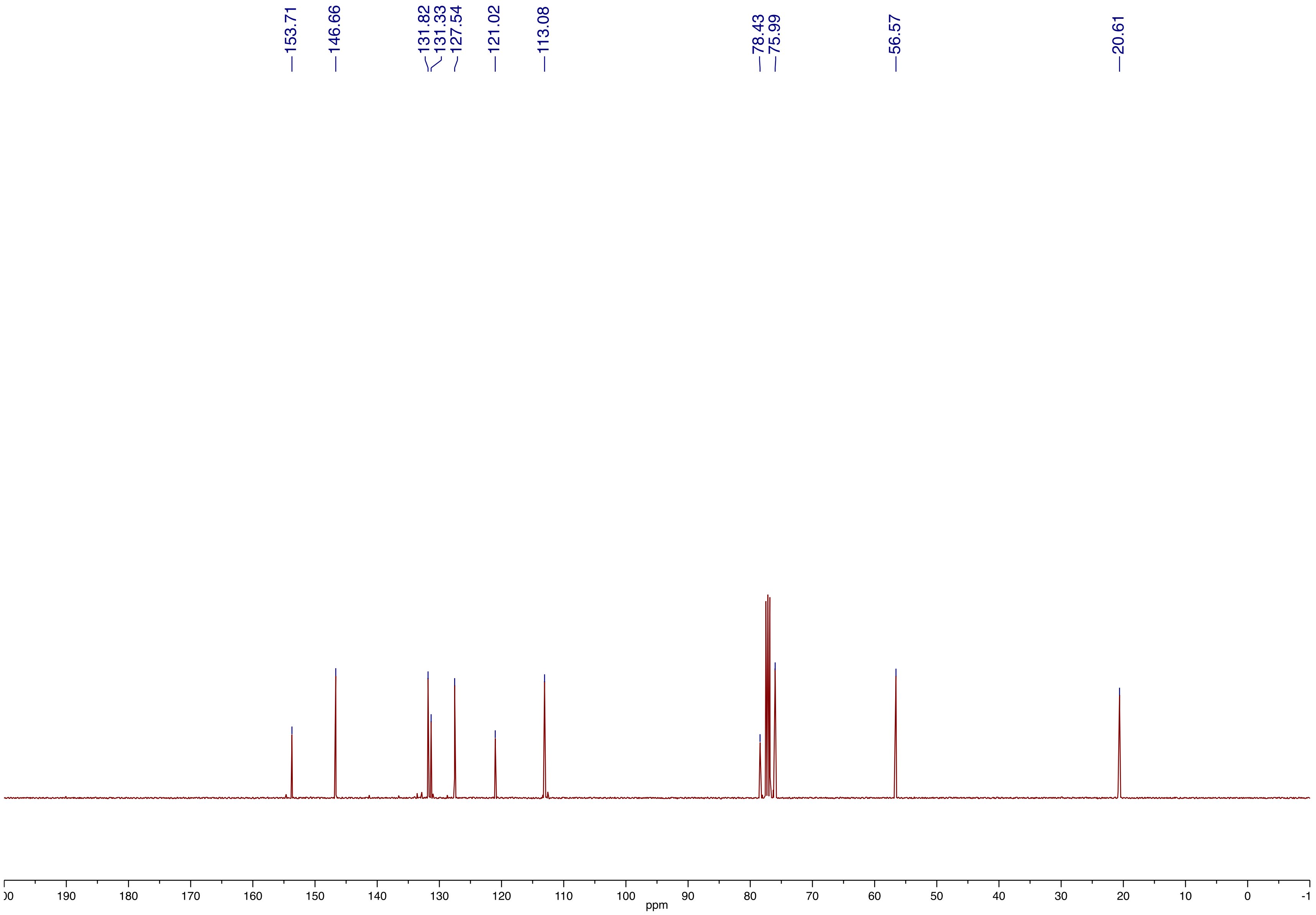

^{1}H NMR (400 MHz, CD_3OD)

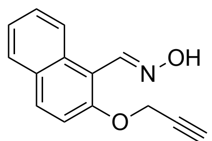

1h

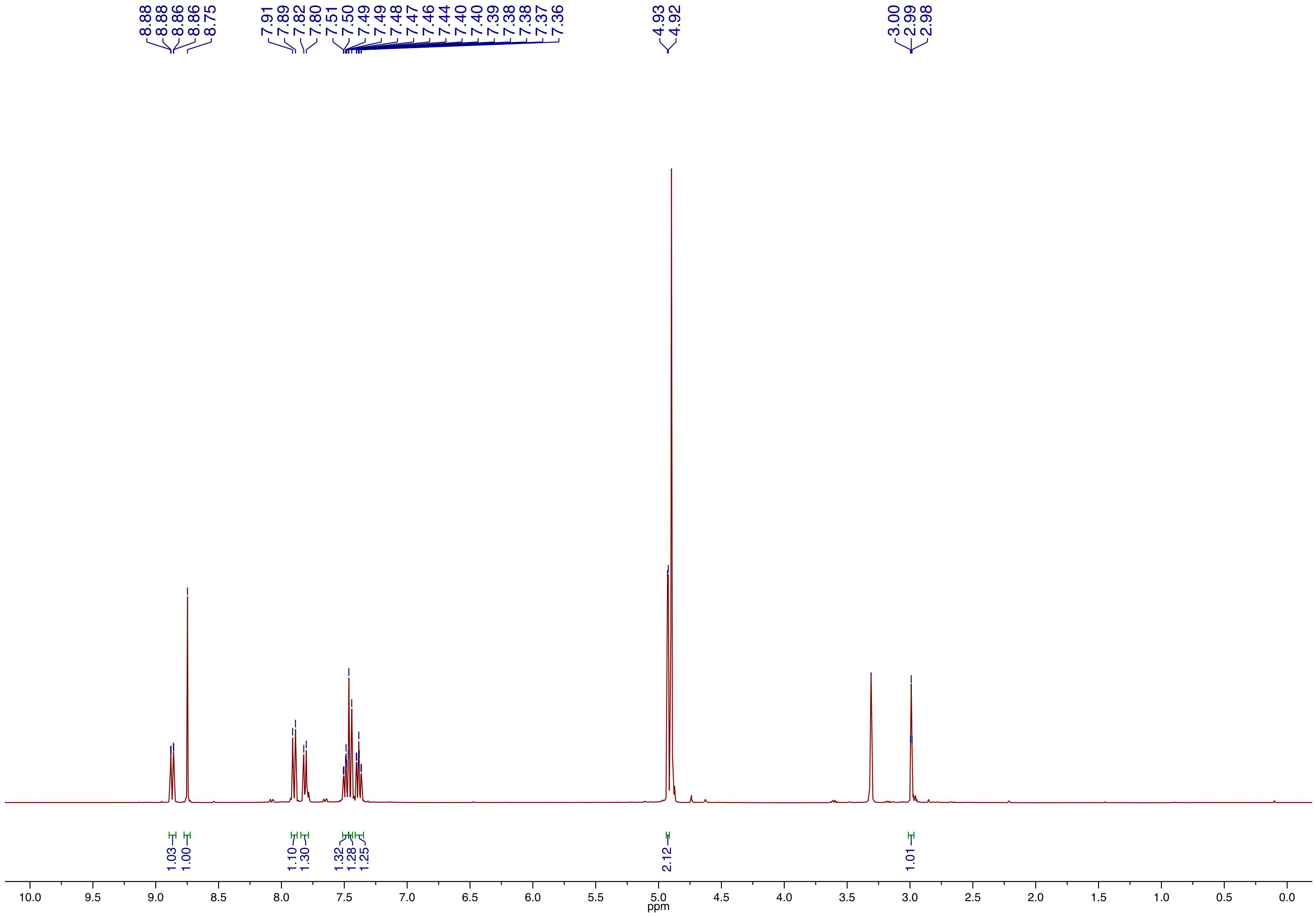

^{13}C NMR (101 MHz, CD_3OD)

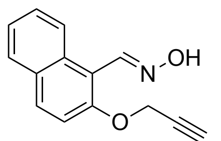

1h

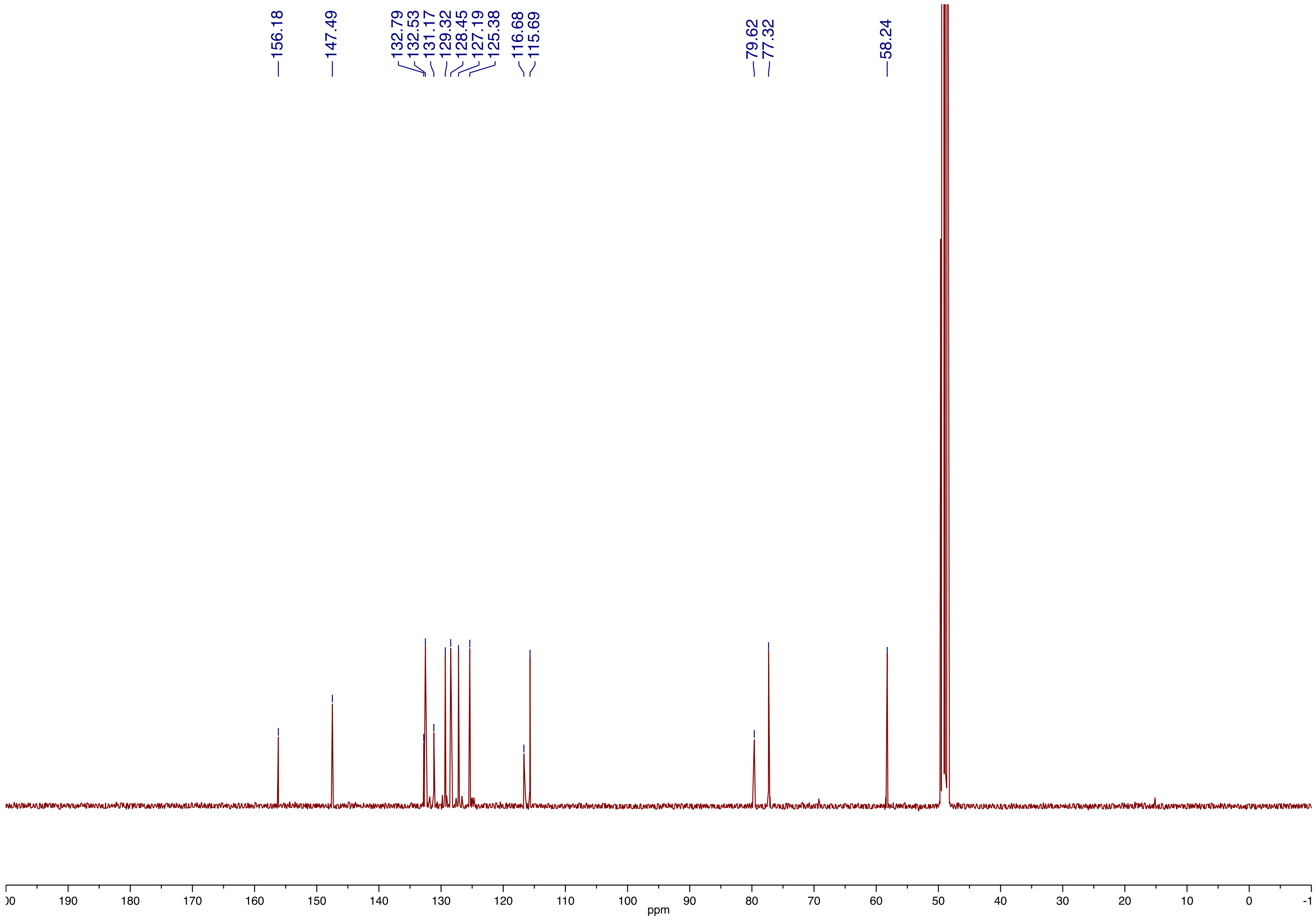

^1H NMR (400 MHz, DMSO-d_6)

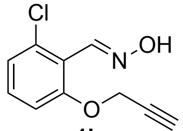

1i

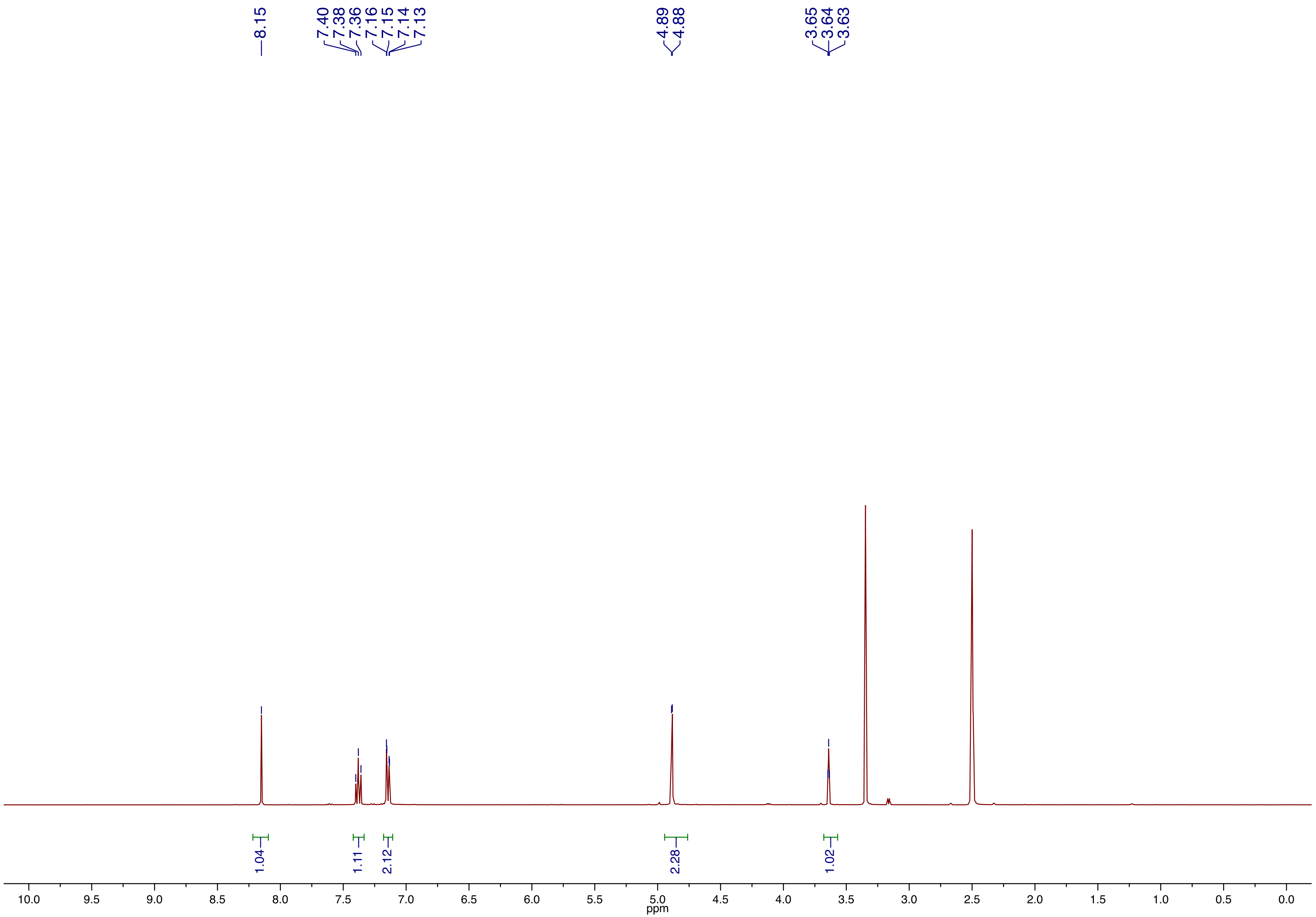

^{13}C NMR (101 MHz, DMSO-d_6)

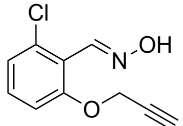

1i

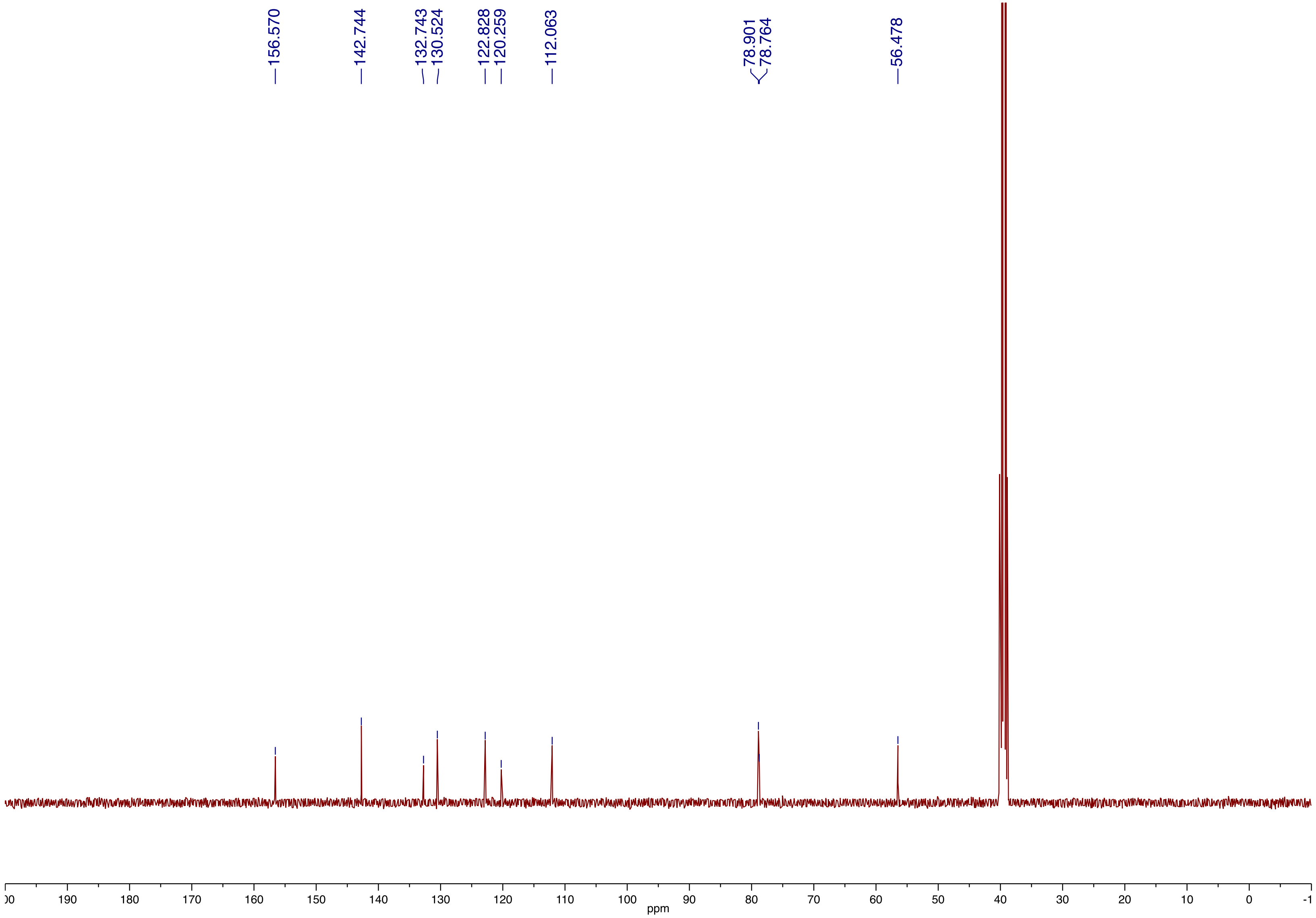

¹H NMR (500 MHz, $CDCl_3$)

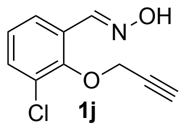
1j

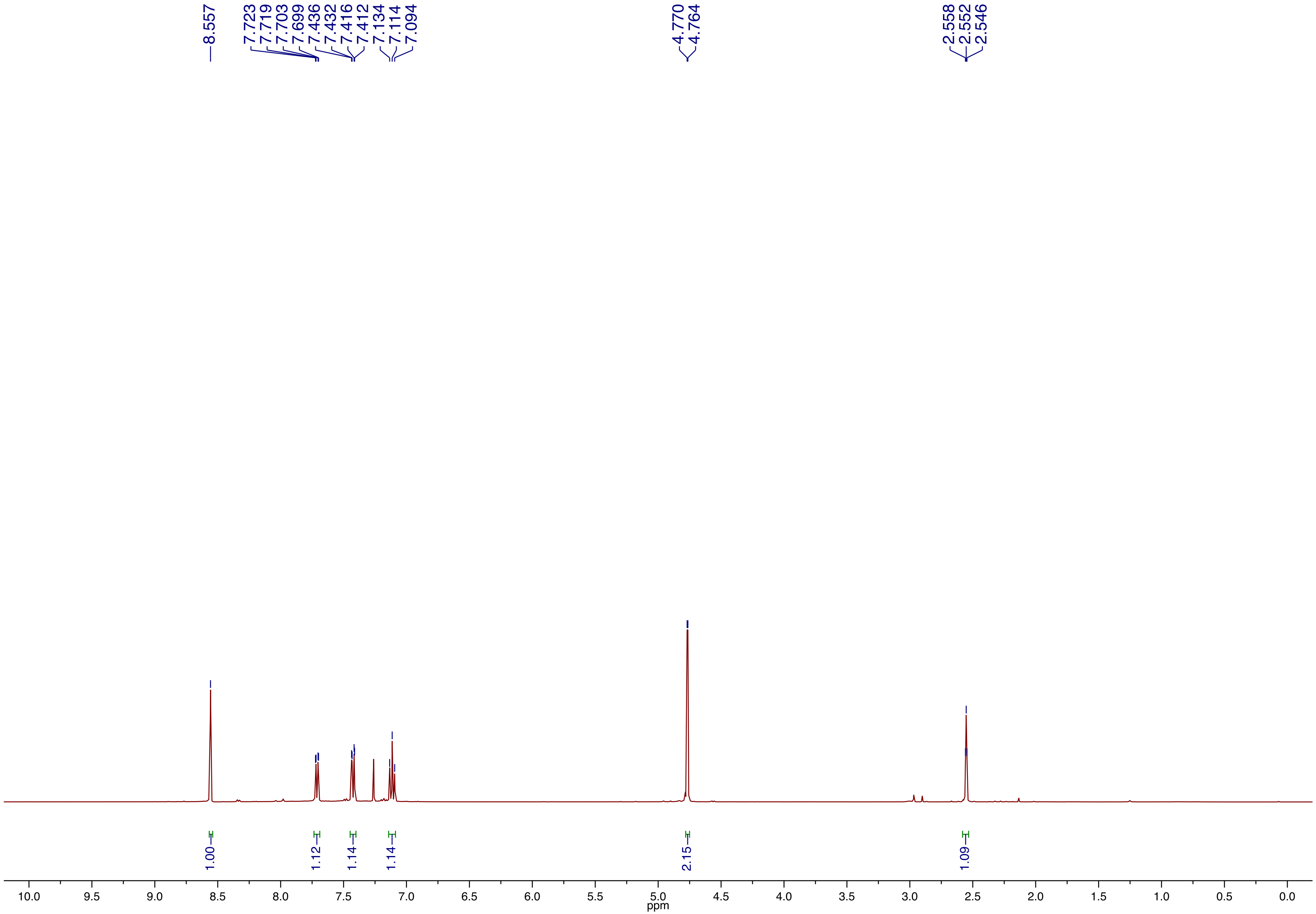

^{13}C NMR (101 MHz, $CDCl_3$)

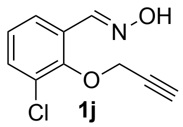

1j

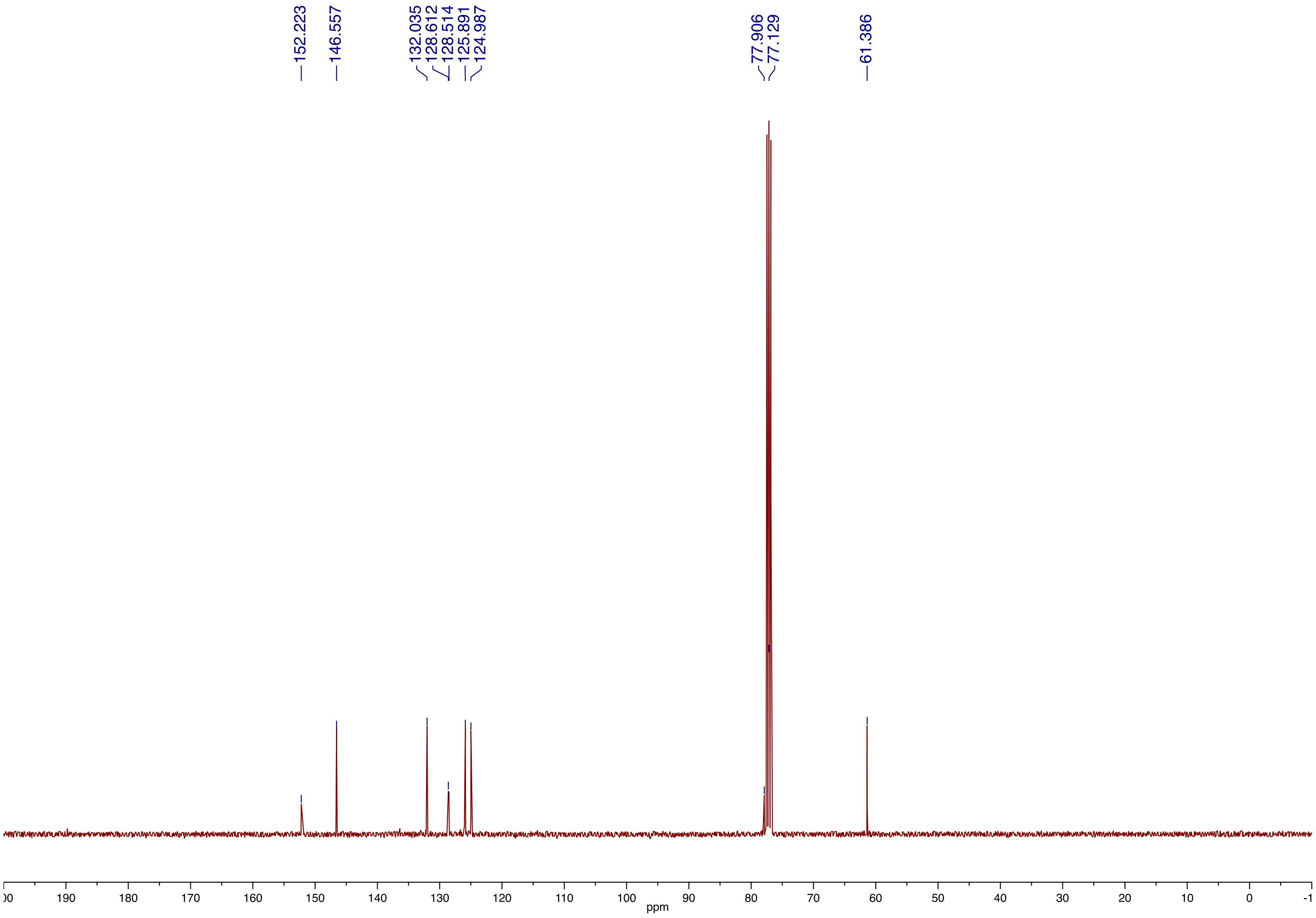

^{1}H NMR (400 MHz, $CDCl_3$)

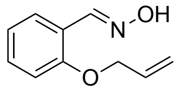

1k

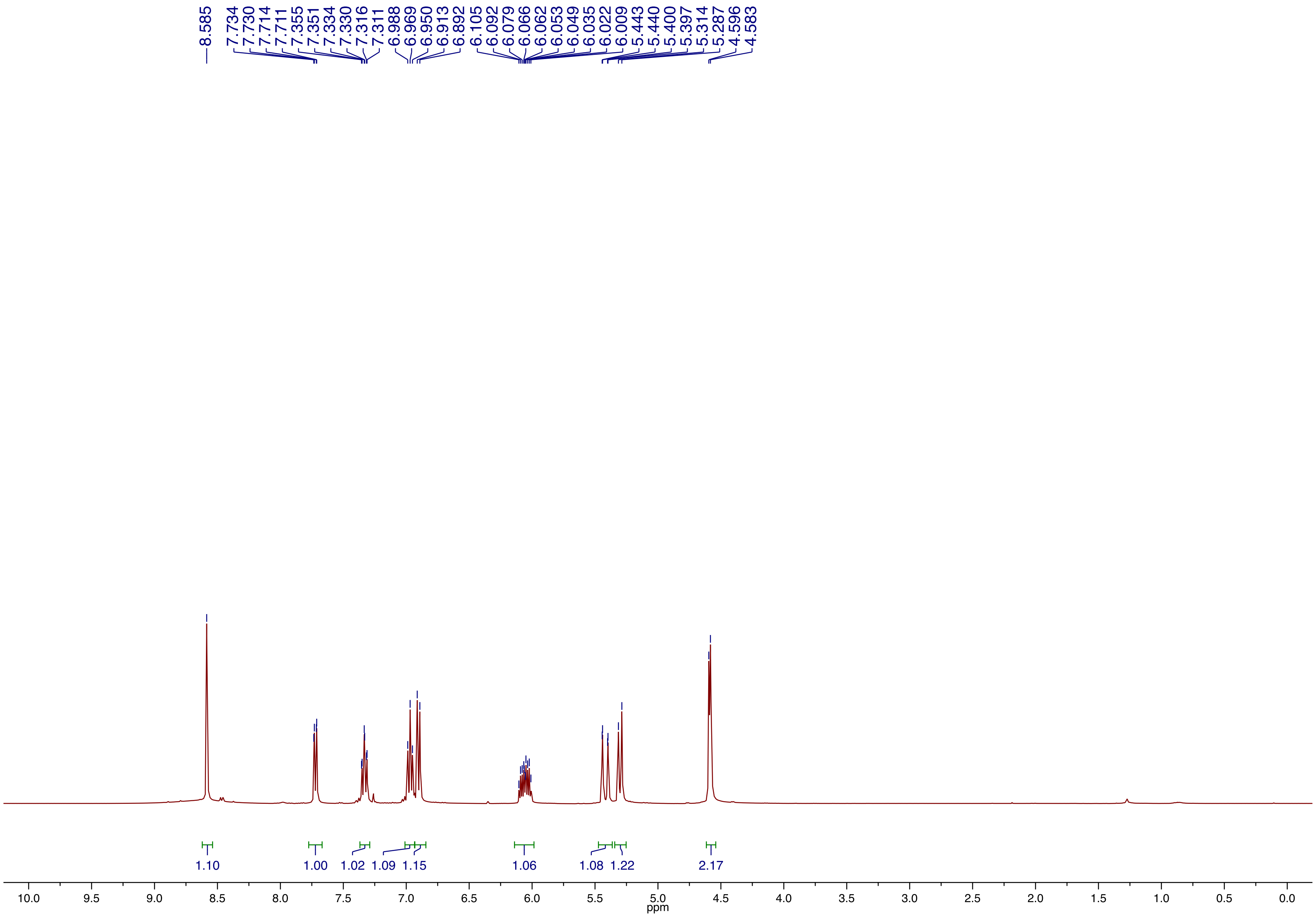

^{13}C NMR (101 MHz, $CDCl_3$)

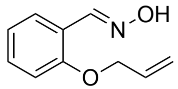

1k

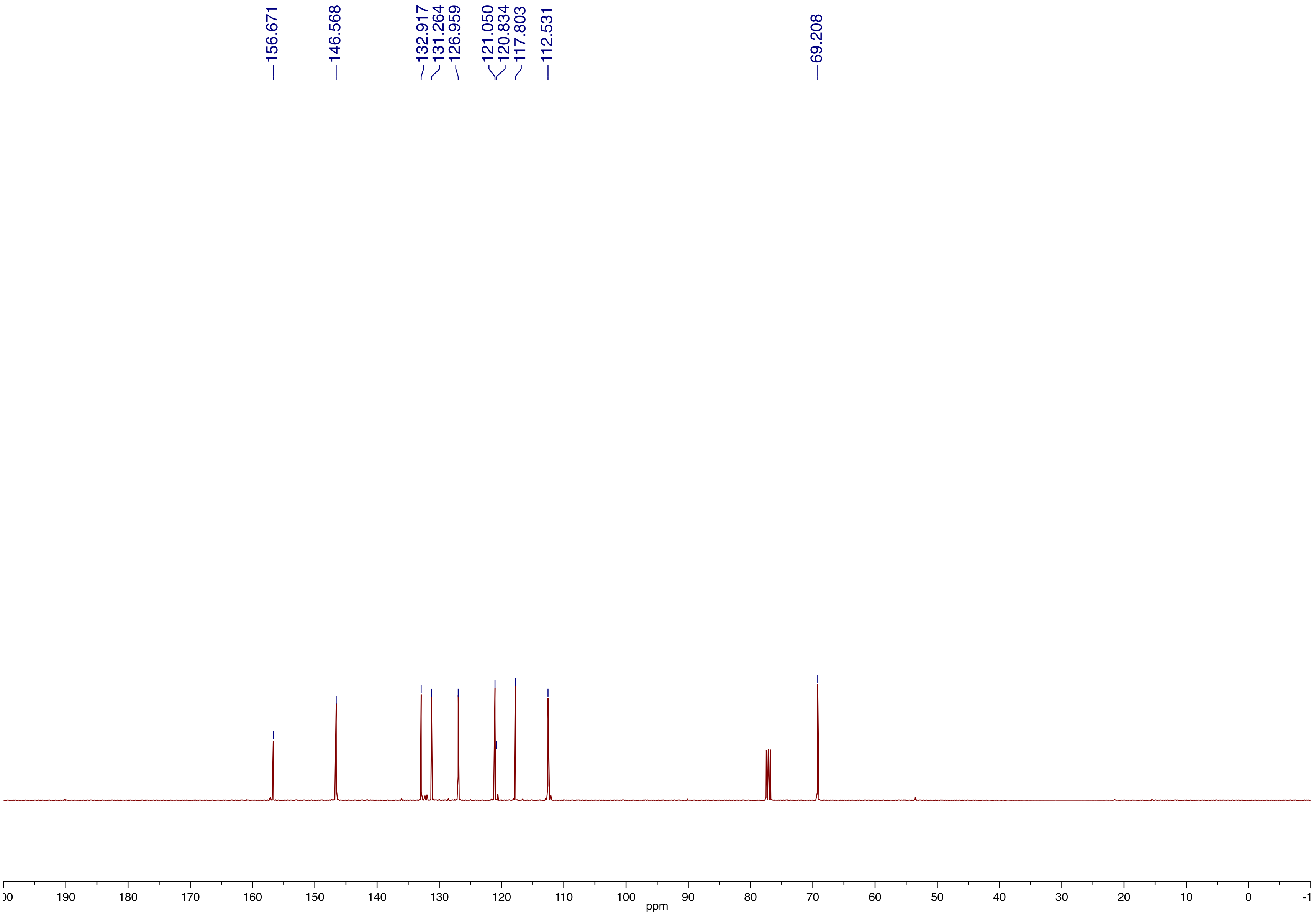

^1H NMR (400 MHz, $CDCl_3$)

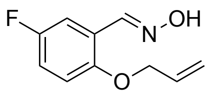

1l

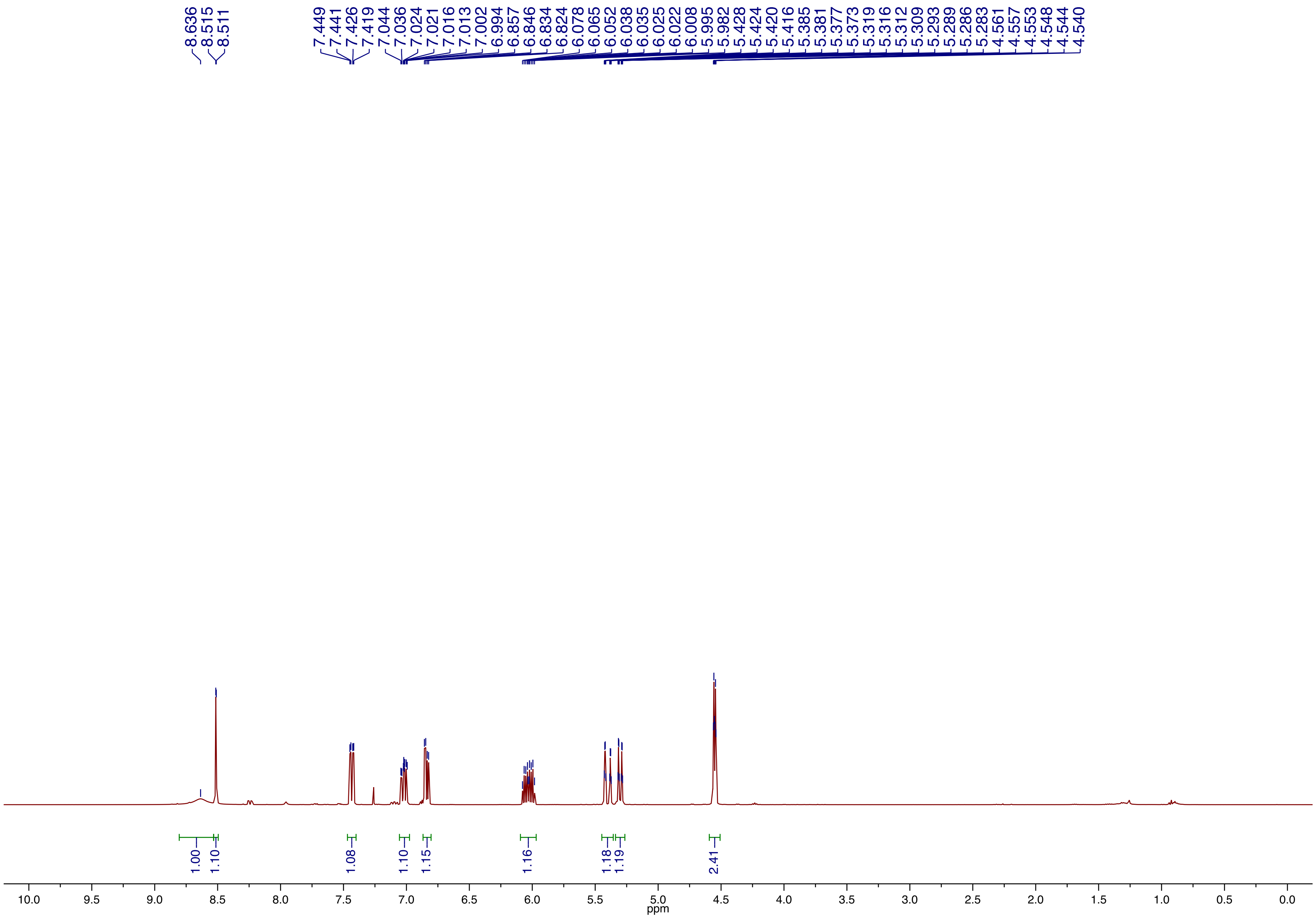

^{13}C NMR (101 MHz, $CDCl_3$)

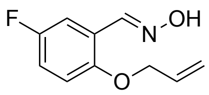

1l

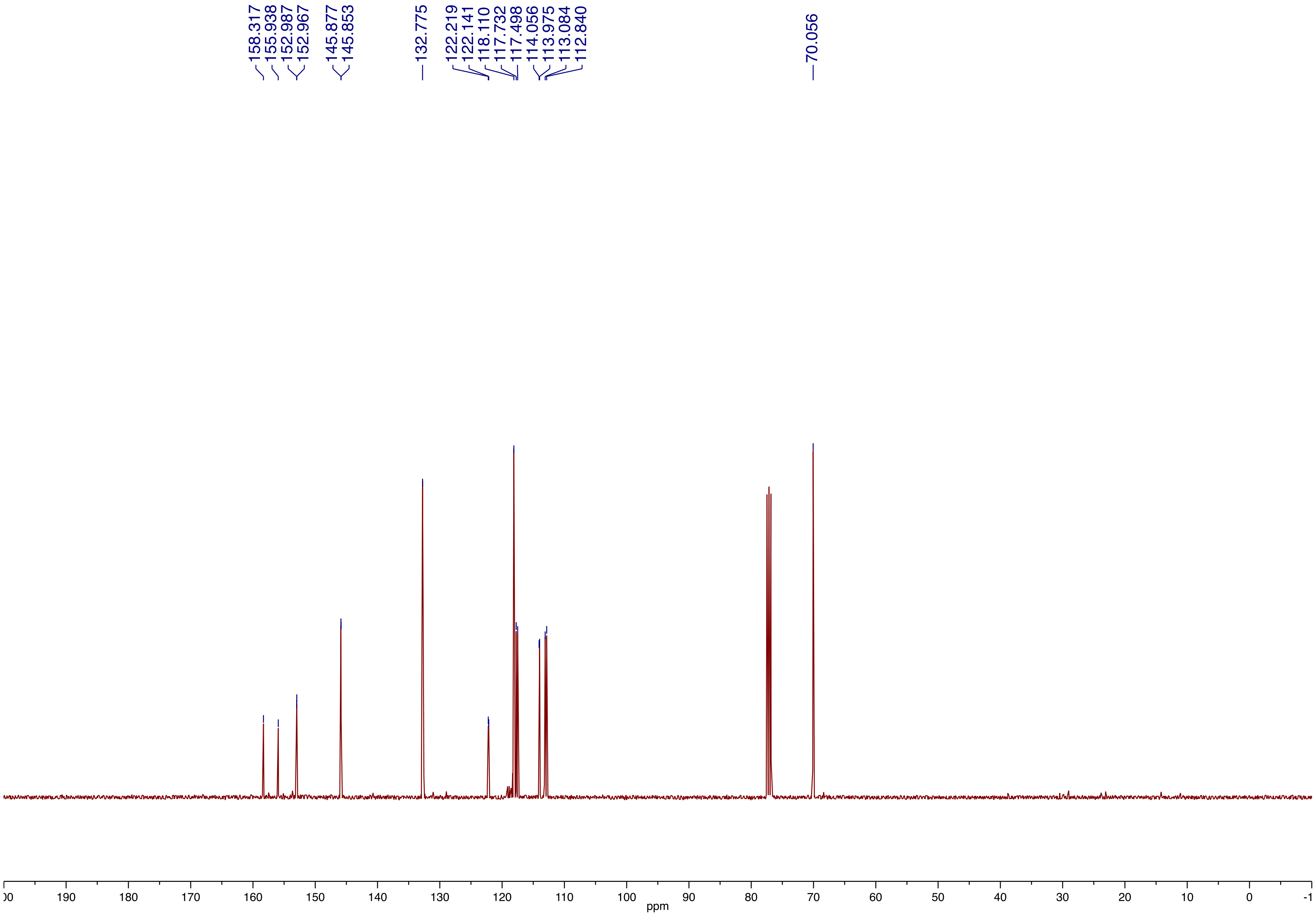

^{19}F NMR (376 MHz, $CDCl_3$)

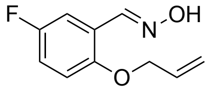

1l

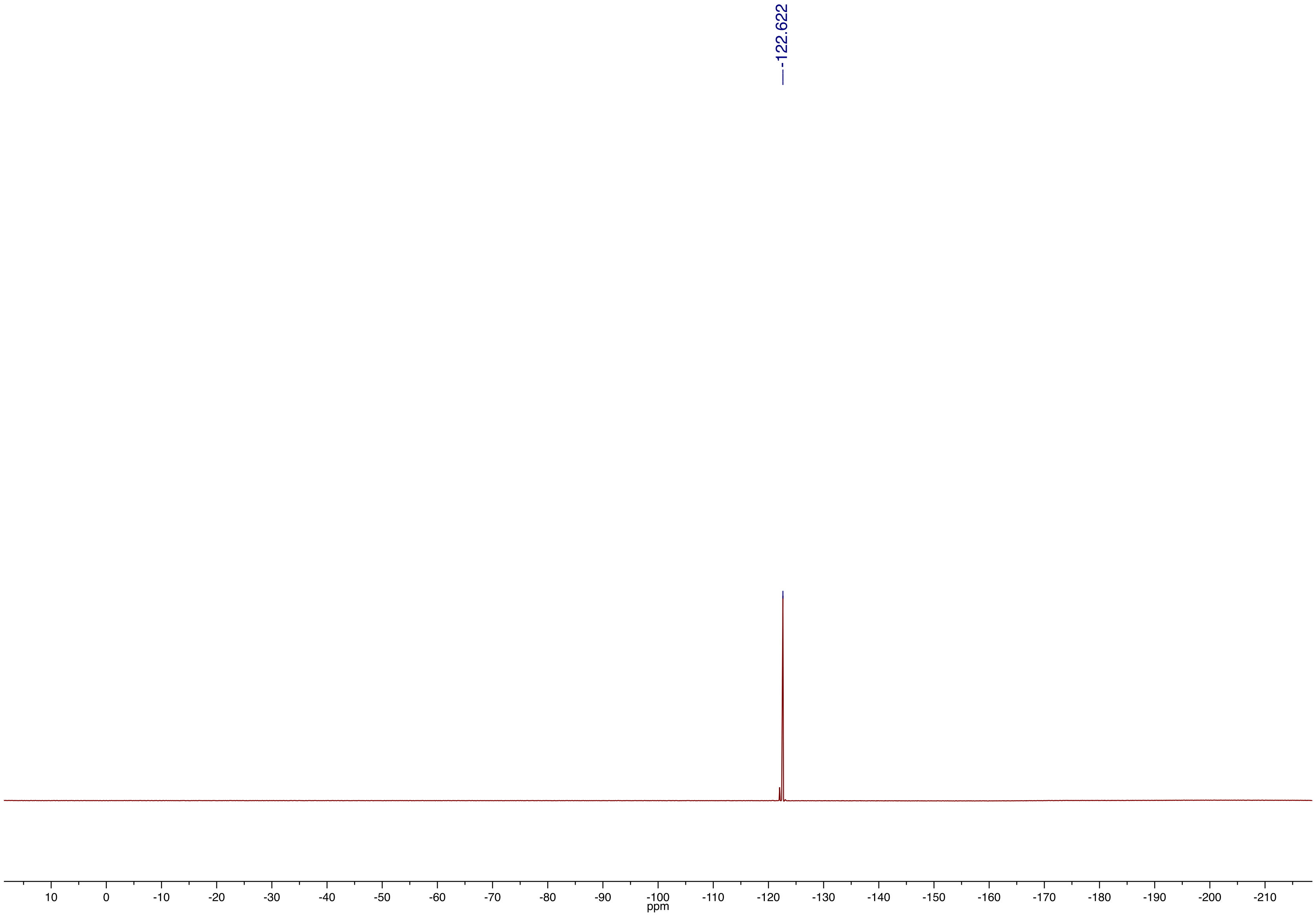

^1H NMR (400 MHz, $CDCl_3$)

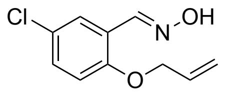

1m

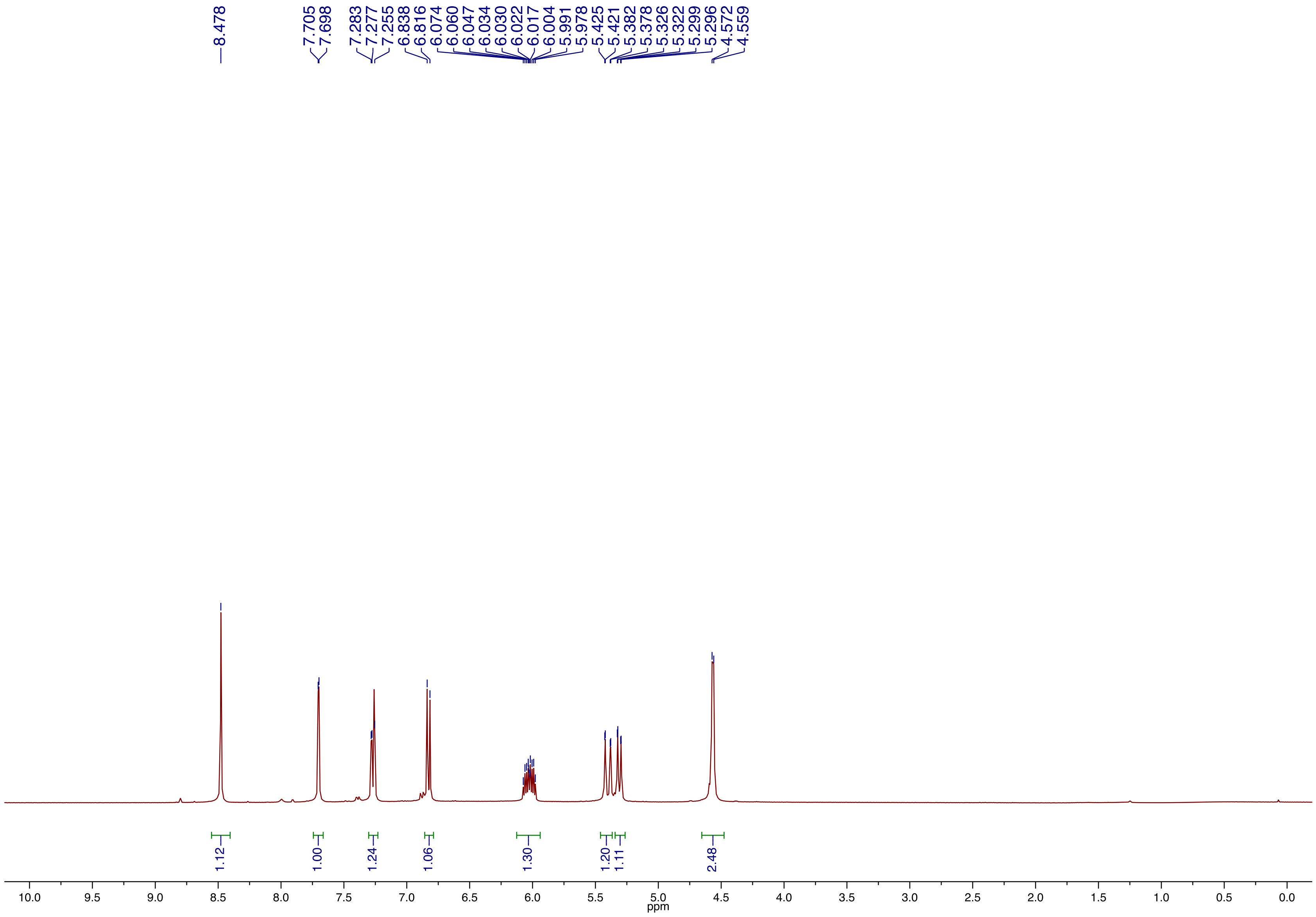

^{13}C NMR (101 MHz, $CDCl_3$)

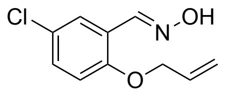

1m

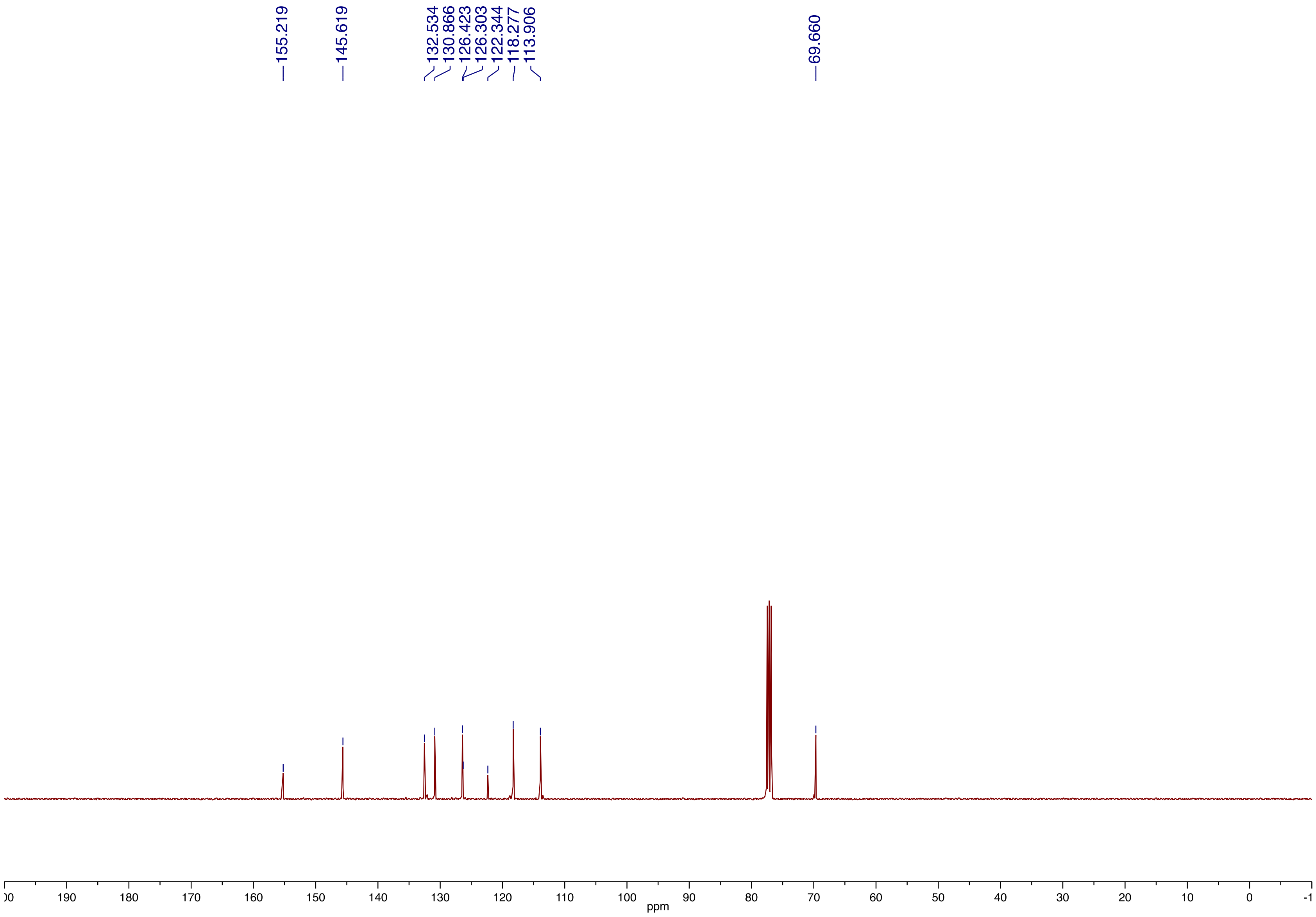

^{1}H NMR (400 MHz, $CDCl_3$)

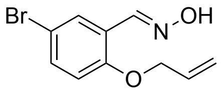

1n

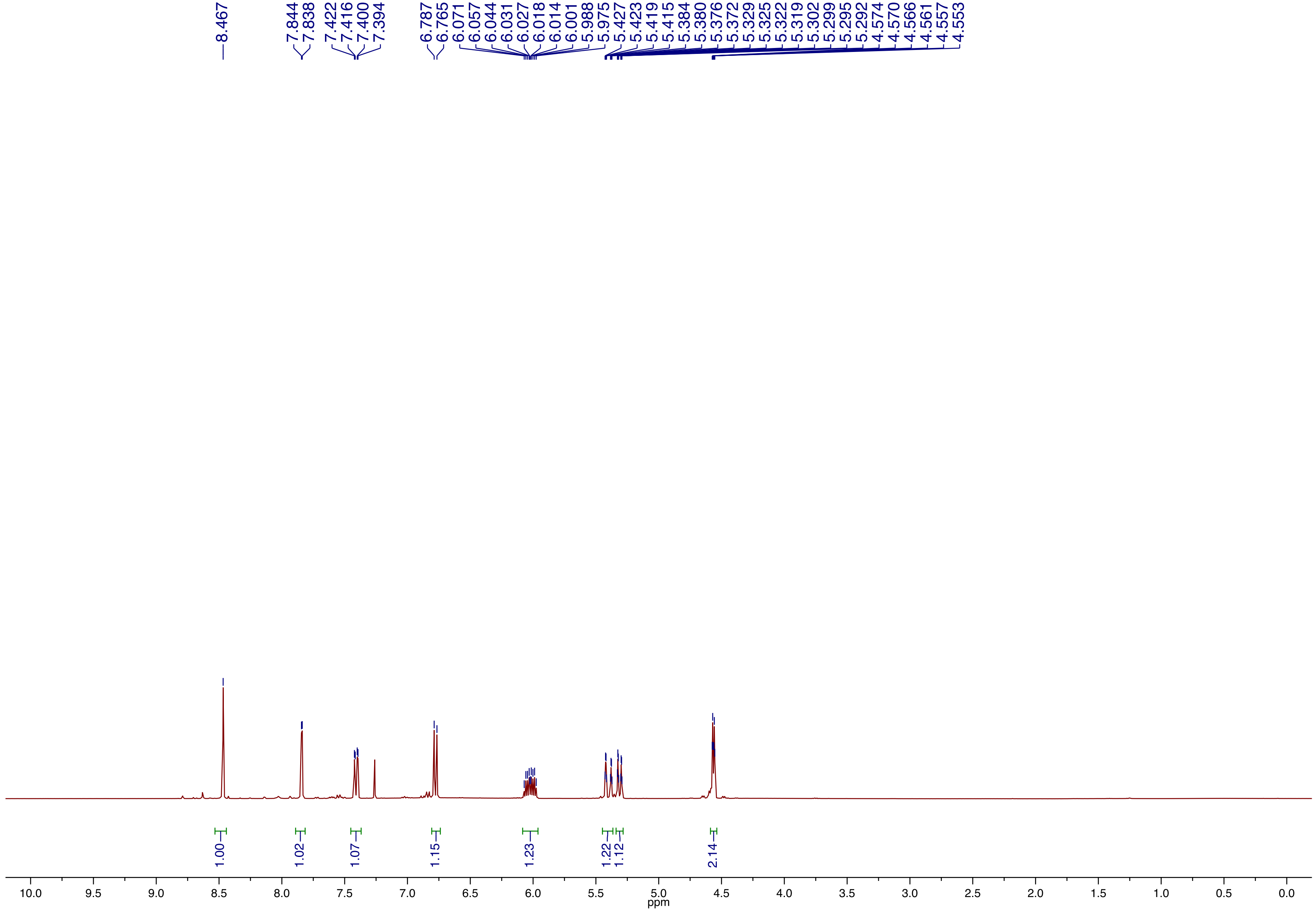

^{13}C NMR (101 MHz, $CDCl_3$)

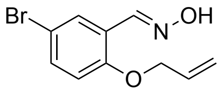

1n

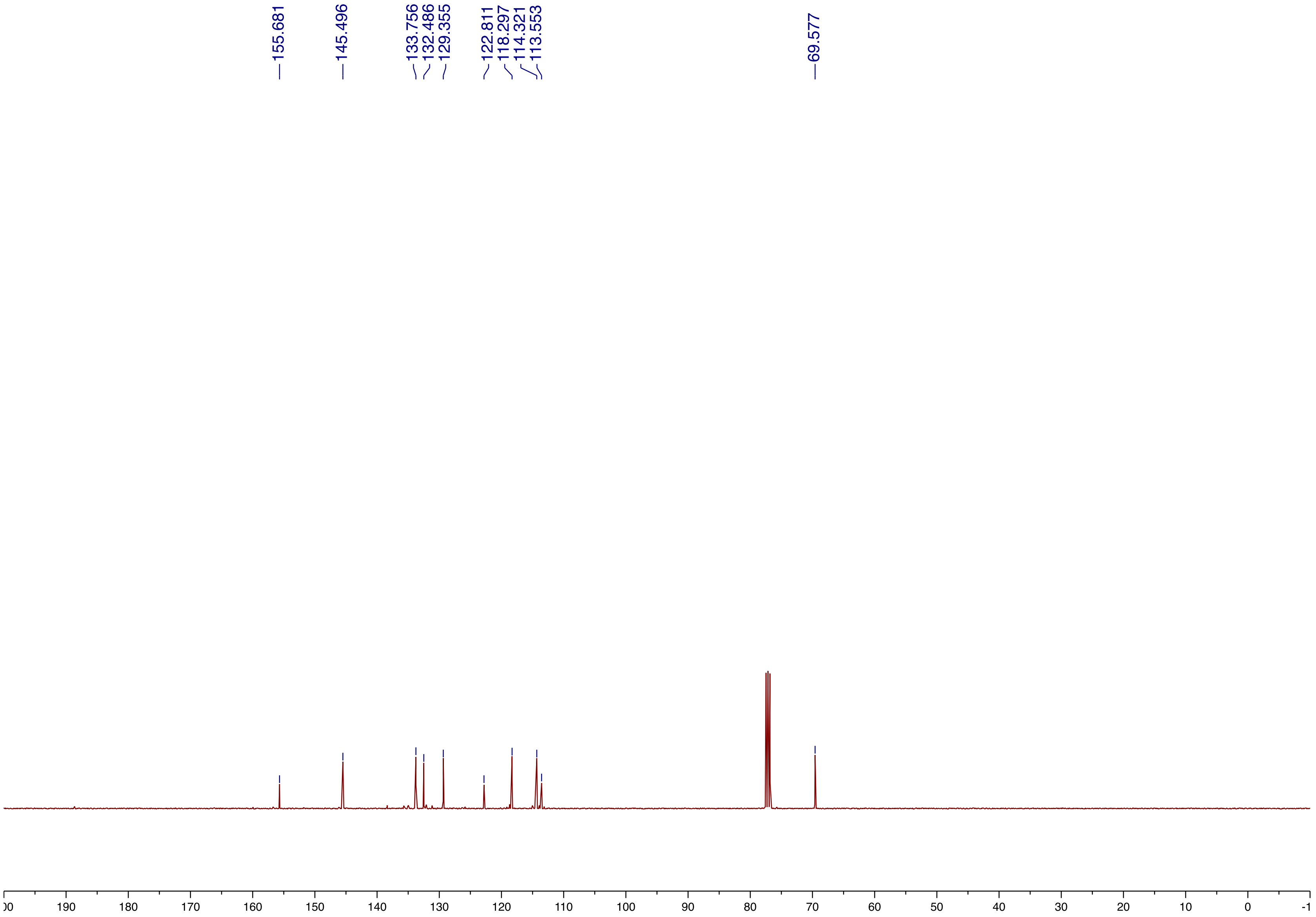

^{1}H NMR (400 MHz, $CDCl_3$)

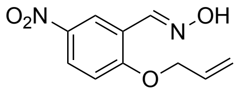

1o

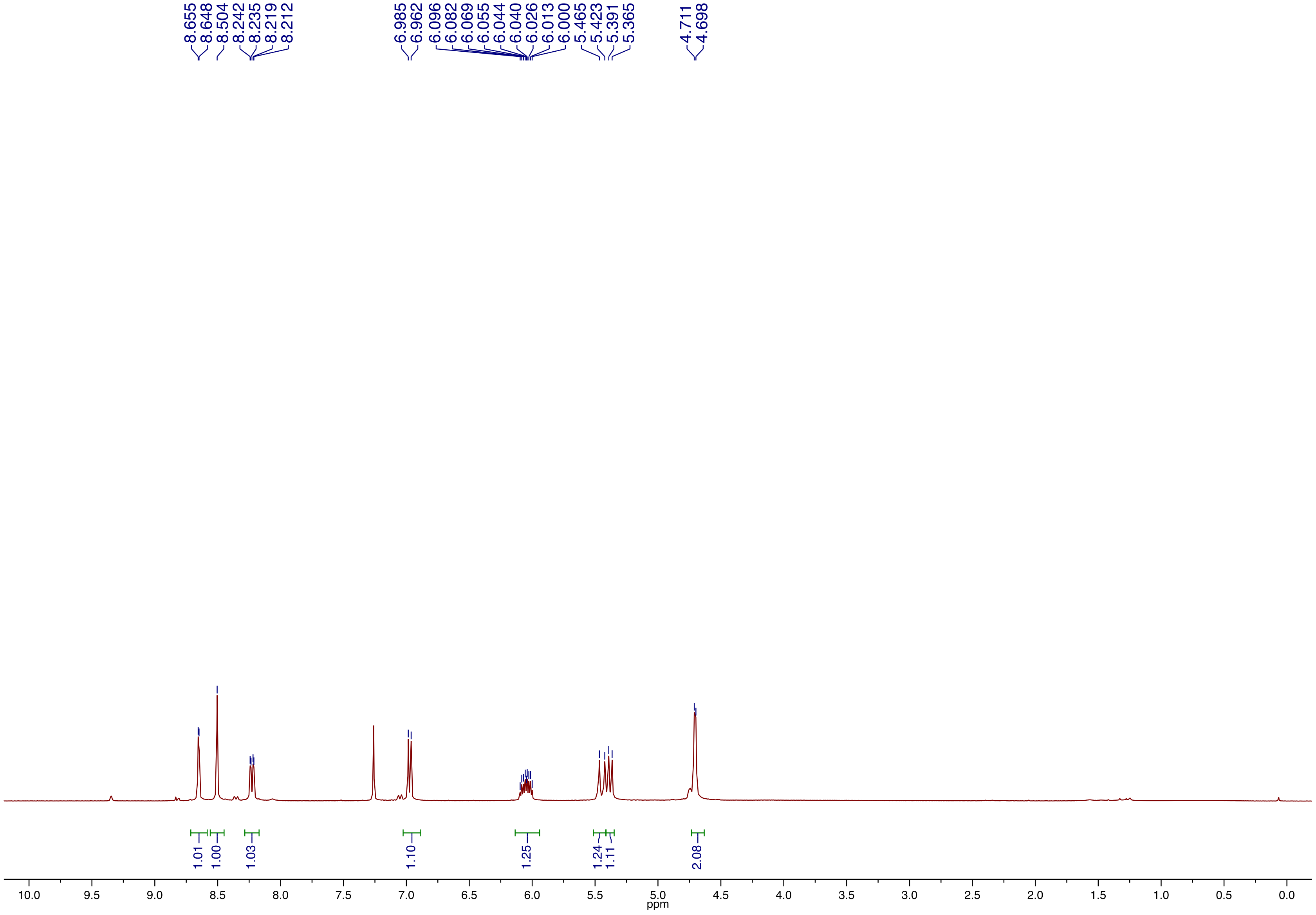

¹³C NMR (101 MHz, $CDCl_3$)

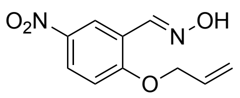

1o

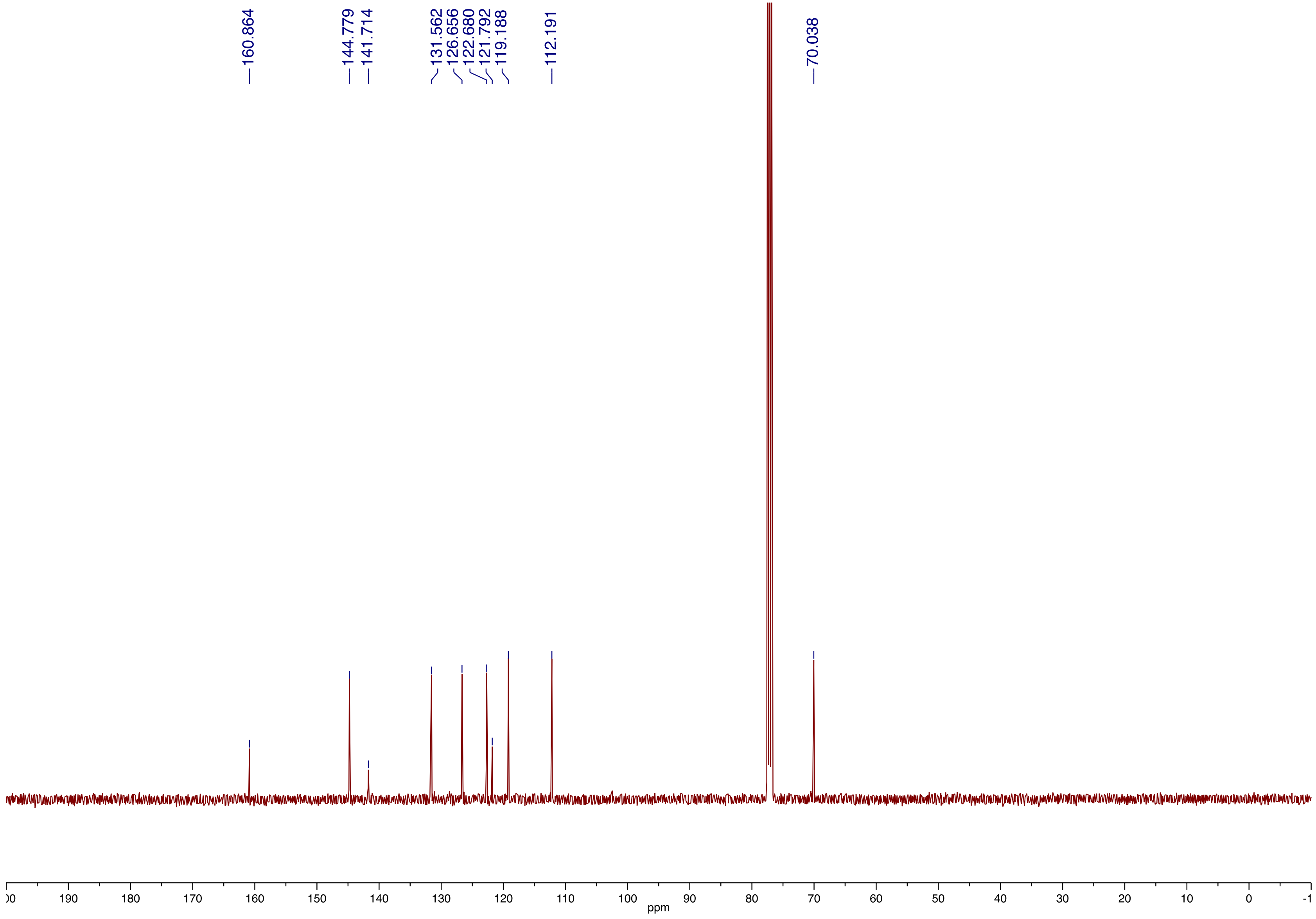

^{1}H NMR (400 MHz, $CDCl_3$)

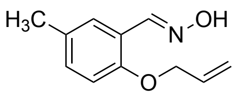

1p

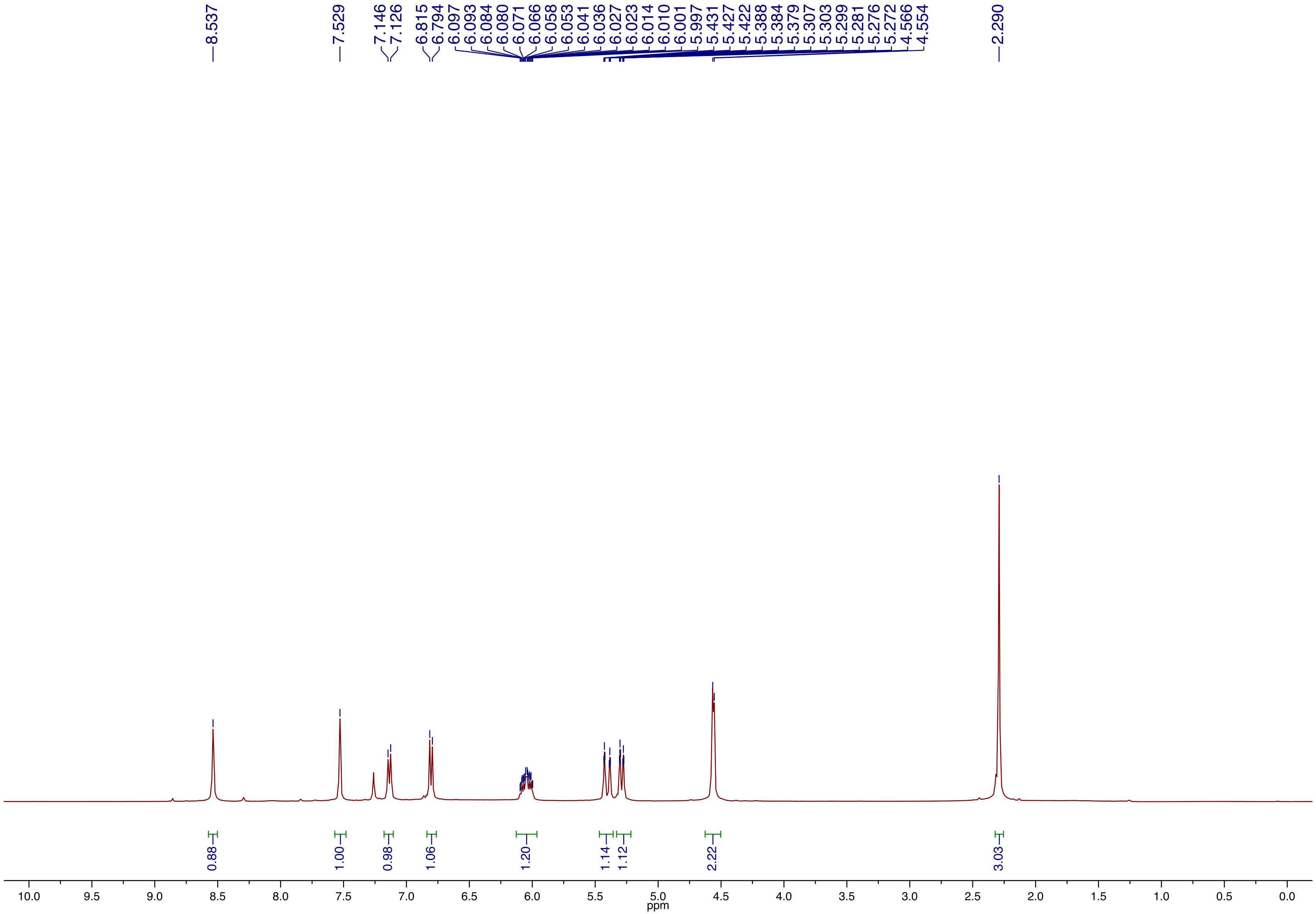

^{13}C NMR (101 MHz, $CDCl_3$)

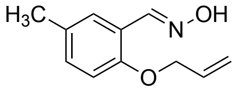

1p

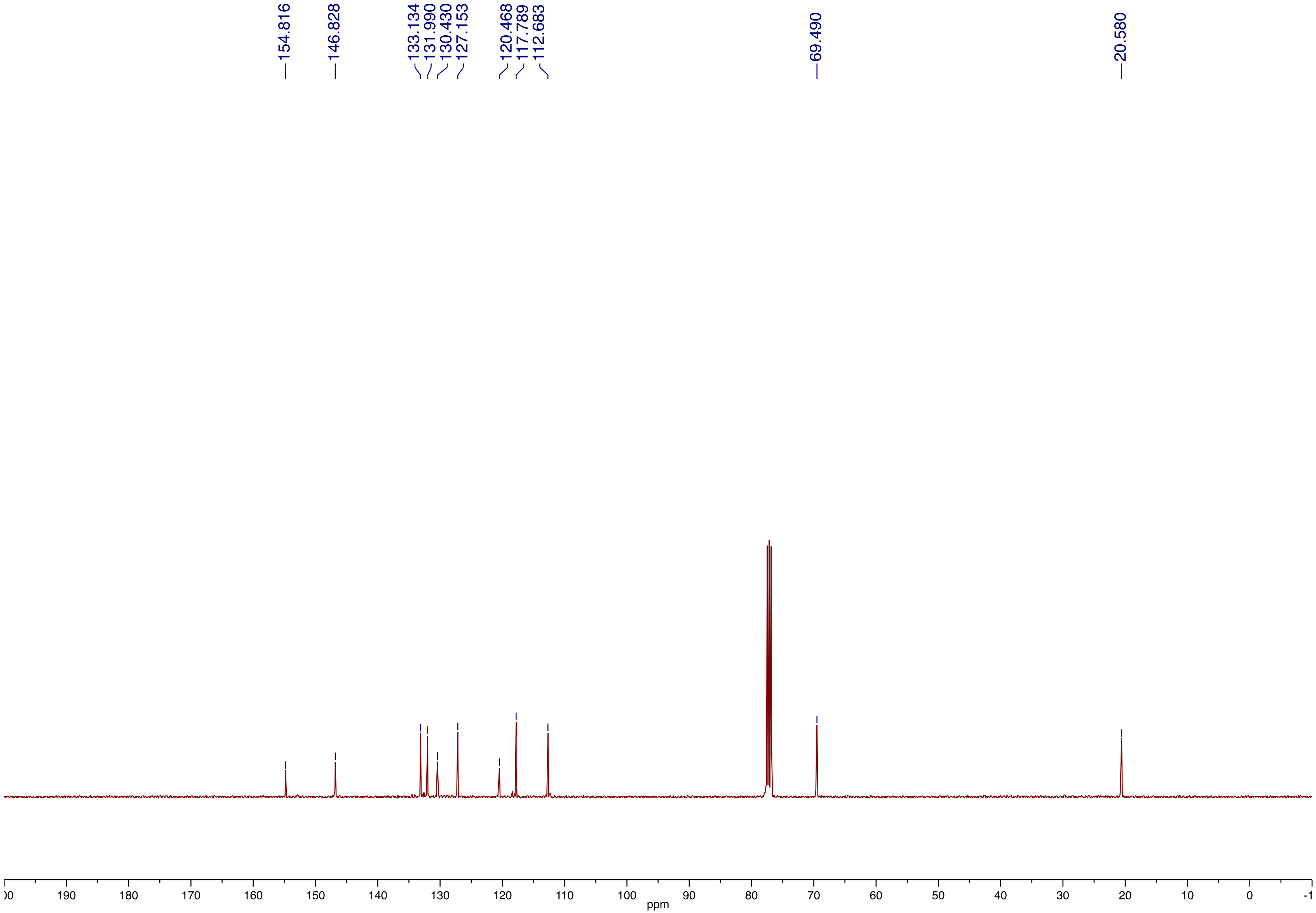

^1H NMR (400 MHz, $CDCl_3$)

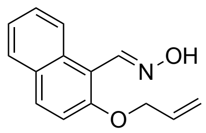

1q

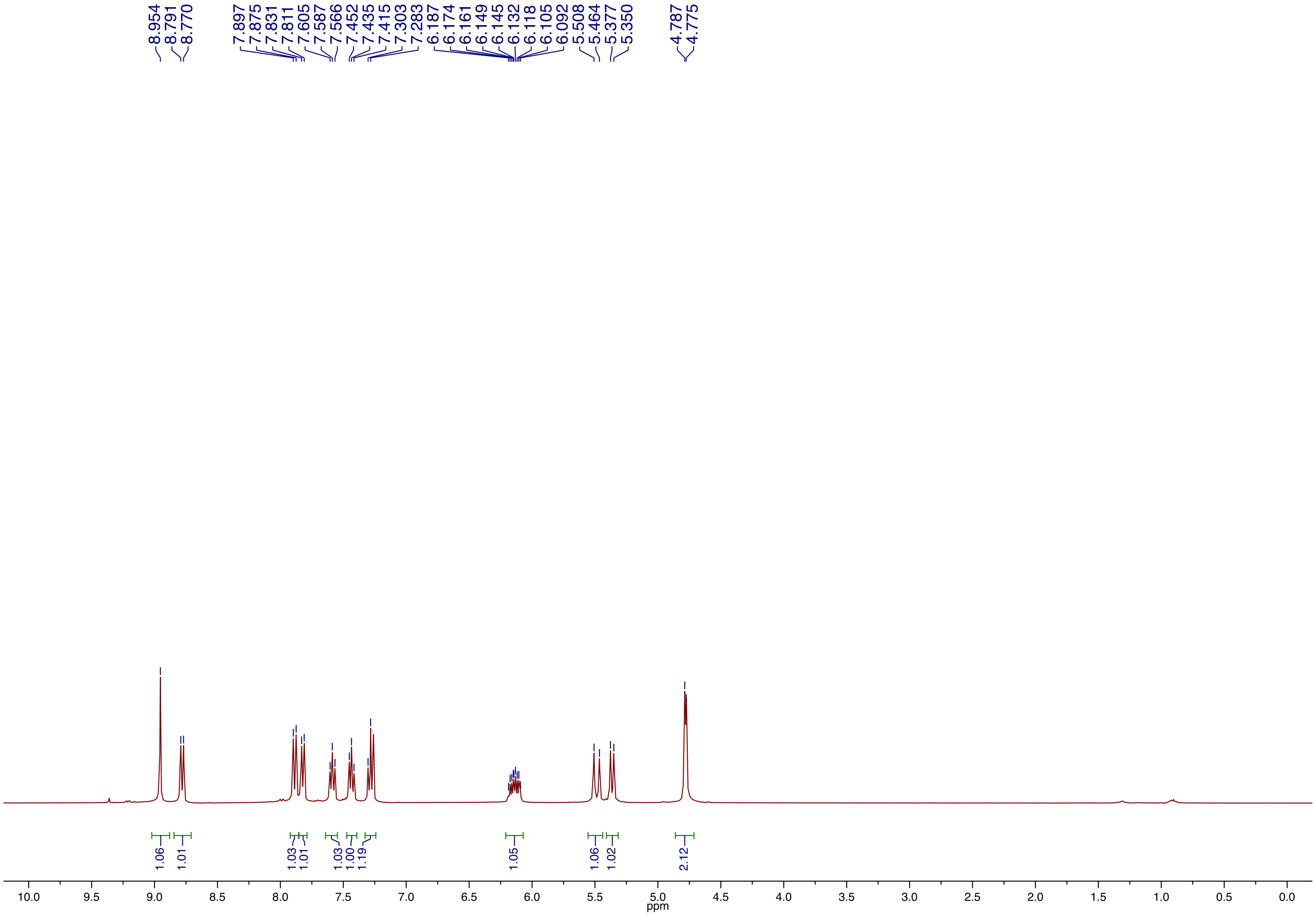

13C NMR (101 MHz, CDCl3)

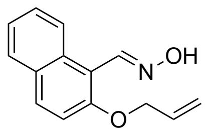

1q

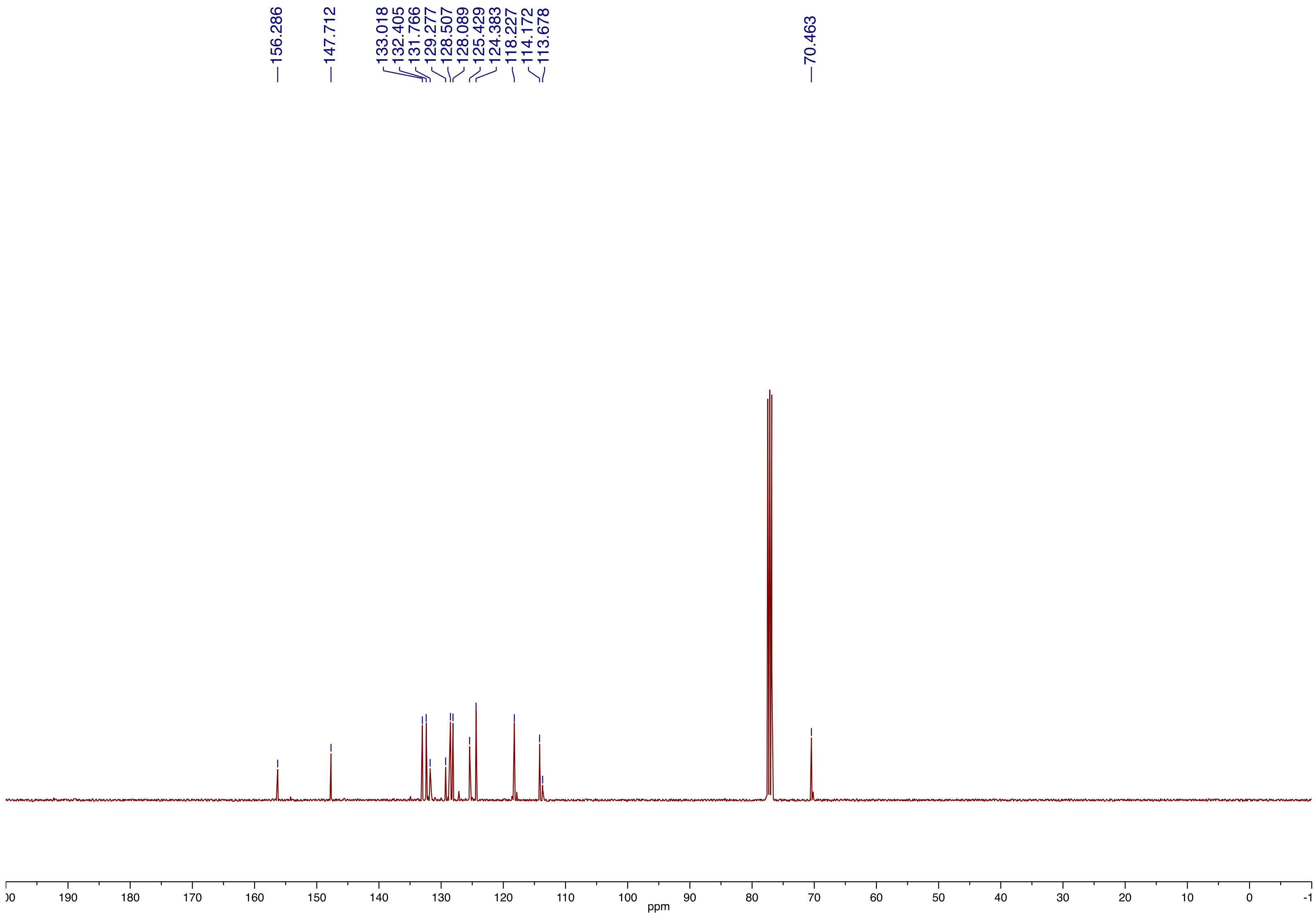

^1H NMR (400 MHz, $CDCl_3$)

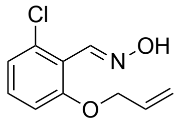

1r

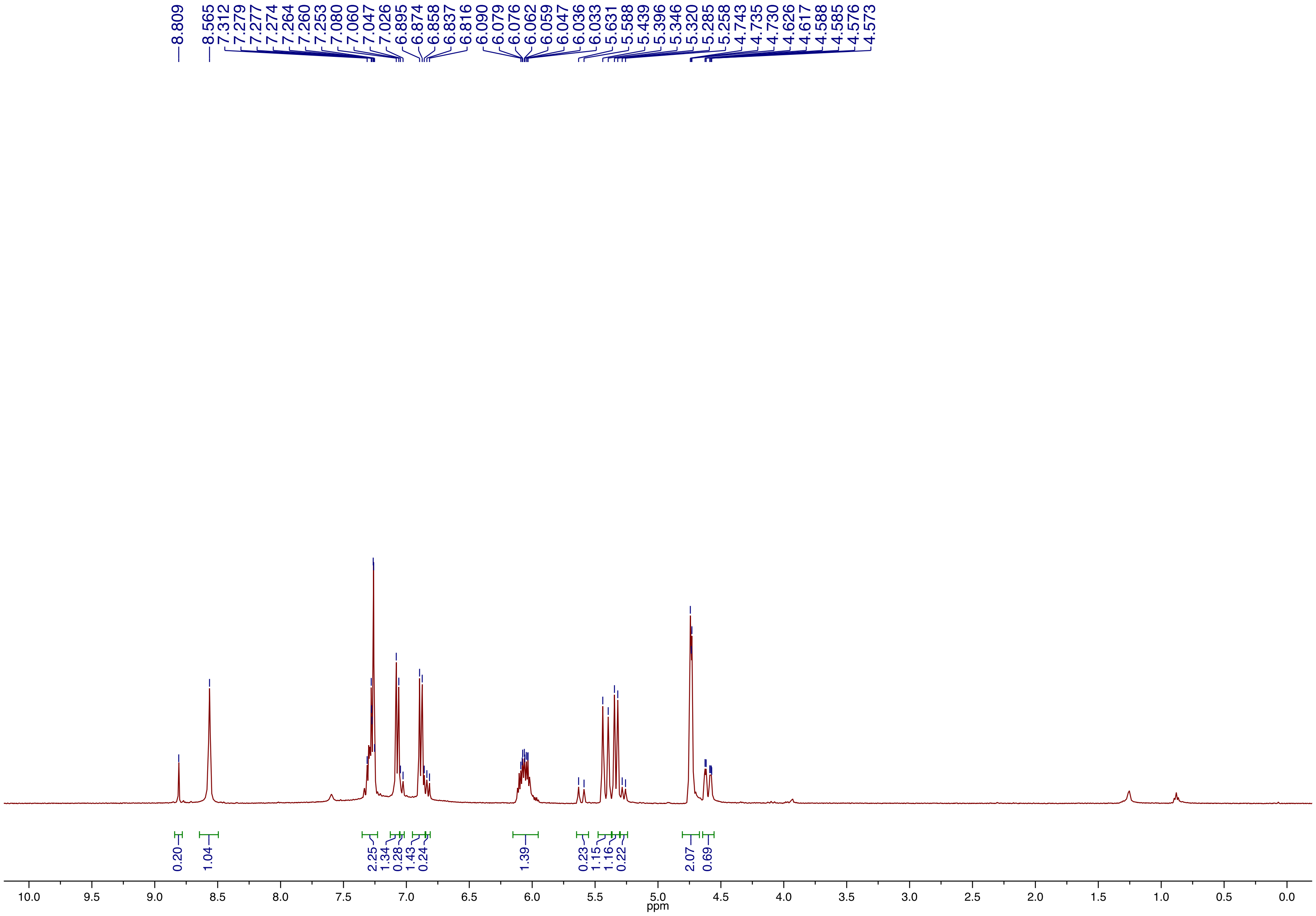

^{13}C NMR (101 MHz, $CDCl_3$)

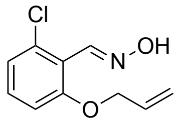

1r

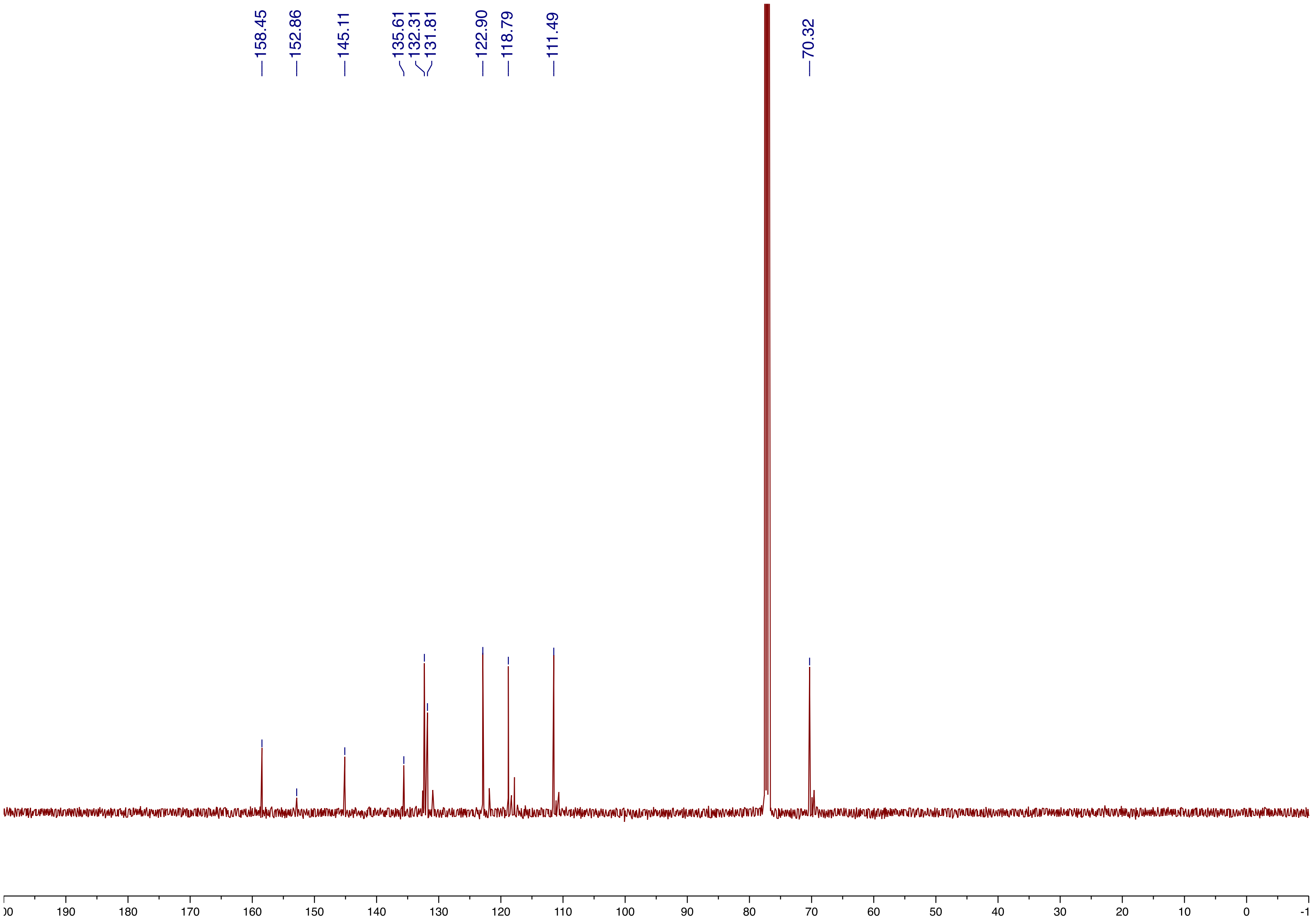

^{1}H NMR (400 MHz, $CDCl_3$)

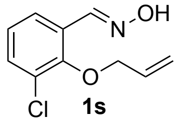

1s

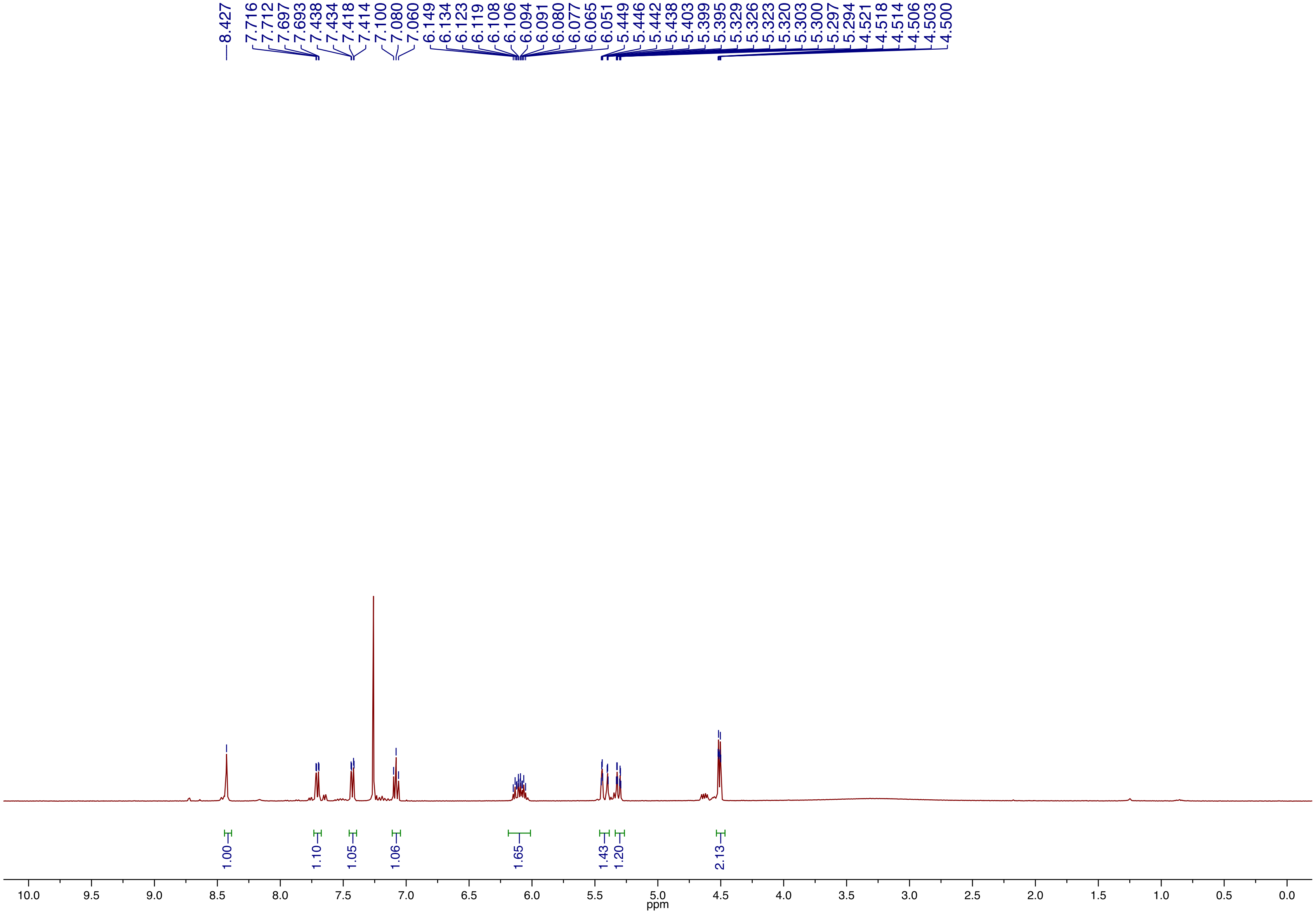

^{13}C NMR (101 MHz, $CDCl_3$)

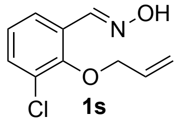

1s

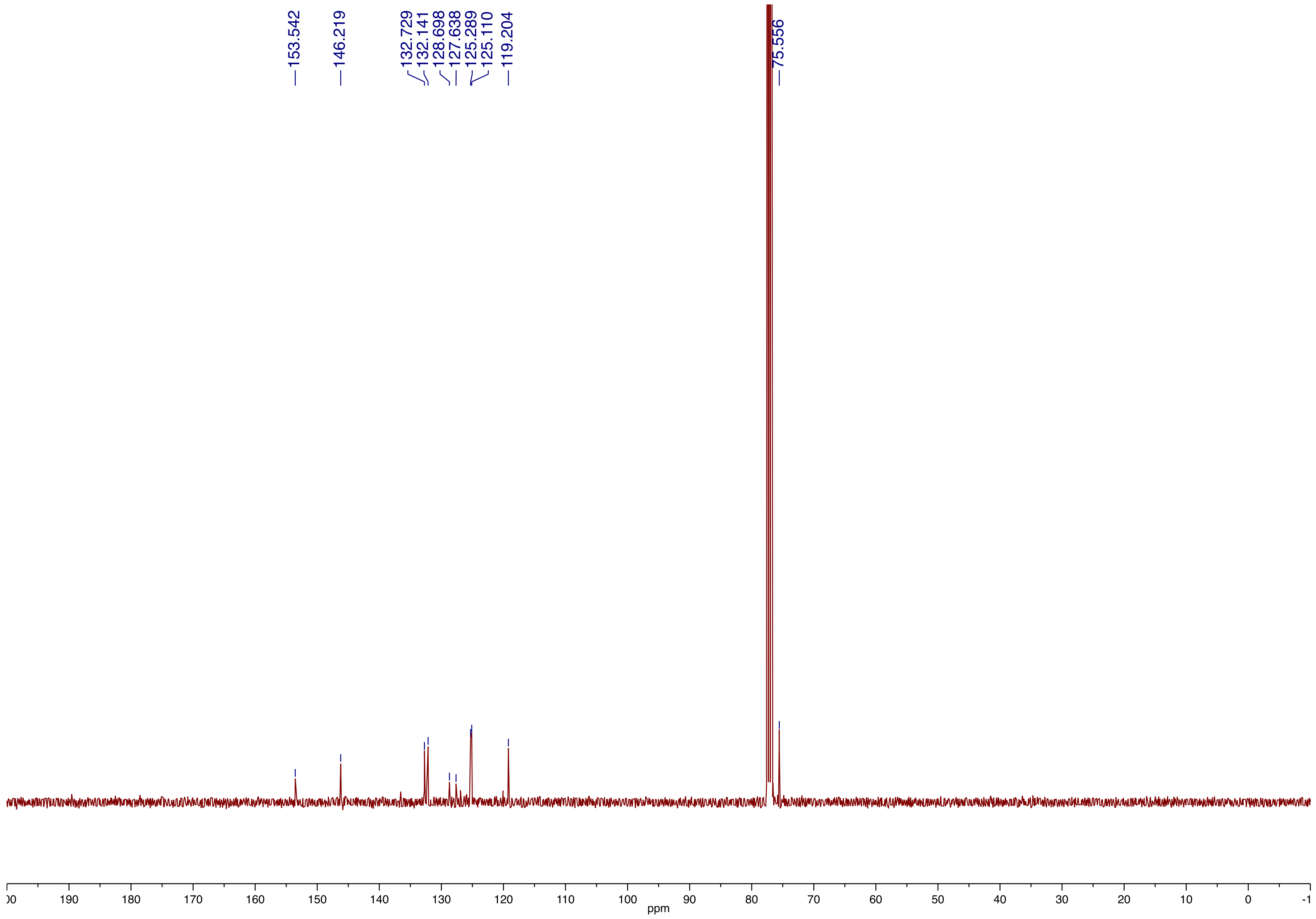

^{1}H NMR (400 MHz, $CDCl_3$)

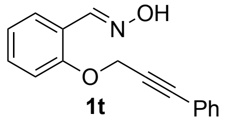

1t

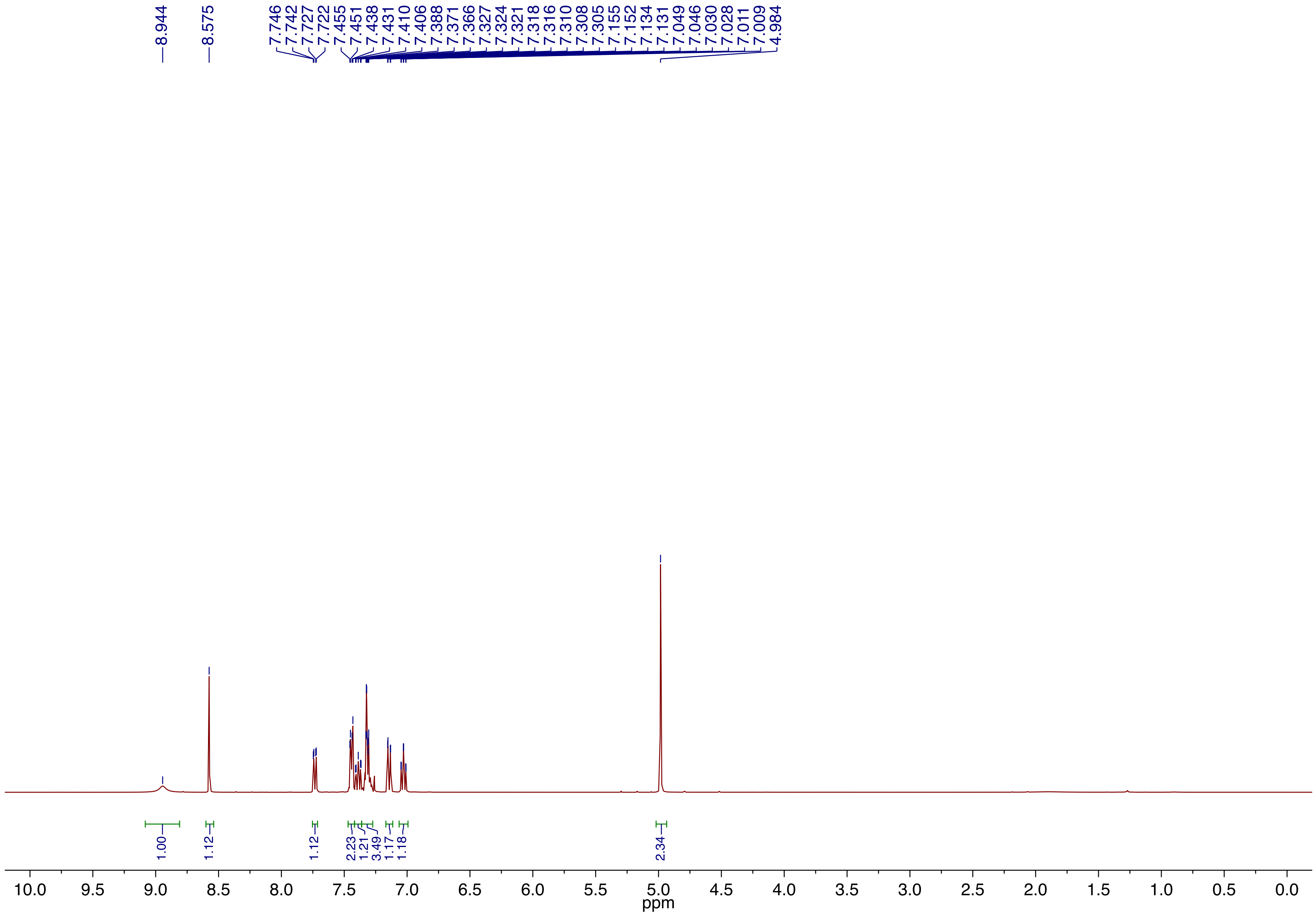

^{13}C NMR (101 MHz, $CDCl_3$)

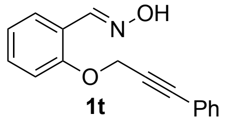

1t

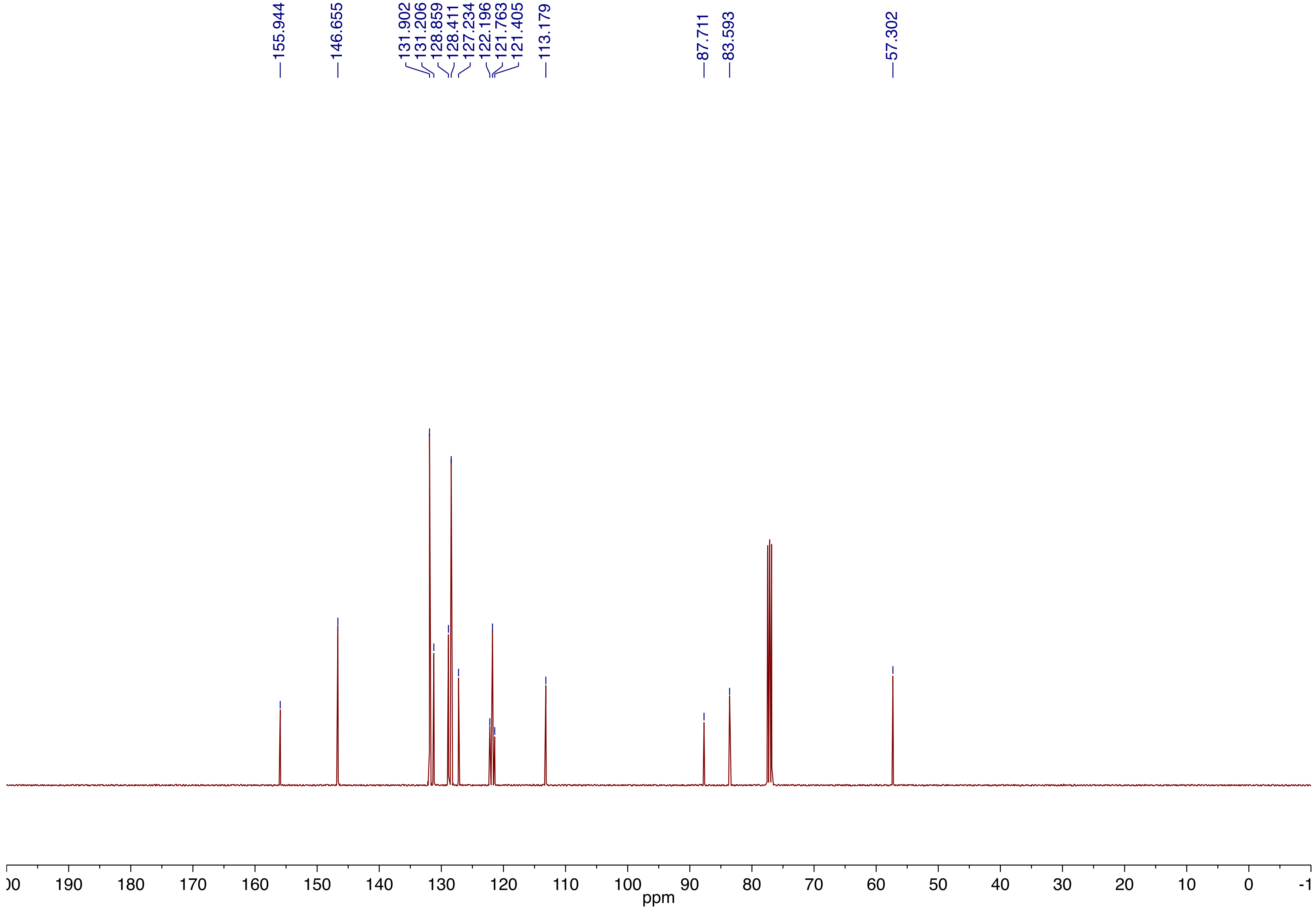

¹H NMR (400 MHz, DMSO-d6)

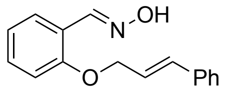

1u

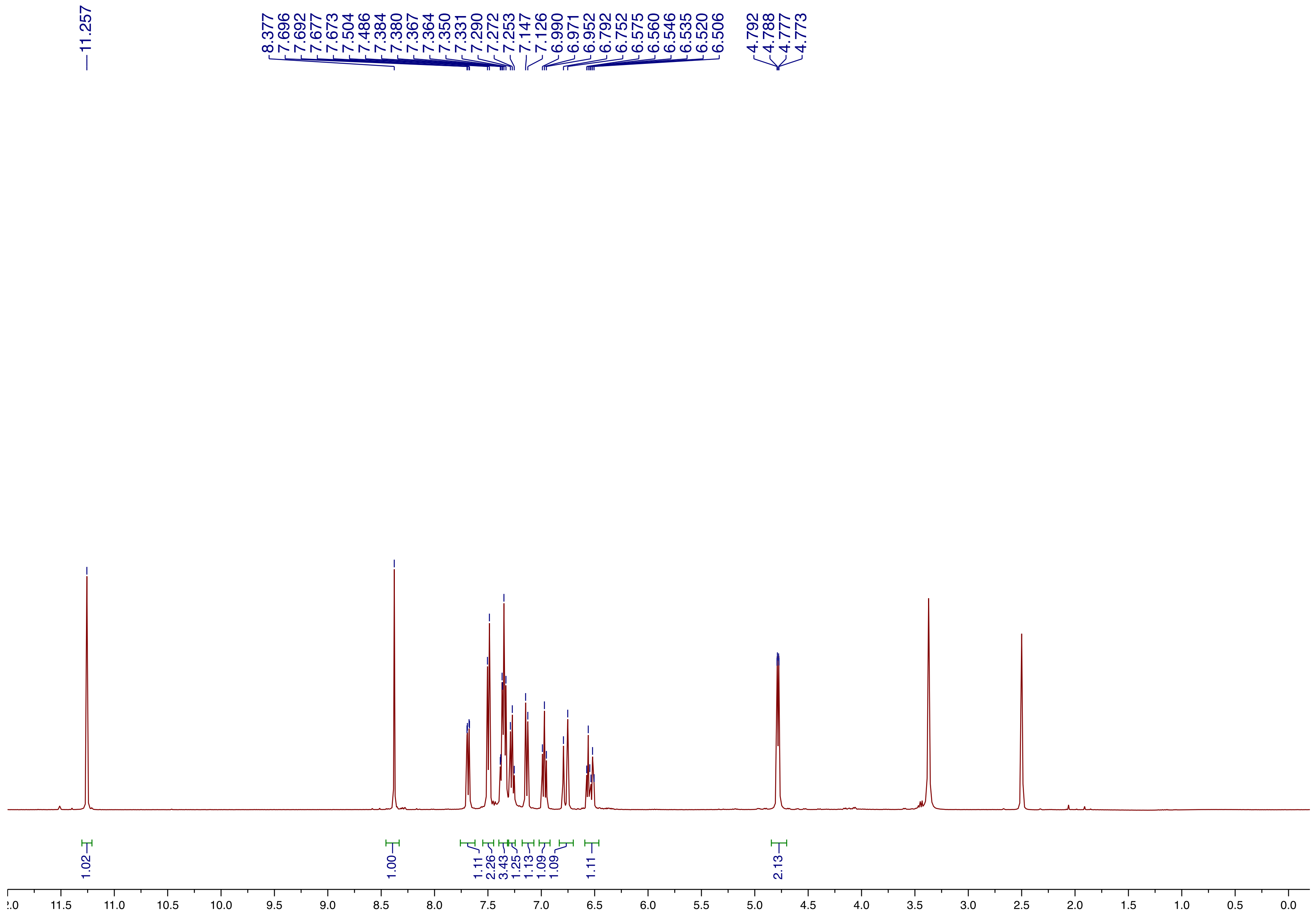

^{13}C NMR (101 MHz, DMSO-d_6)

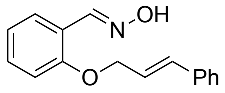

1u

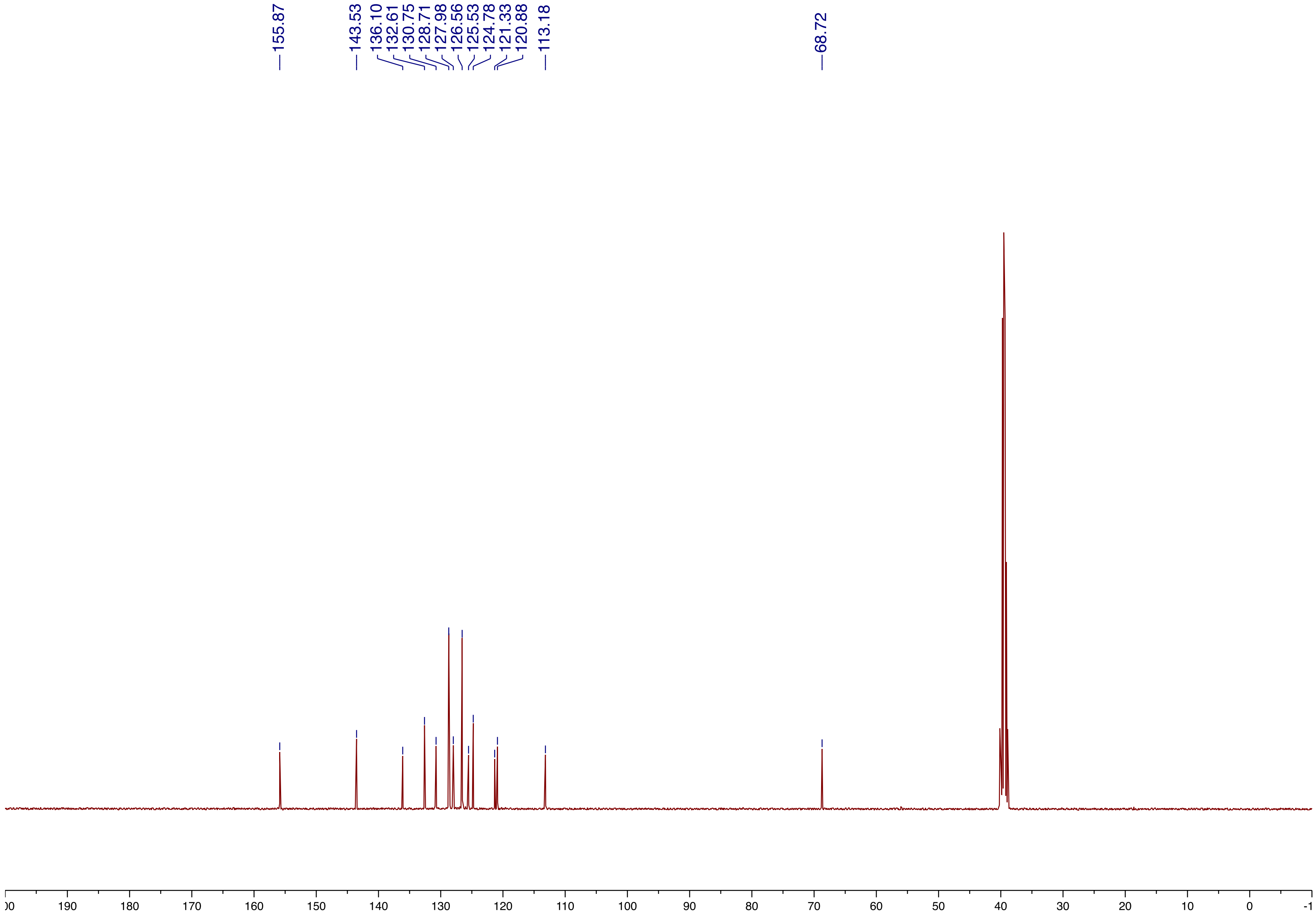

^{1}H NMR (400 MHz, $CDCl_3$)

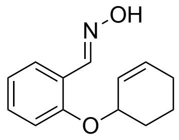

1v

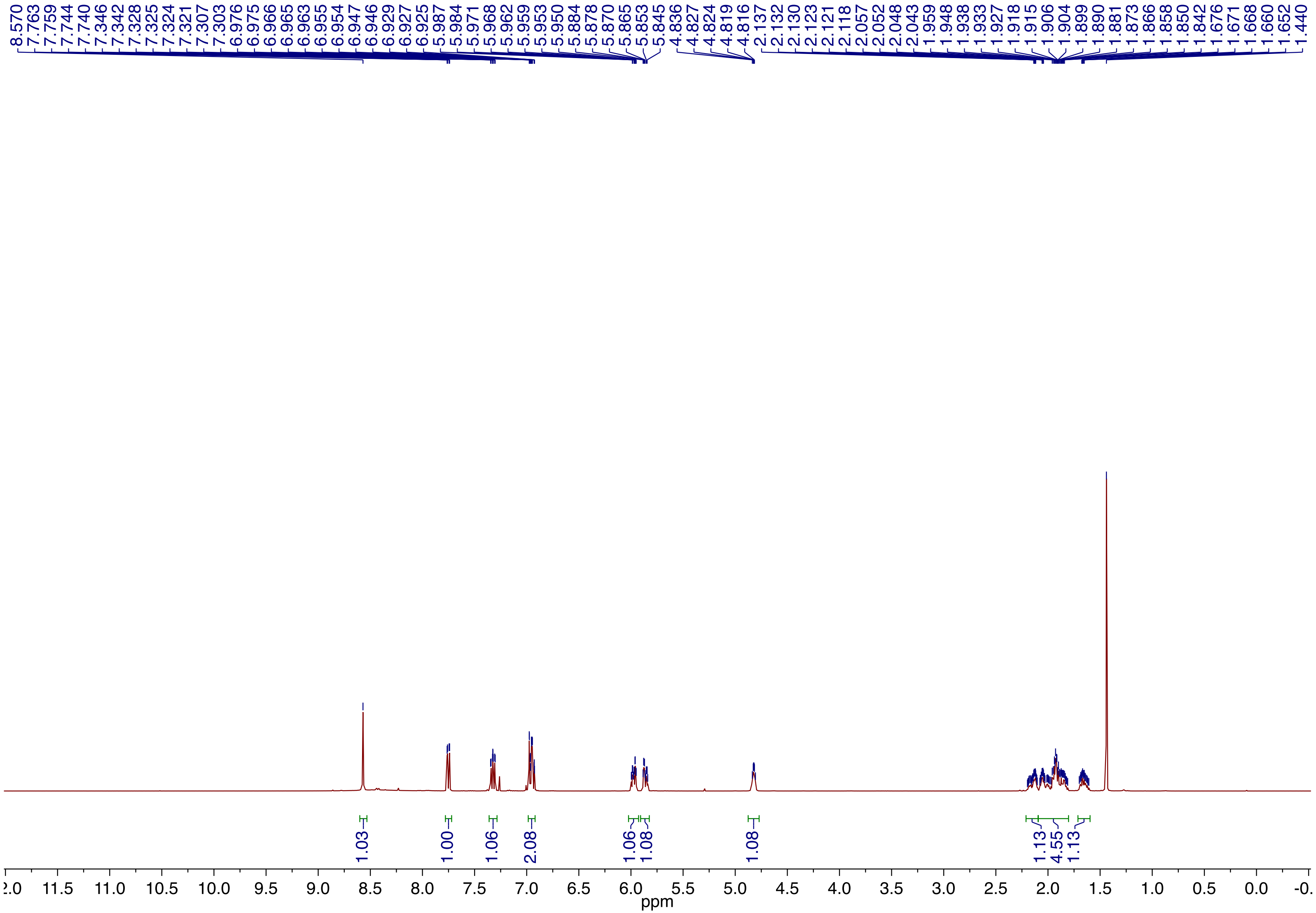

^{13}C NMR (101 MHz, $CDCl_3$)

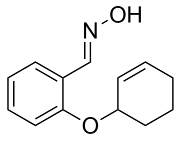

1v

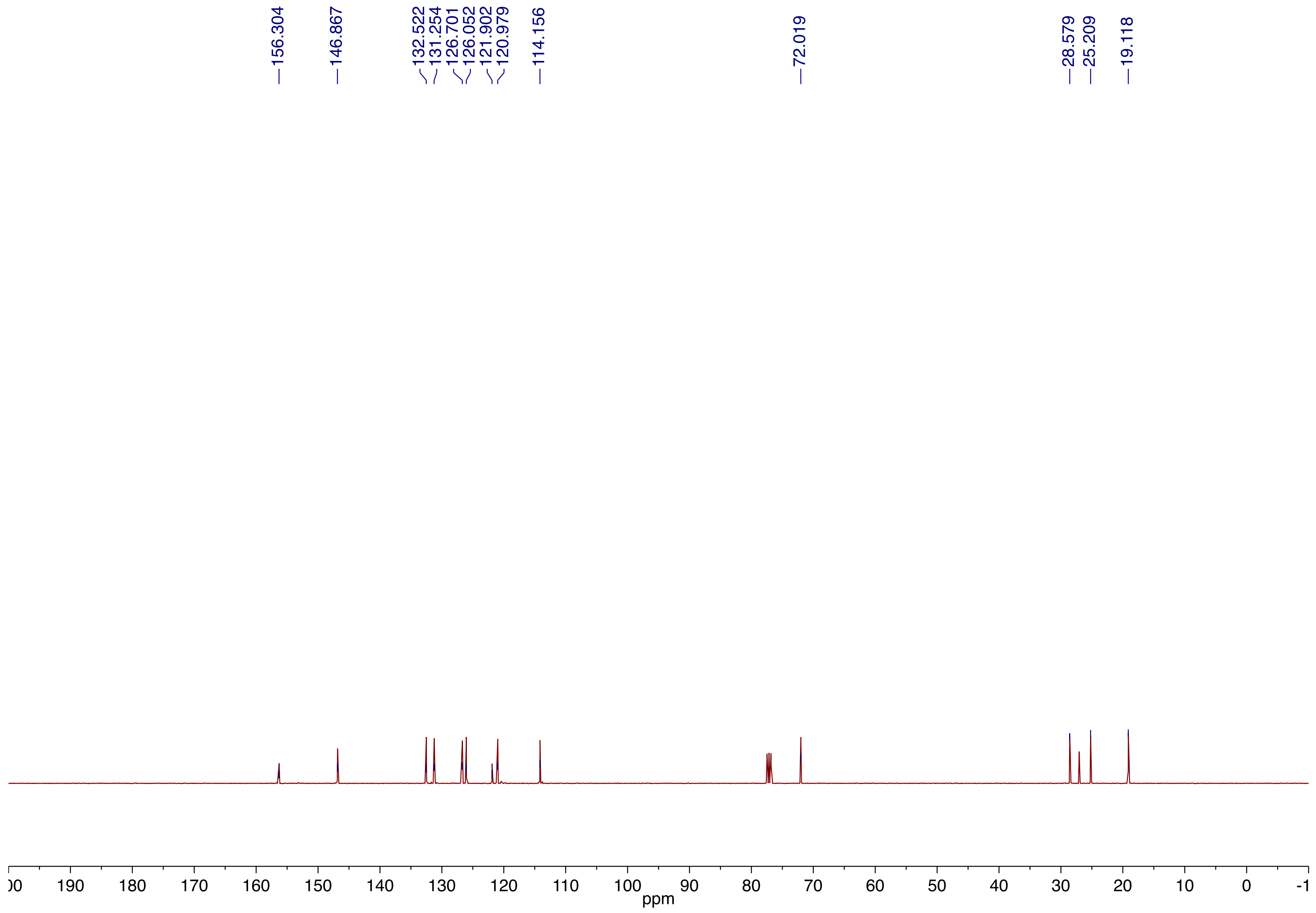

¹H NMR (400 MHz, $CDCl_3$)

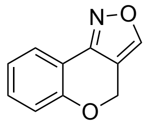

3a

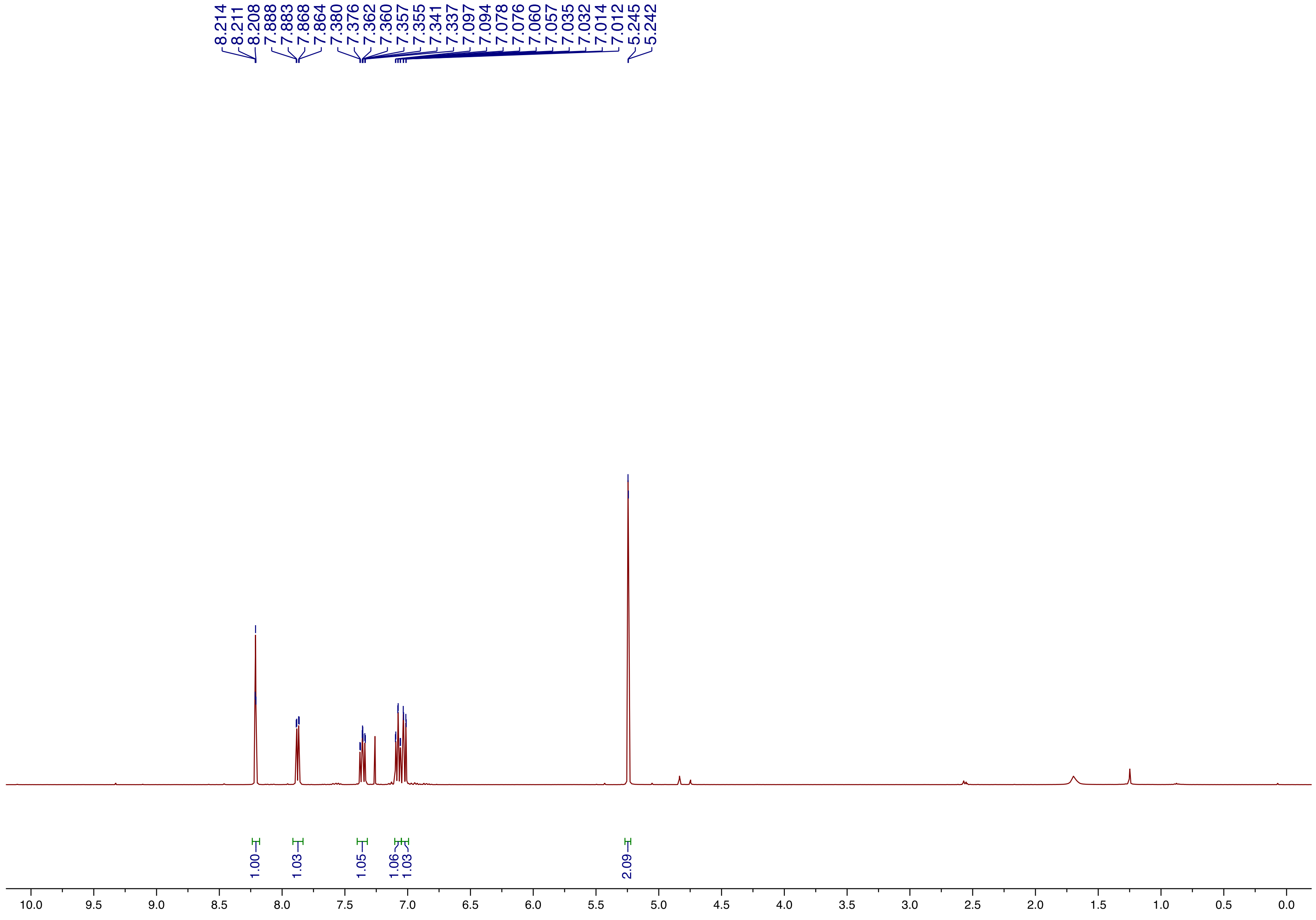

^{13}C NMR (101 MHz, $CDCl_3$)

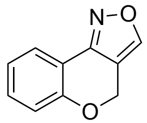

3a

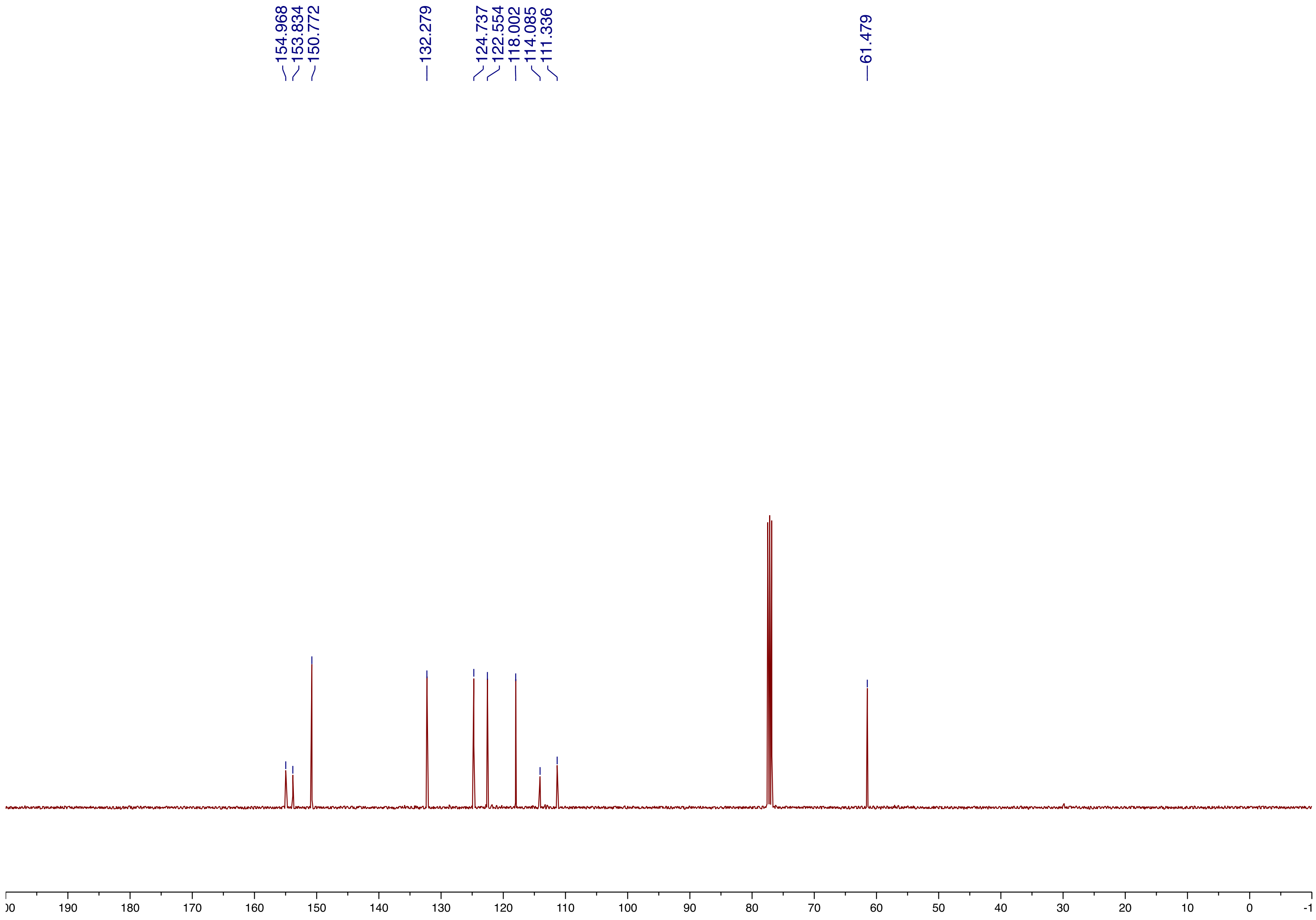

^{1}H NMR (400 MHz, $CDCl_3$)

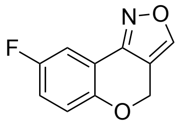

3b

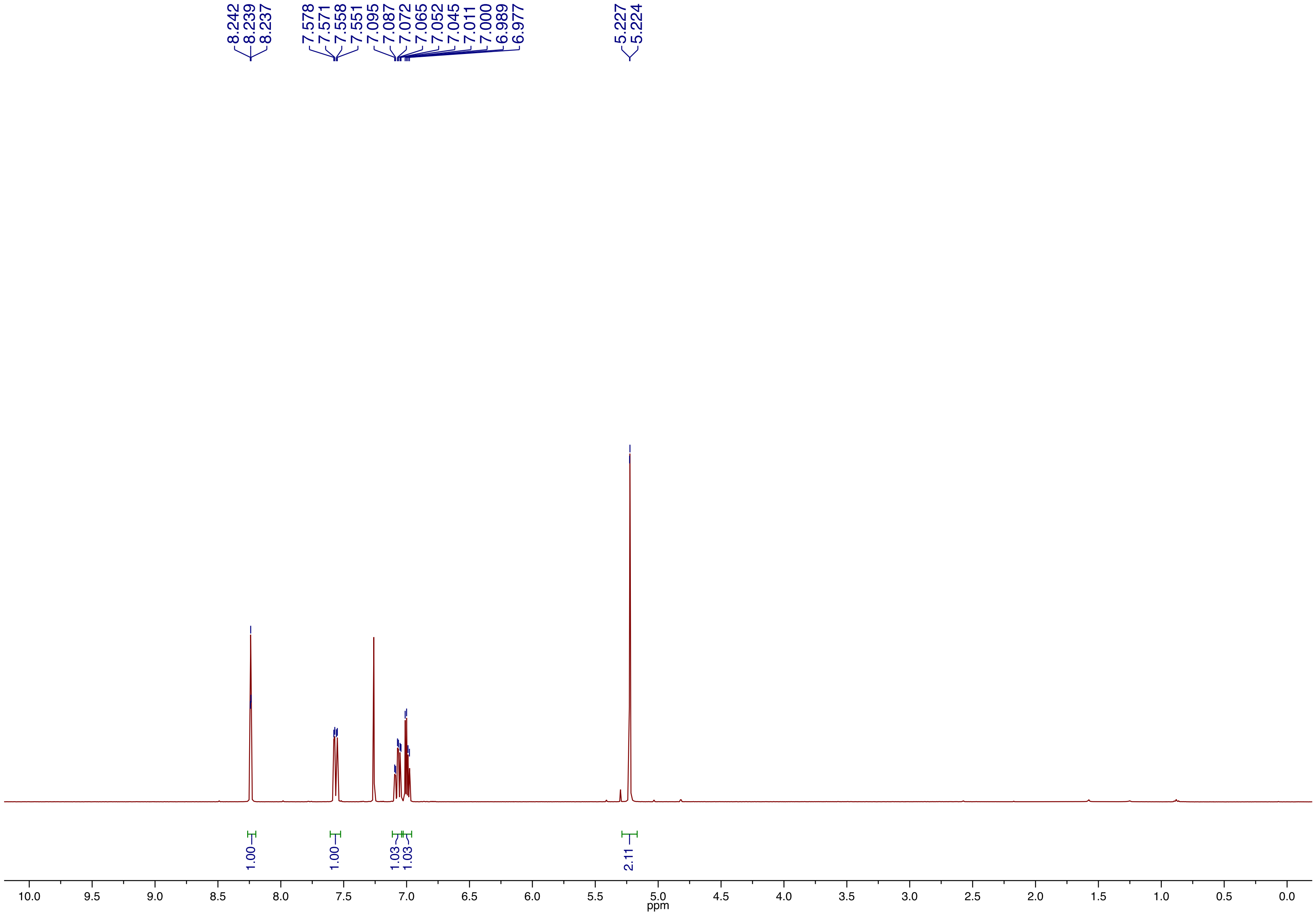

^{13}C NMR (101 MHz, $CDCl_3$)

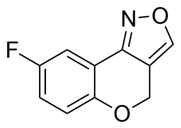

3b

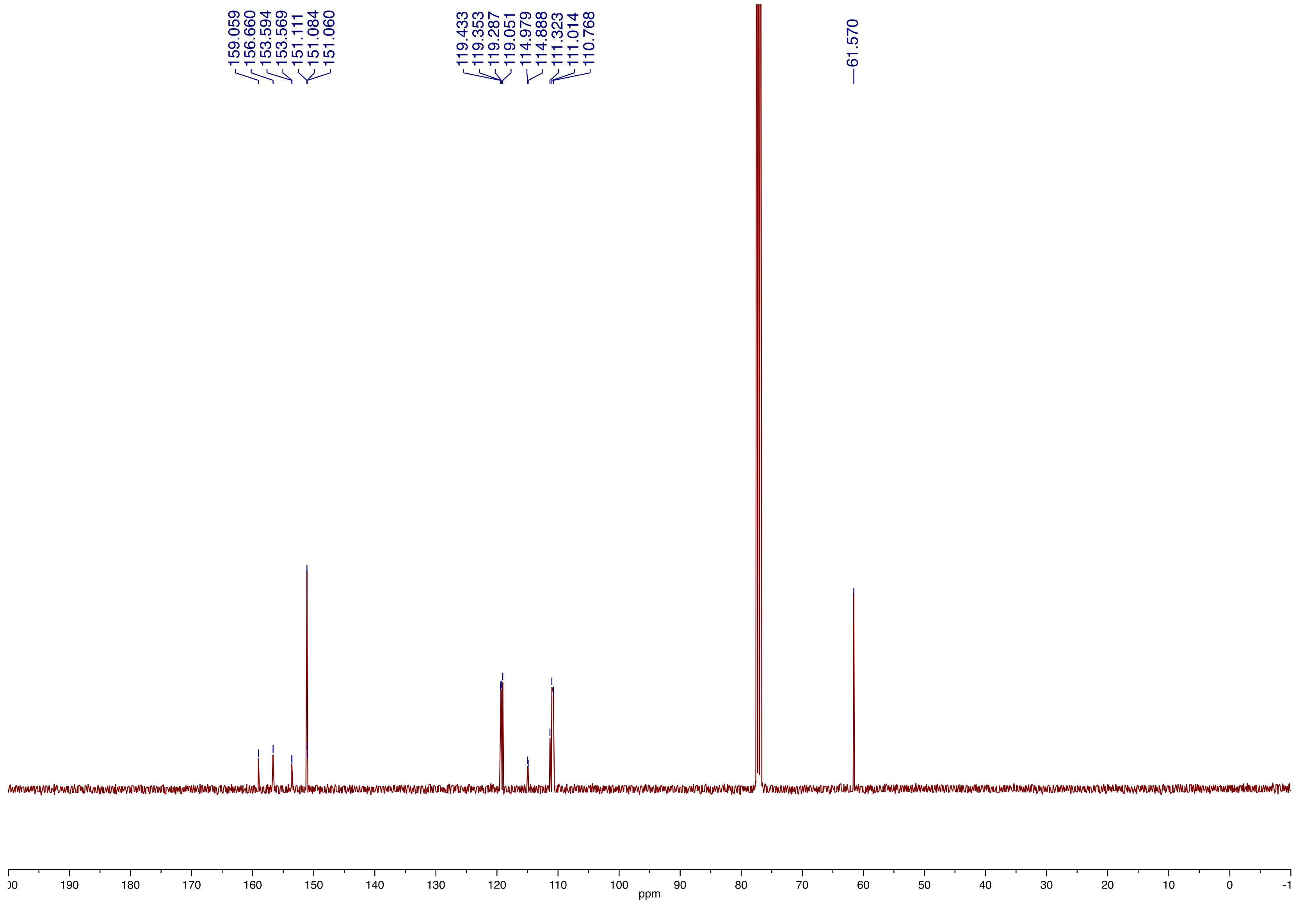

¹⁹F NMR (376 MHz, $CDCl_3$)

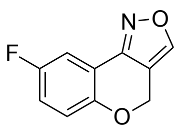

3b

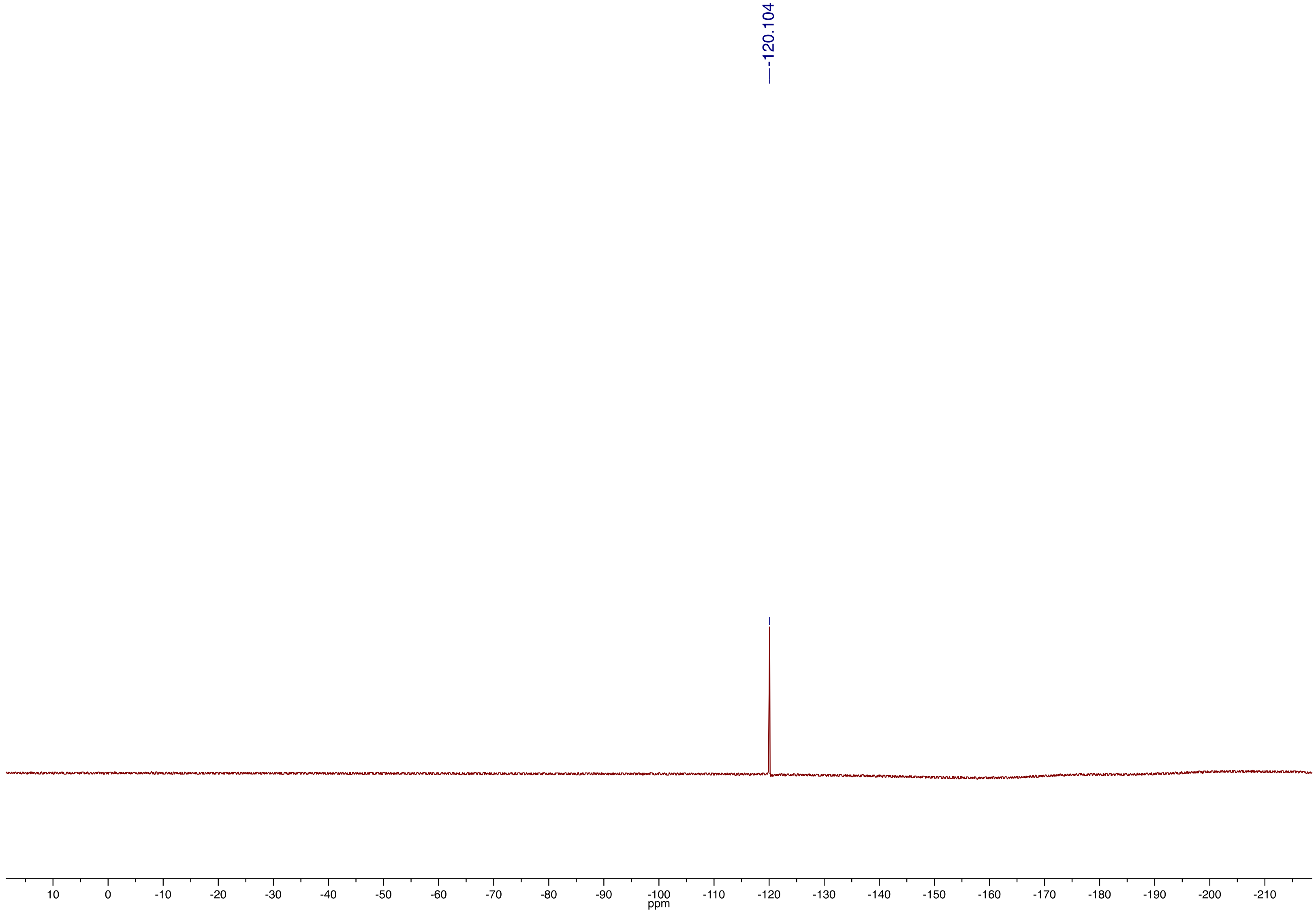

^{1}H NMR (400 MHz, $CDCl_3$)

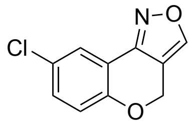

3c

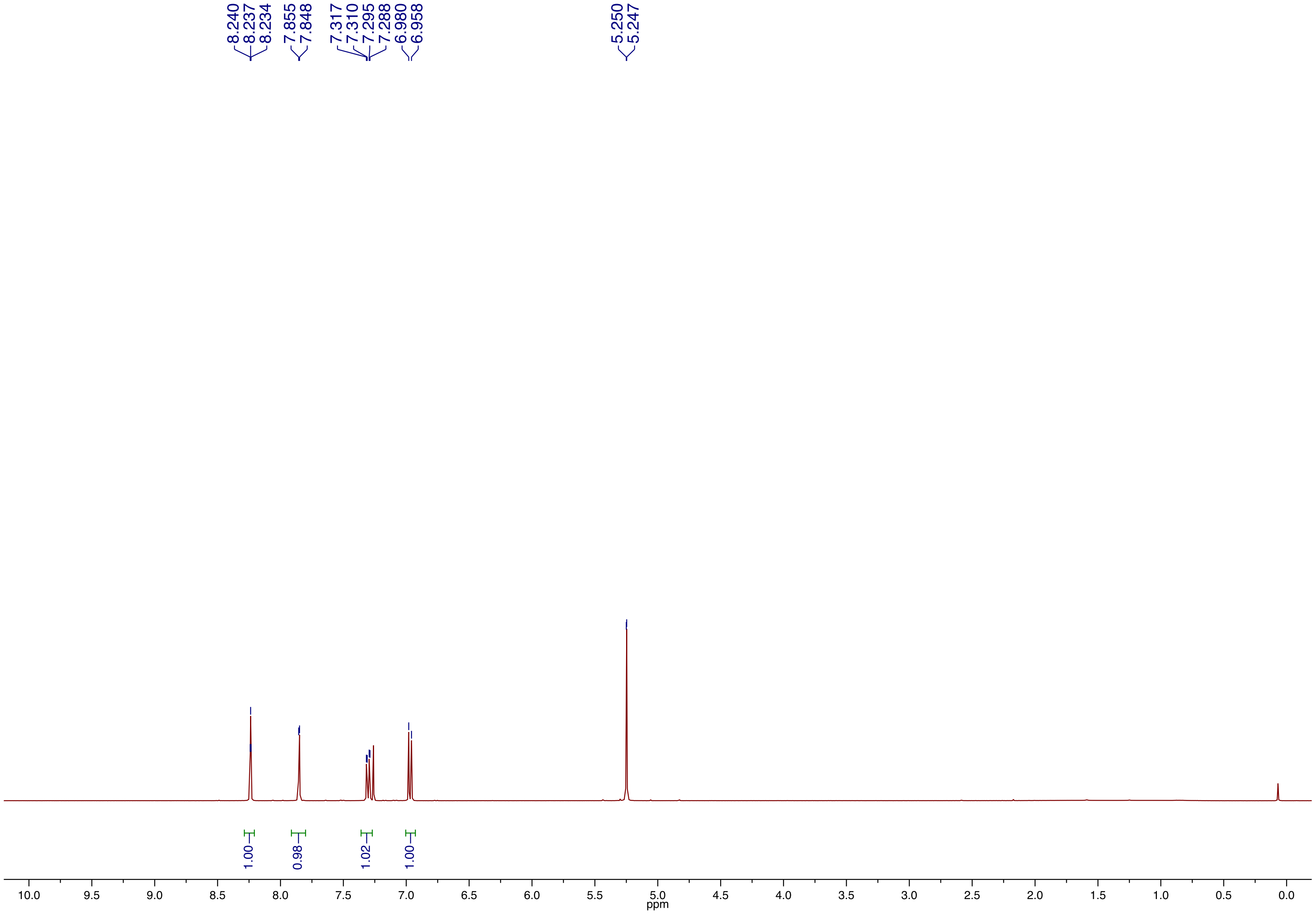

^{13}C NMR (101 MHz, $CDCl_3$)

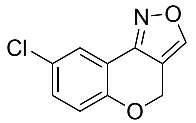

3c

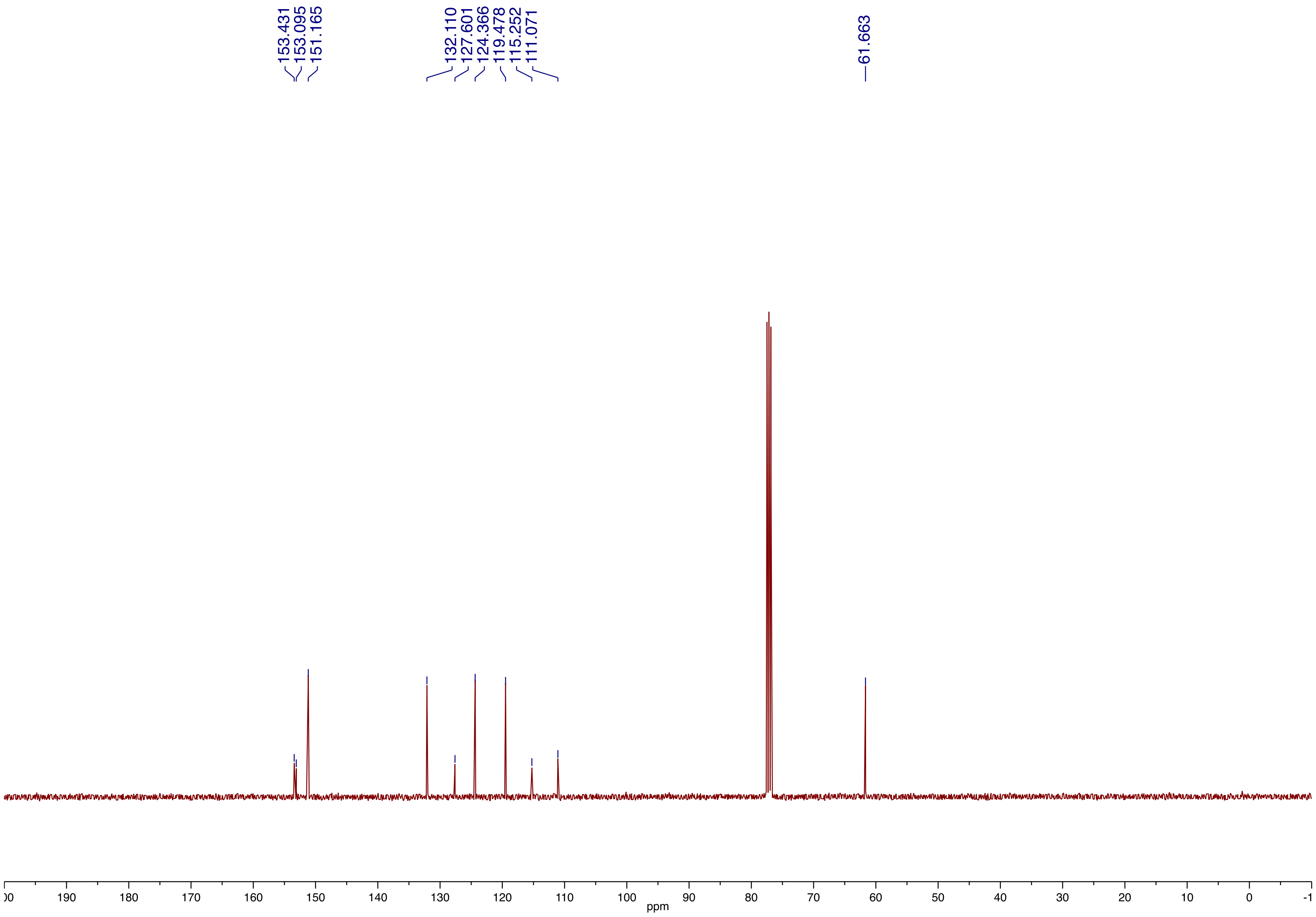

^1H NMR (400 MHz, $CDCl_3$)

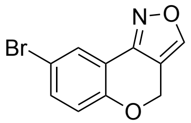

3d

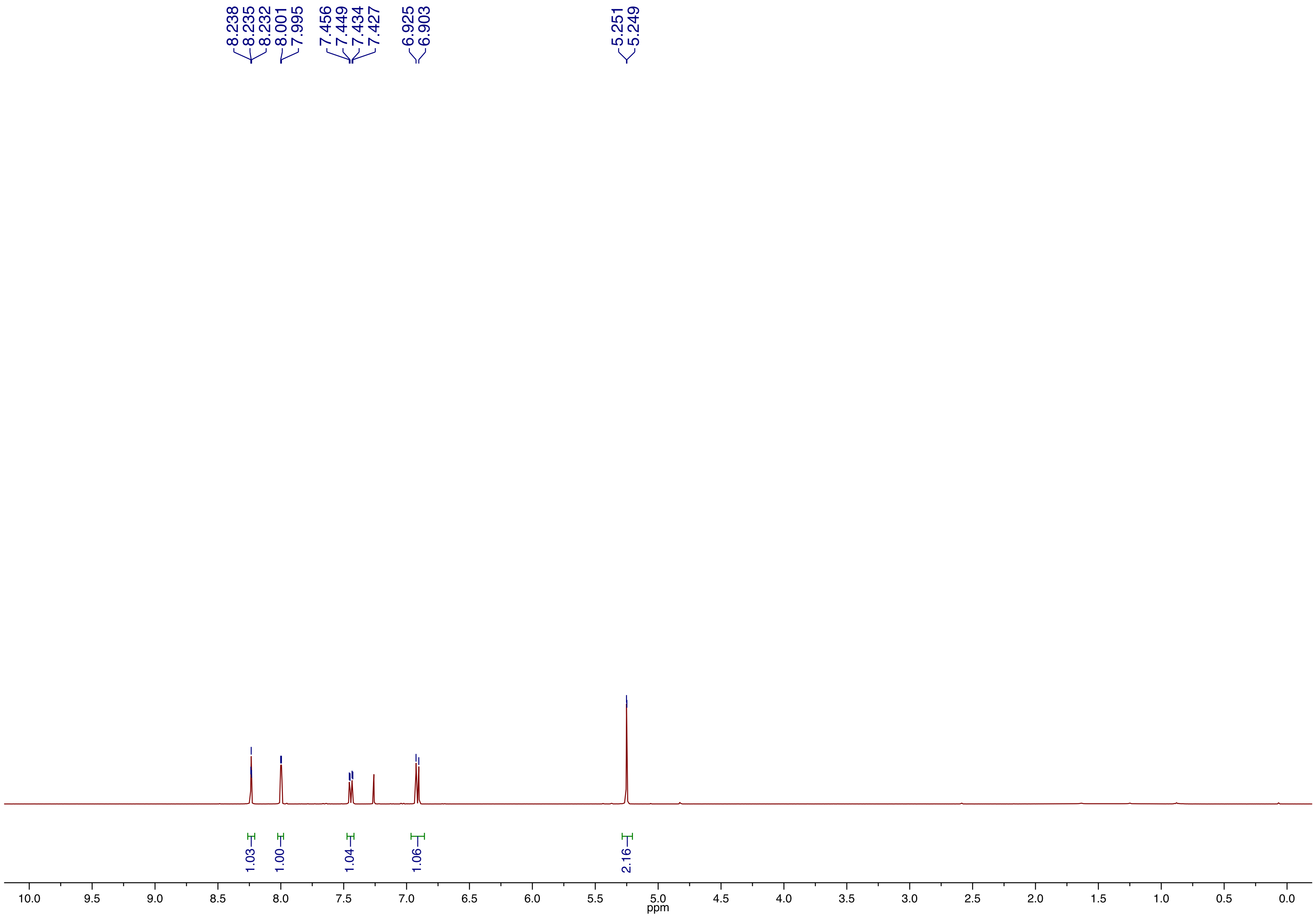

^{13}C NMR (101 MHz, $CDCl_3$)

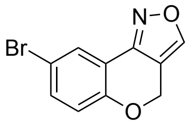

3d

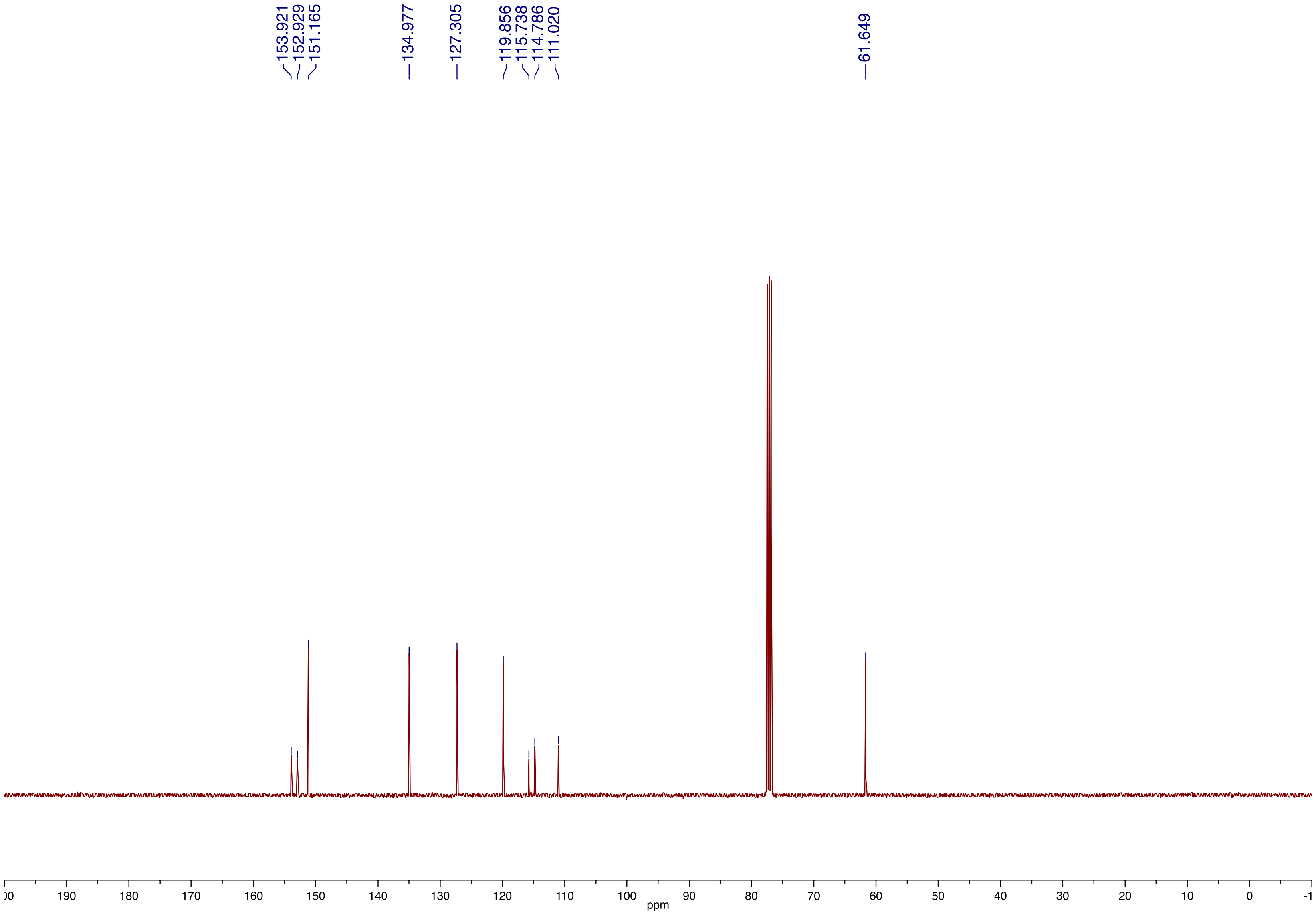

^{1}H NMR (400 MHz, $CDCl_3$)

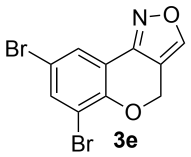

3e

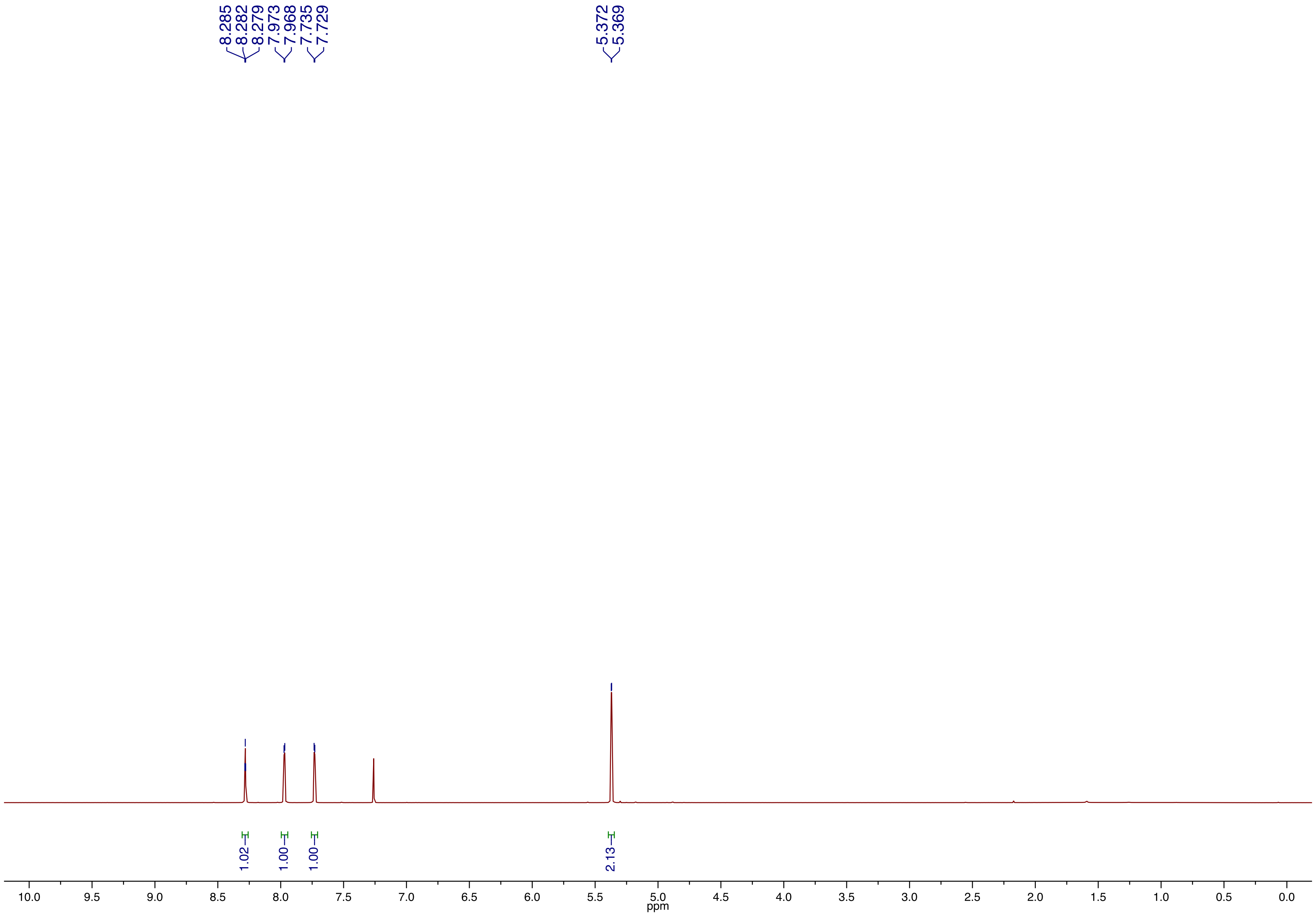

^{13}C NMR (101 MHz, CDCl$_3$)

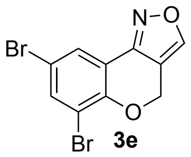

3e

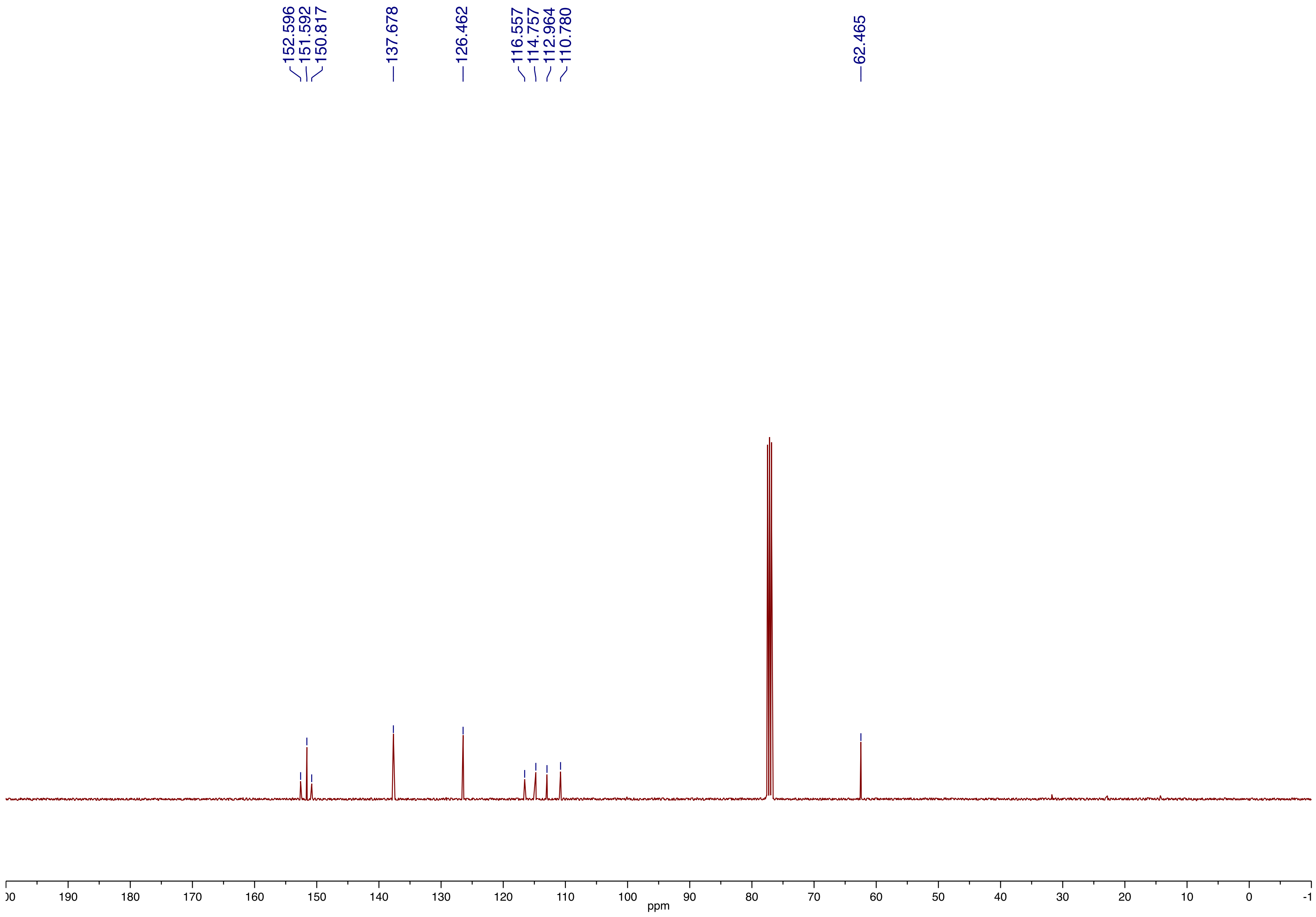

^{1}H NMR (400 MHz, $CDCl_3$)

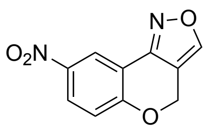

3f

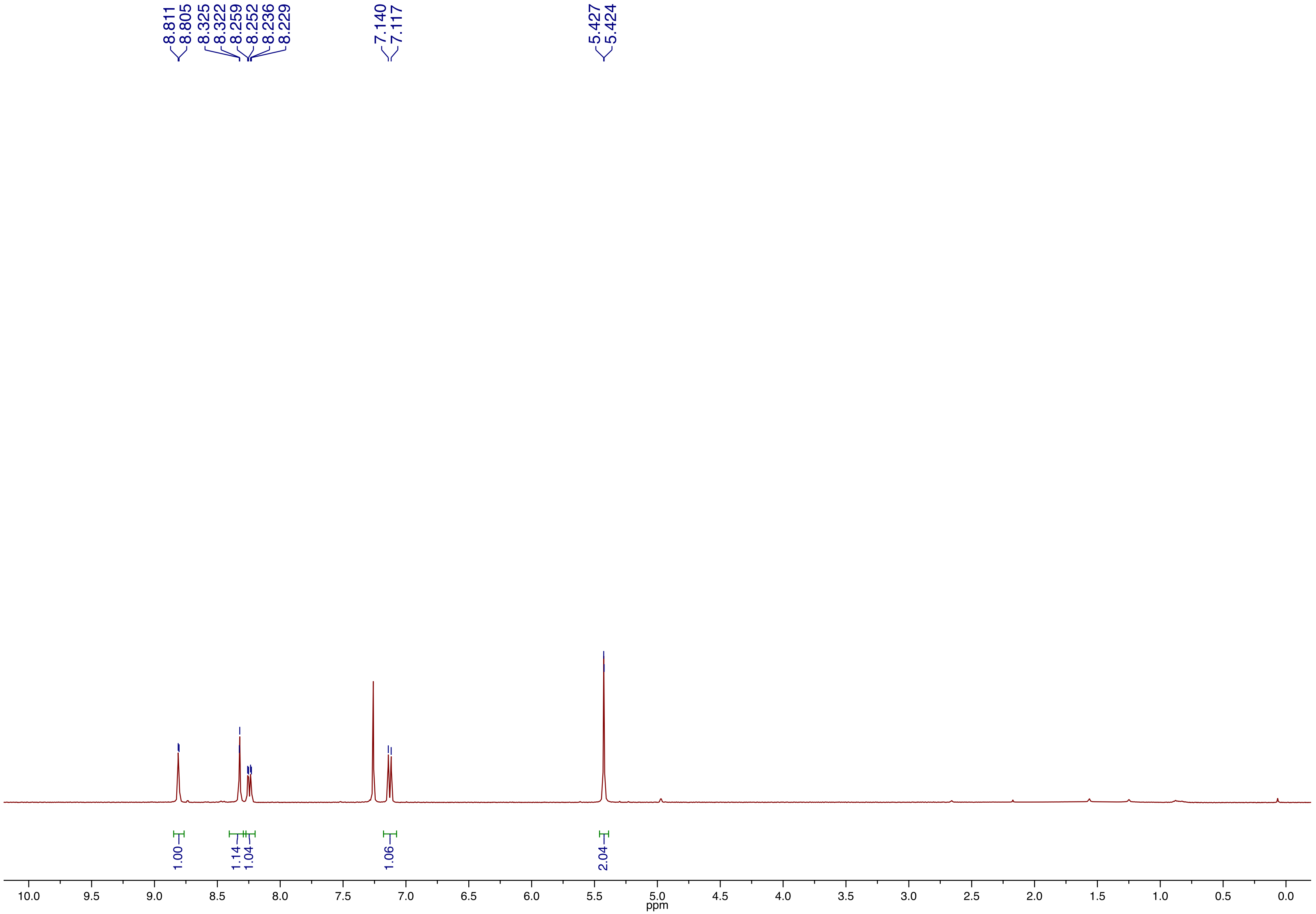

^{13}C NMR (101 MHz, $CDCl_3$)

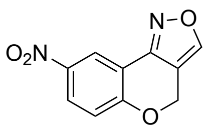

3f

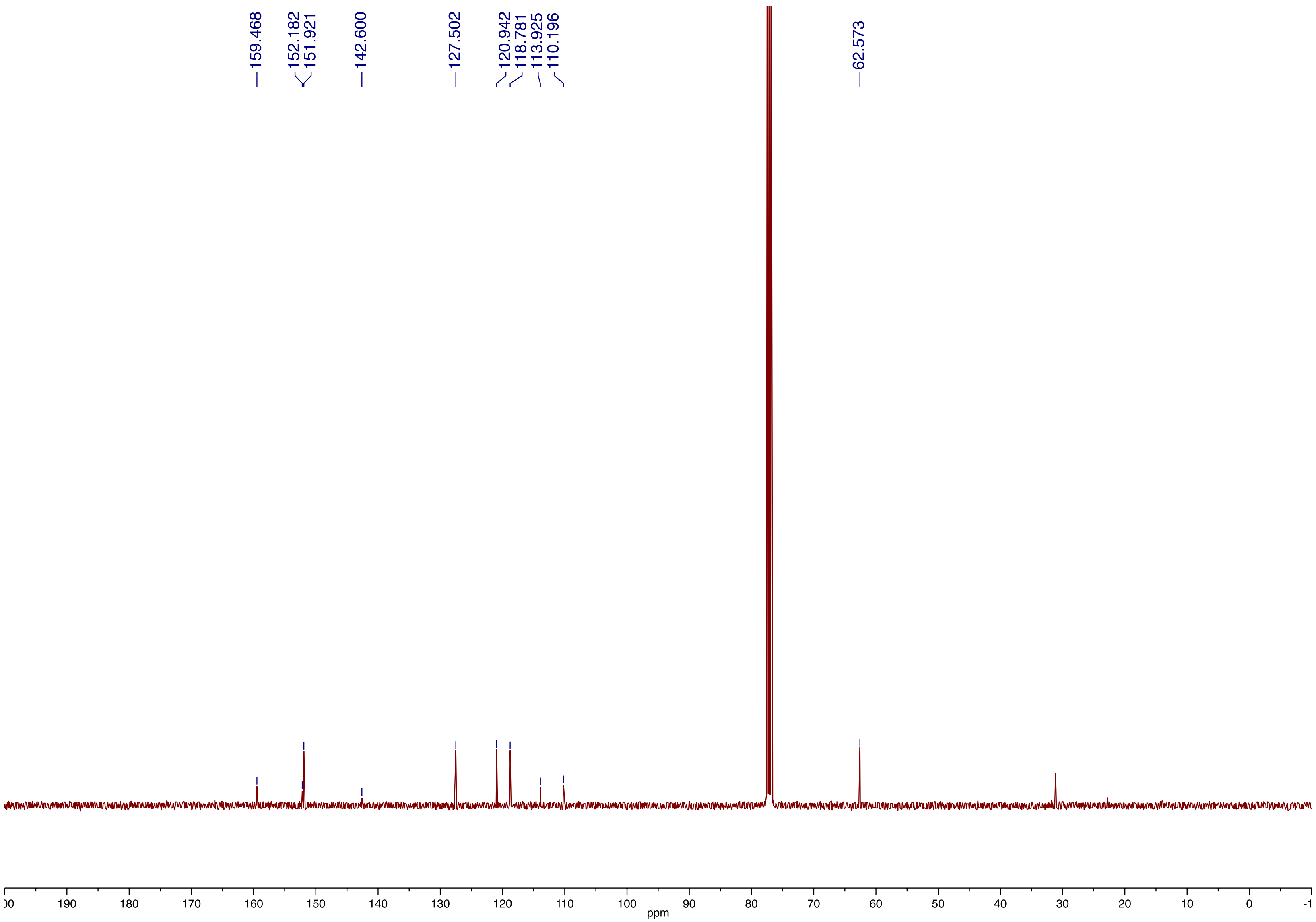

^{1}H NMR (400 MHz, $CDCl_3$)

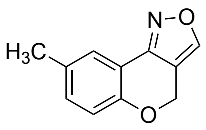

3g

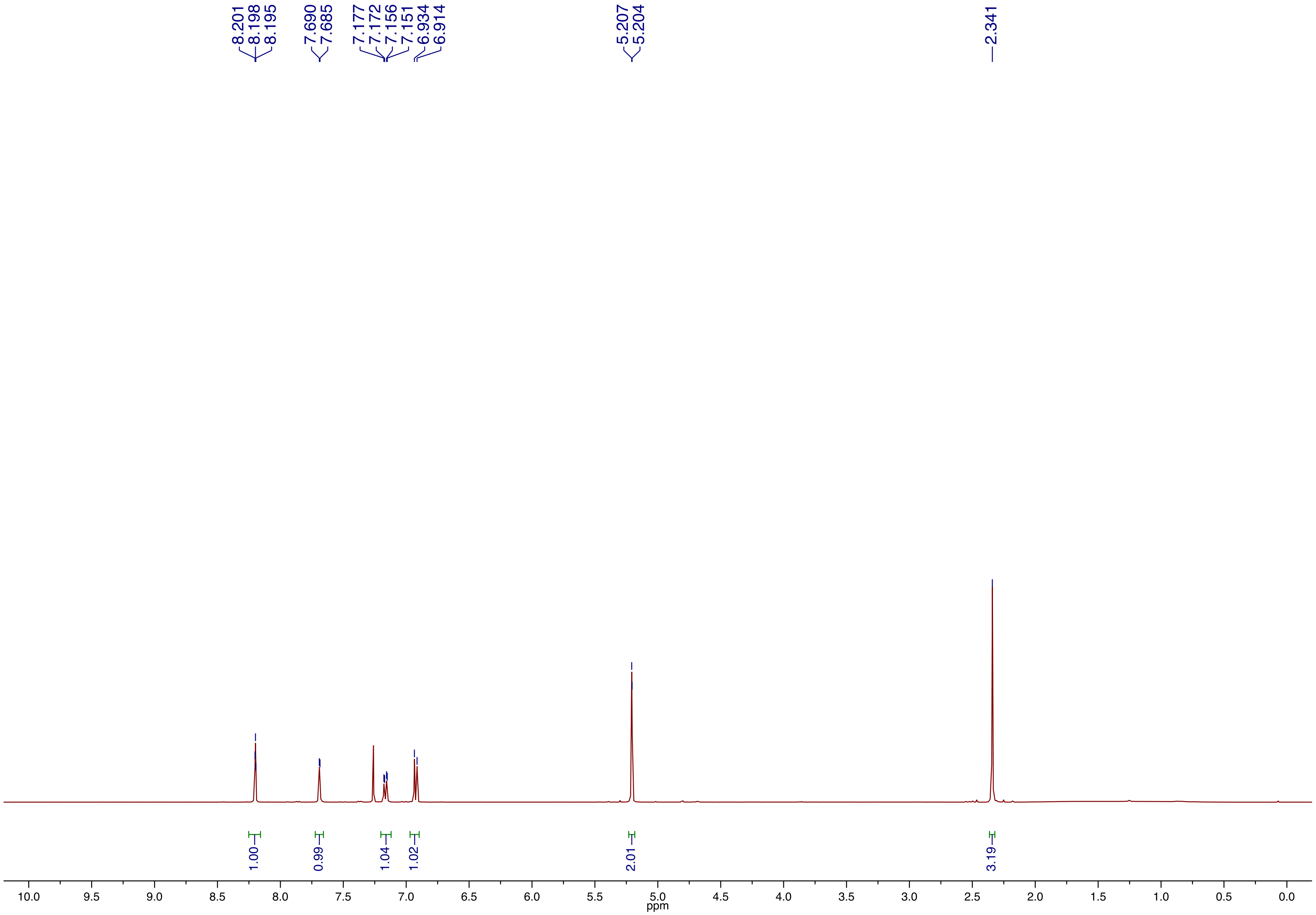

^{13}C NMR (101 MHz, $CDCl_3$)

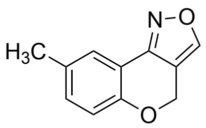

3g

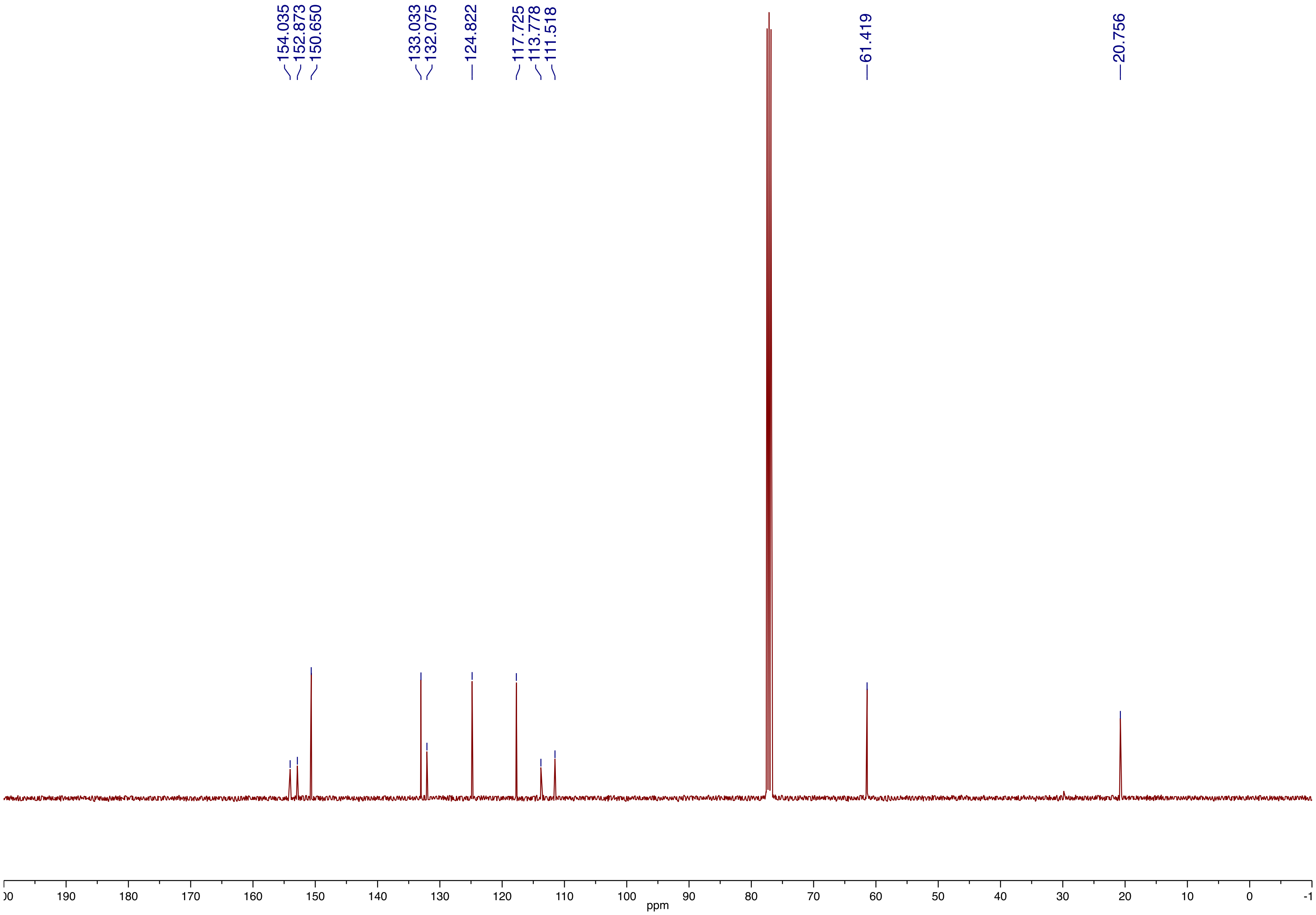

^{1}H NMR (400 MHz, $CDCl_3$)

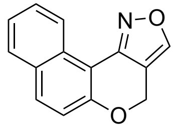

3h

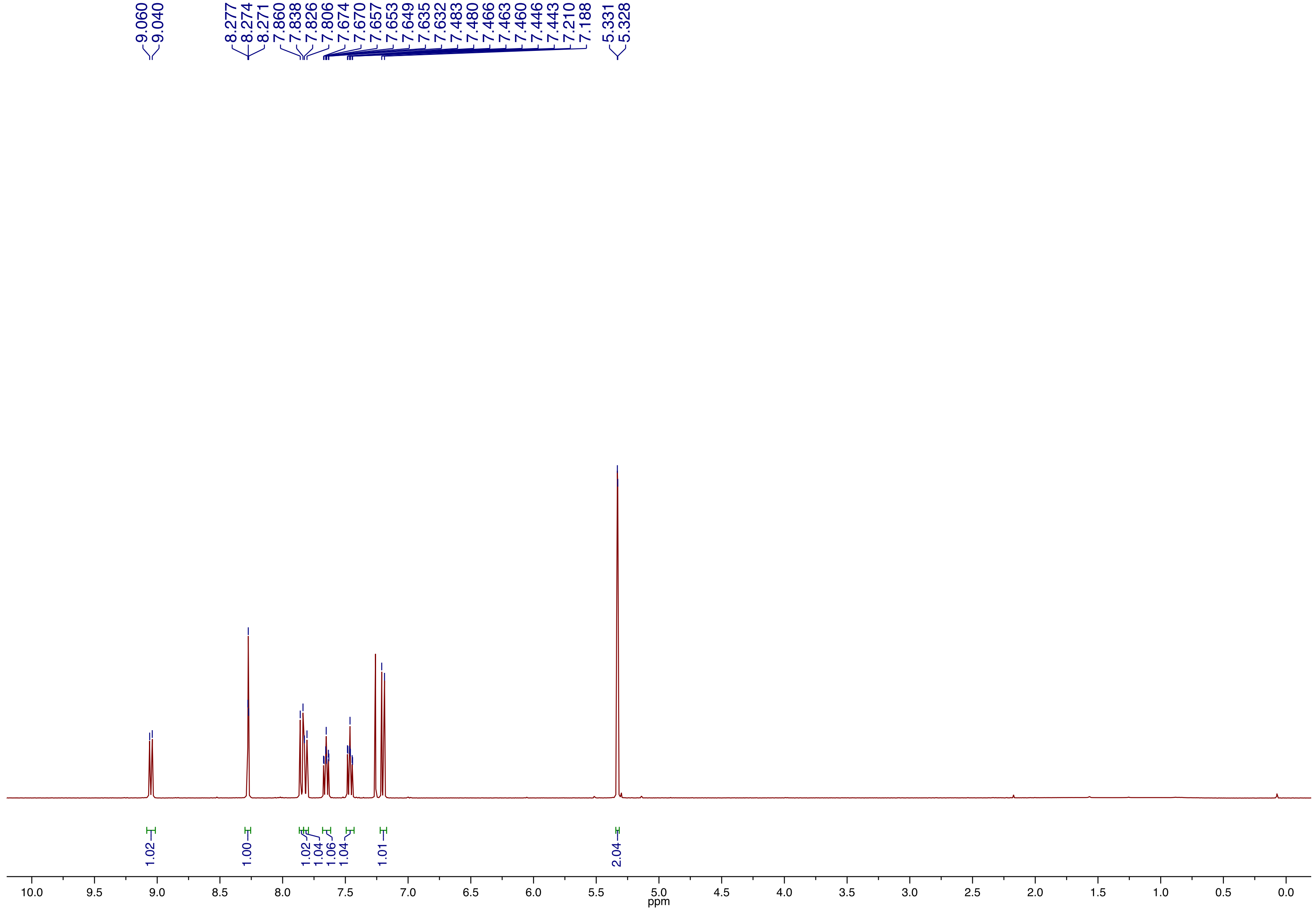

^{13}C NMR (101 MHz, $CDCl_3$)

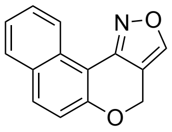

3h

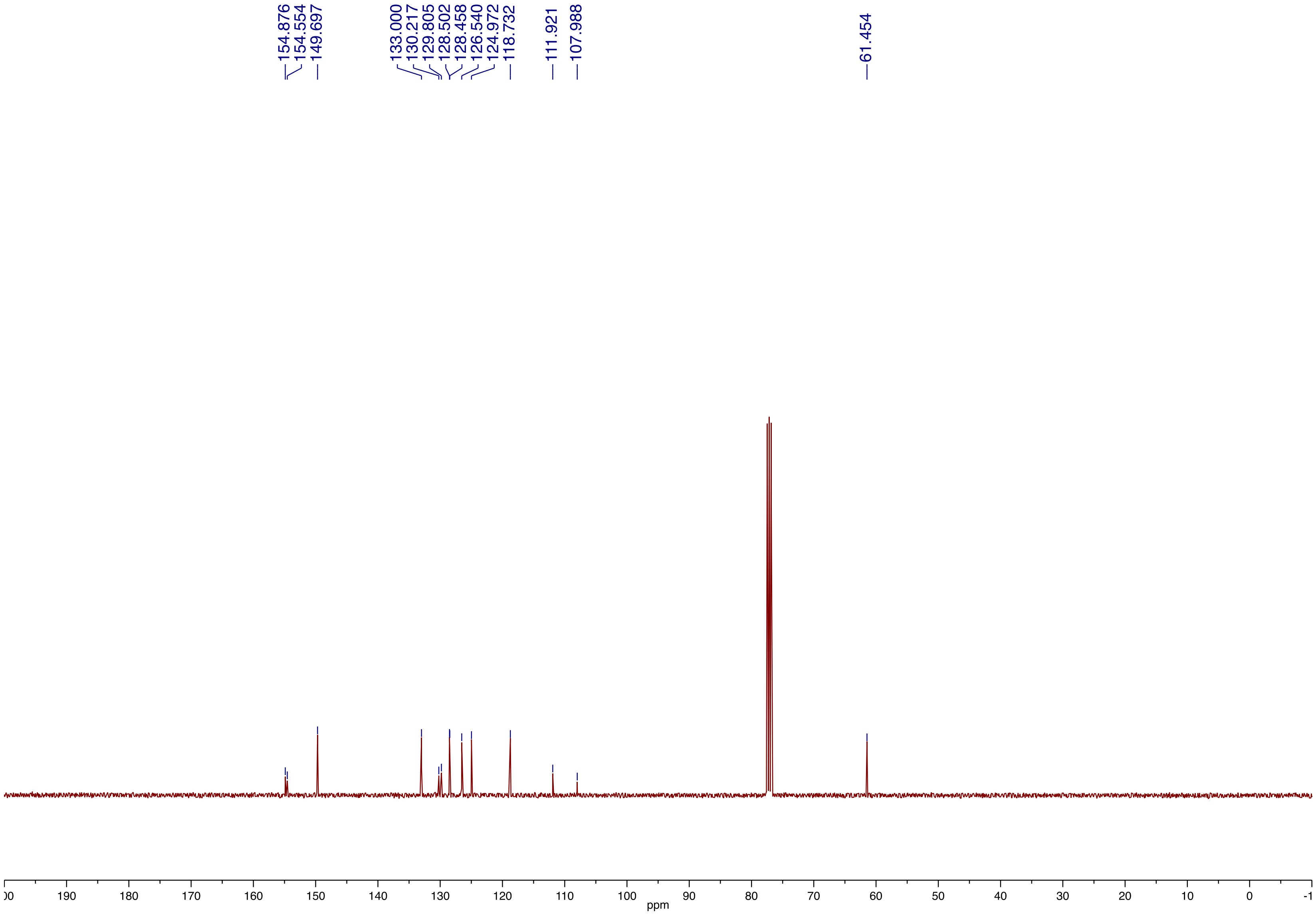

^1H NMR (400 MHz, $CDCl_3$)

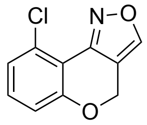

3i

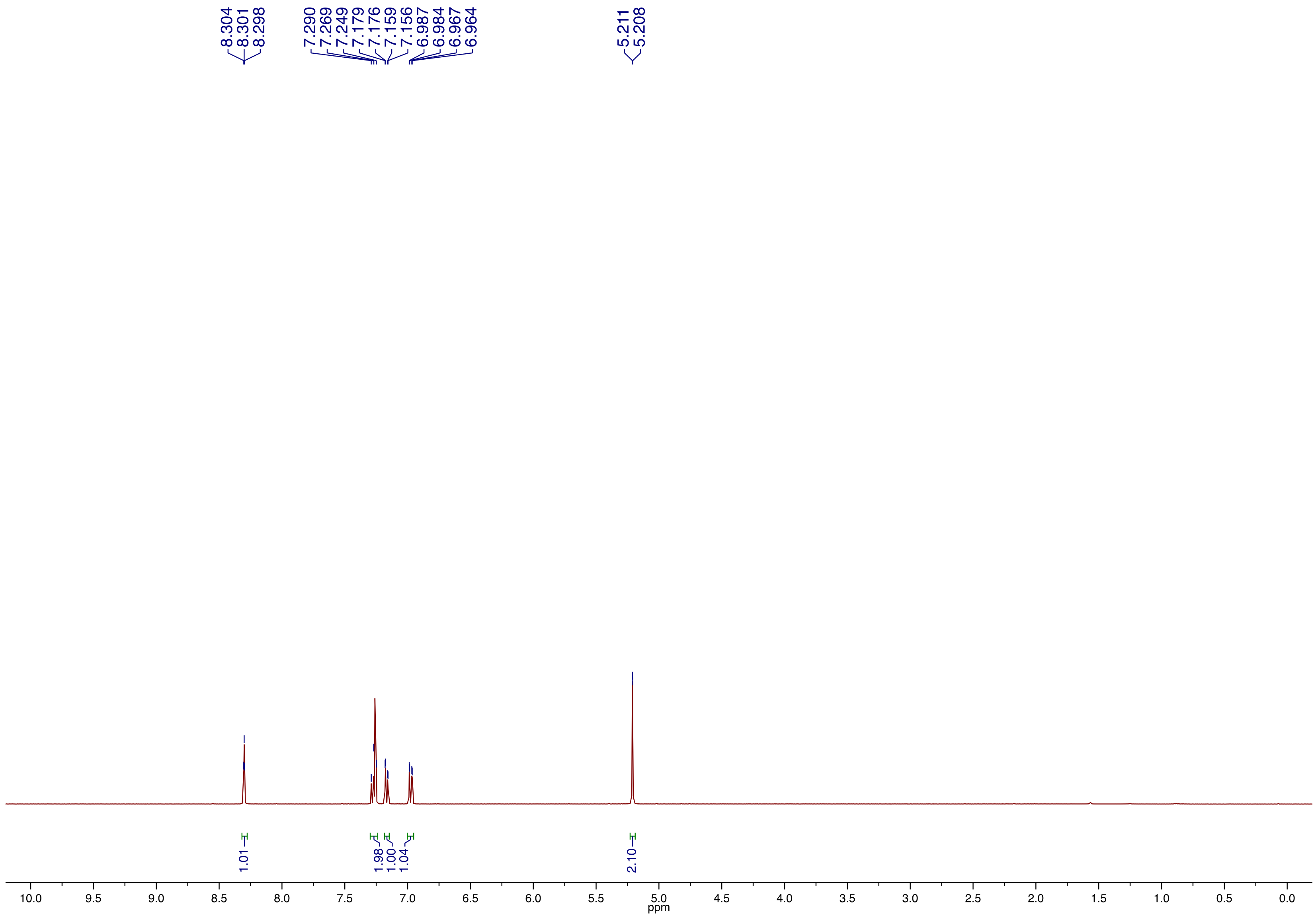

^{13}C NMR (101 MHz, $CDCl_3$)

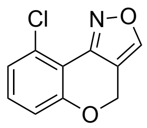

3i

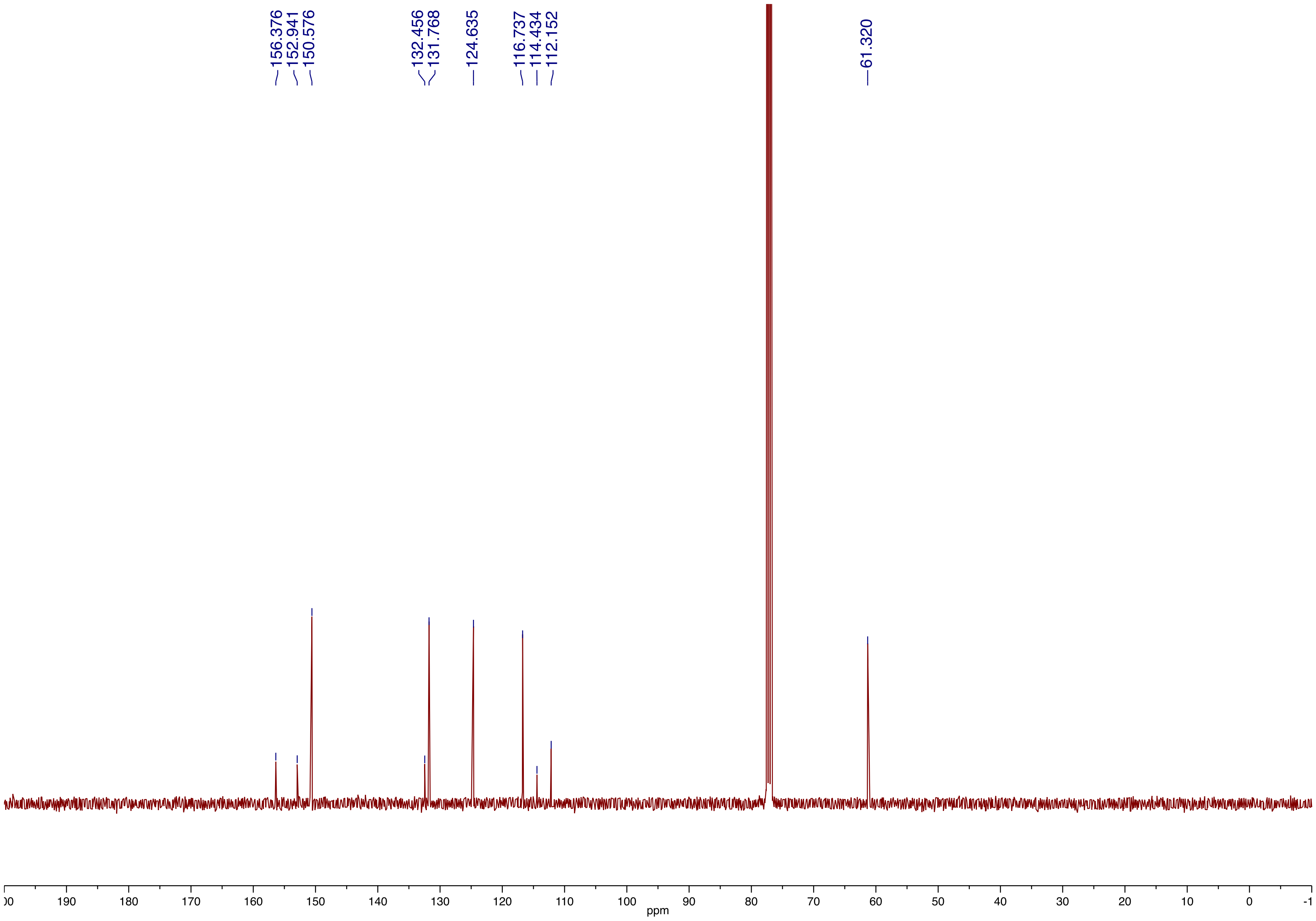

^{1}H NMR (400 MHz, $CDCl_3$)

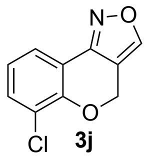

3j

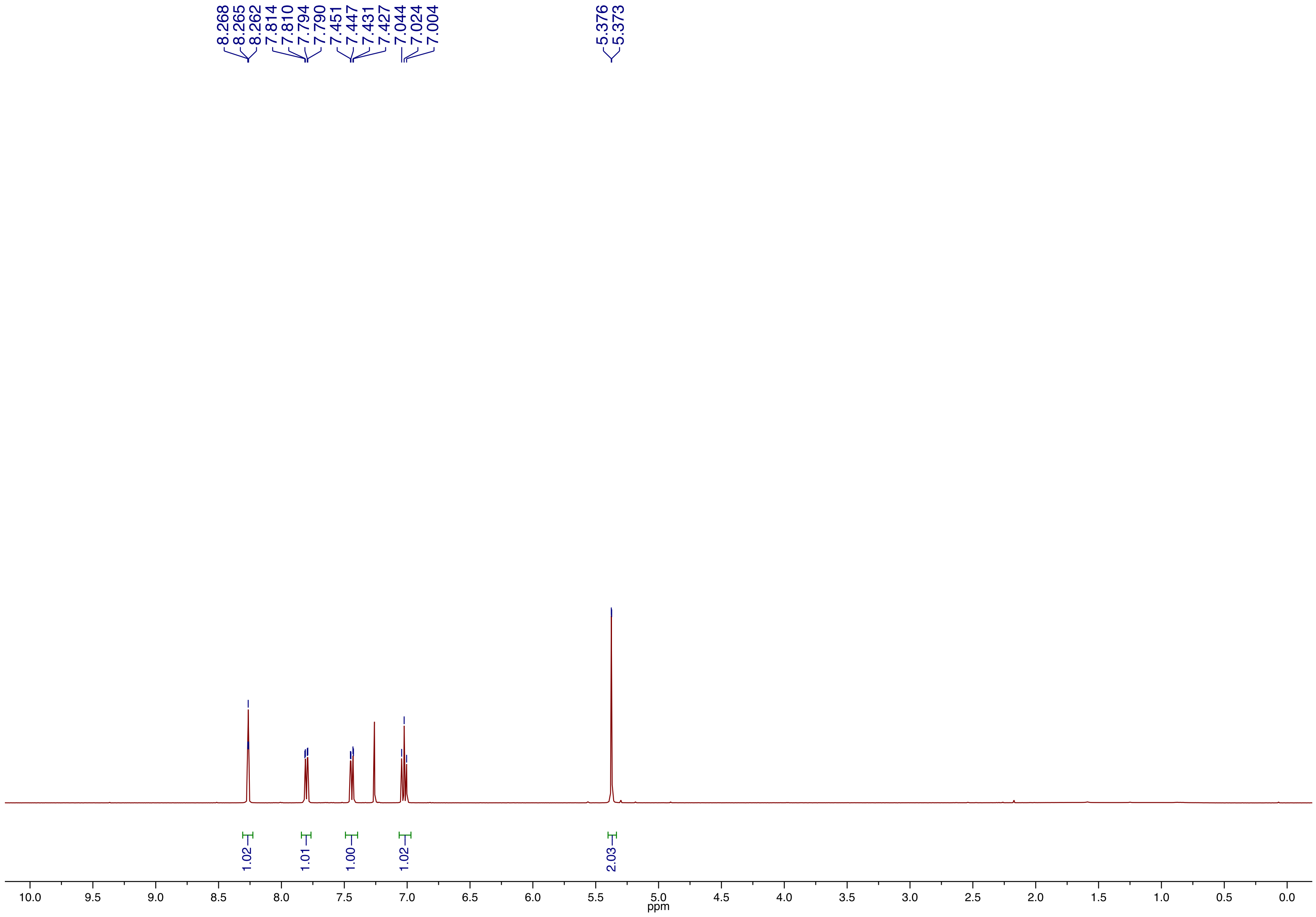

^{13}C NMR (101 MHz, $CDCl_3$)

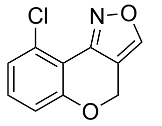

3i

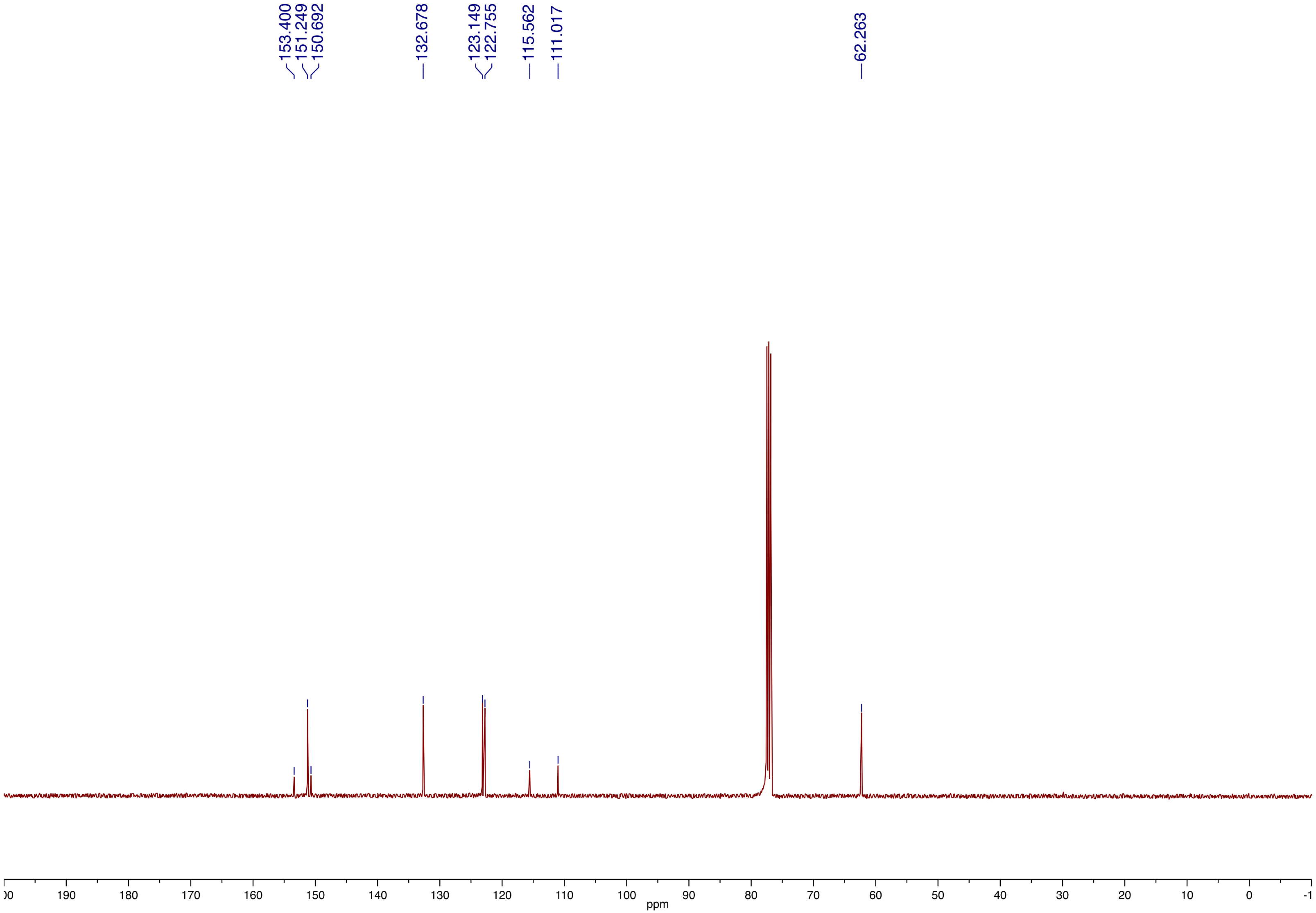

^{1}H NMR (400 MHz, $CDCl_3$)

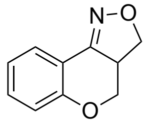

3k

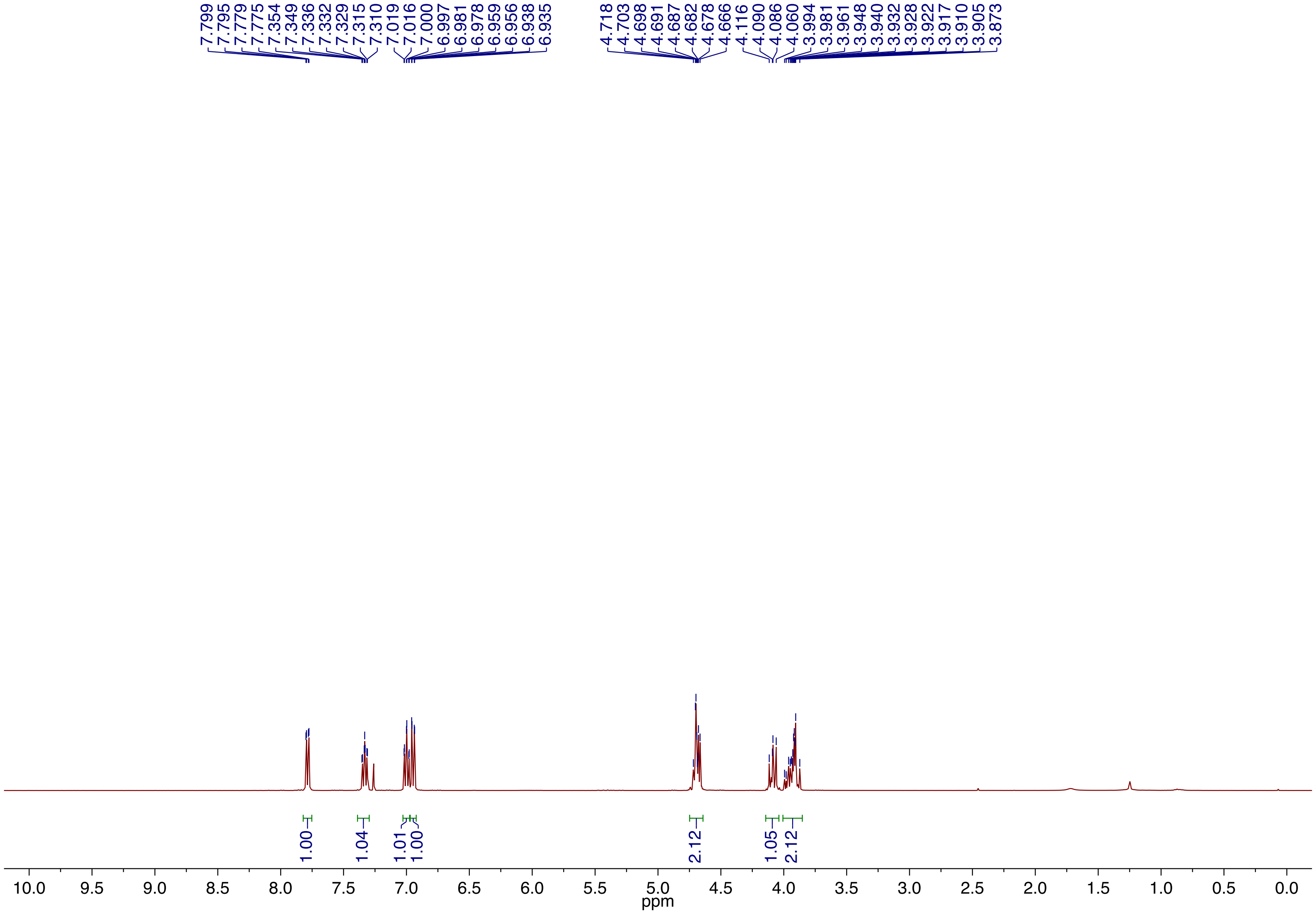

^{13}C NMR (101 MHz, $CDCl_3$)

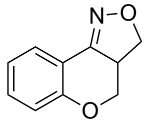

3k

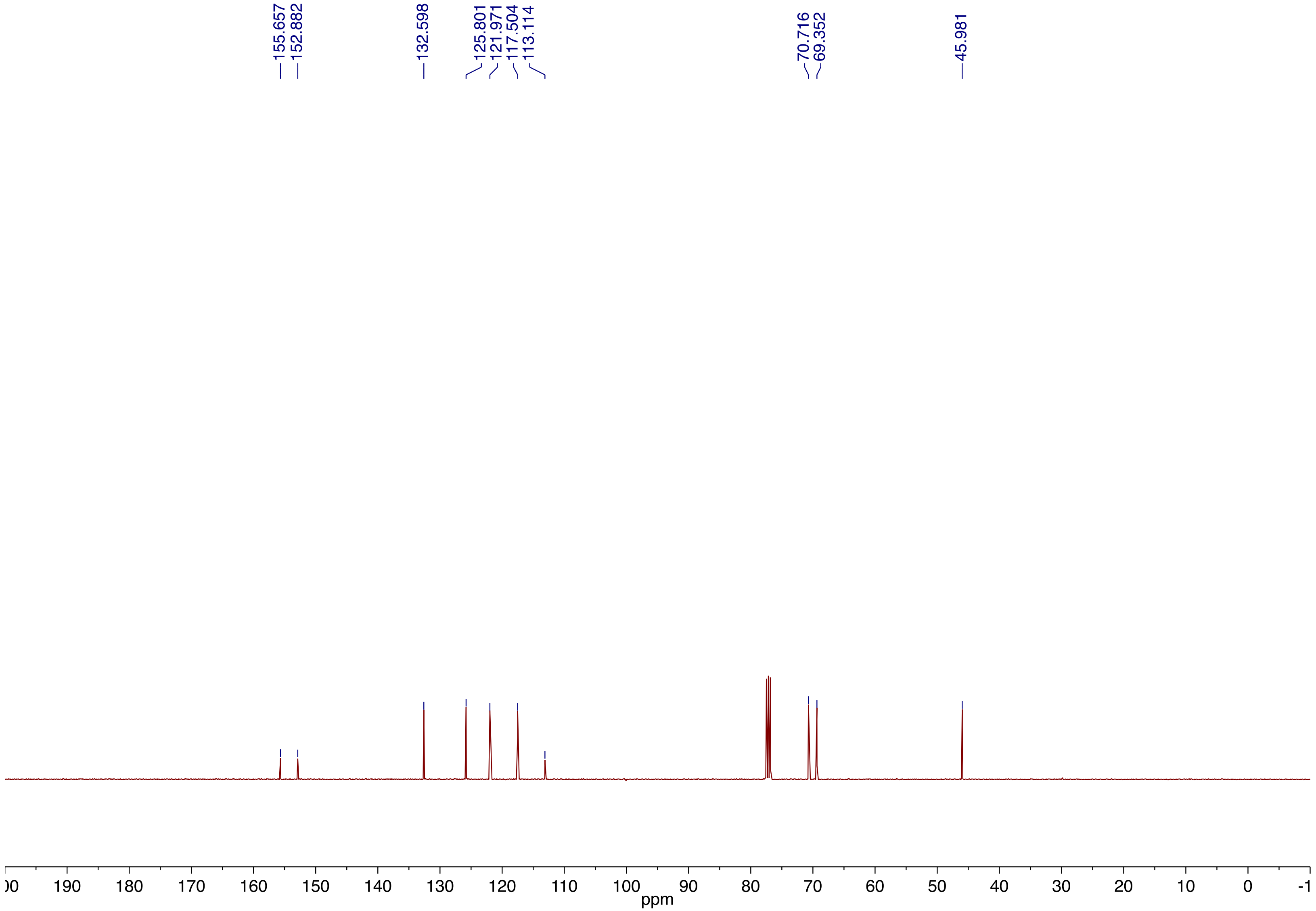

^{1}H NMR (400 MHz, $CDCl_3$)

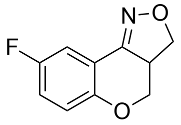

3l

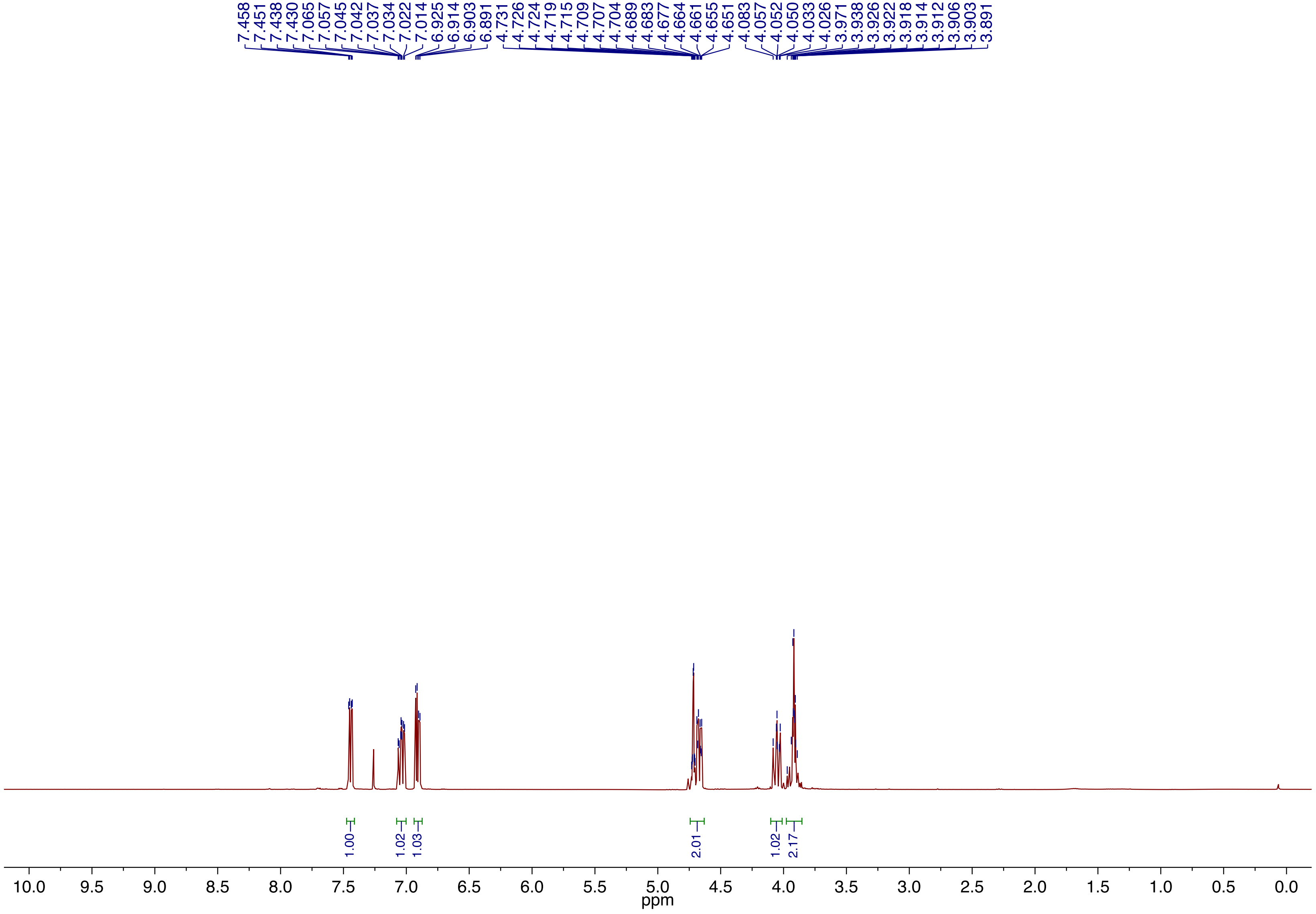

^{13}C NMR (101 MHz, $CDCl_3$)

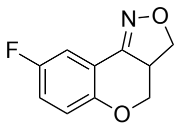

3l

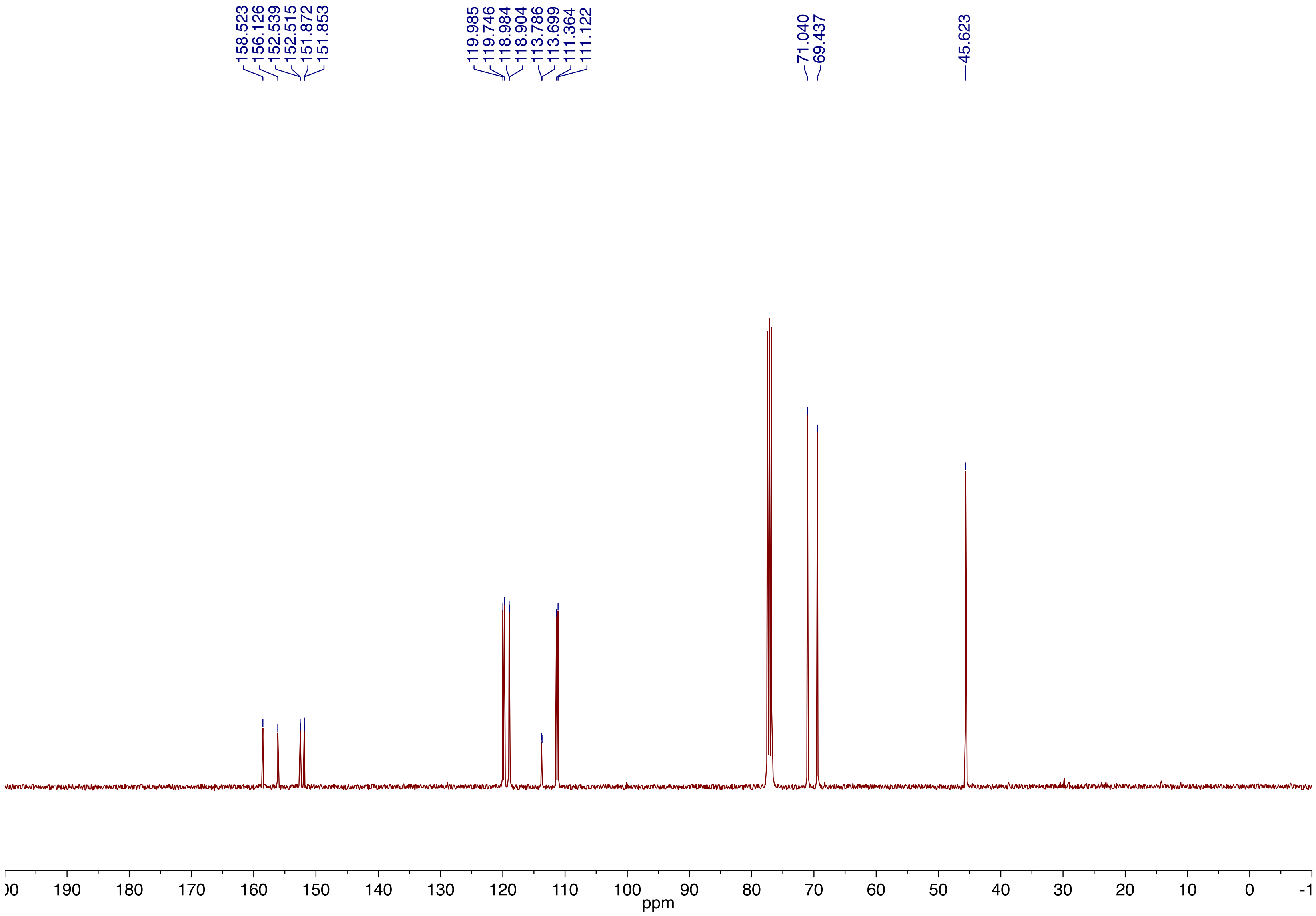

^{1}H NMR (400 MHz, $CDCl_3$)

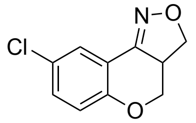

3m

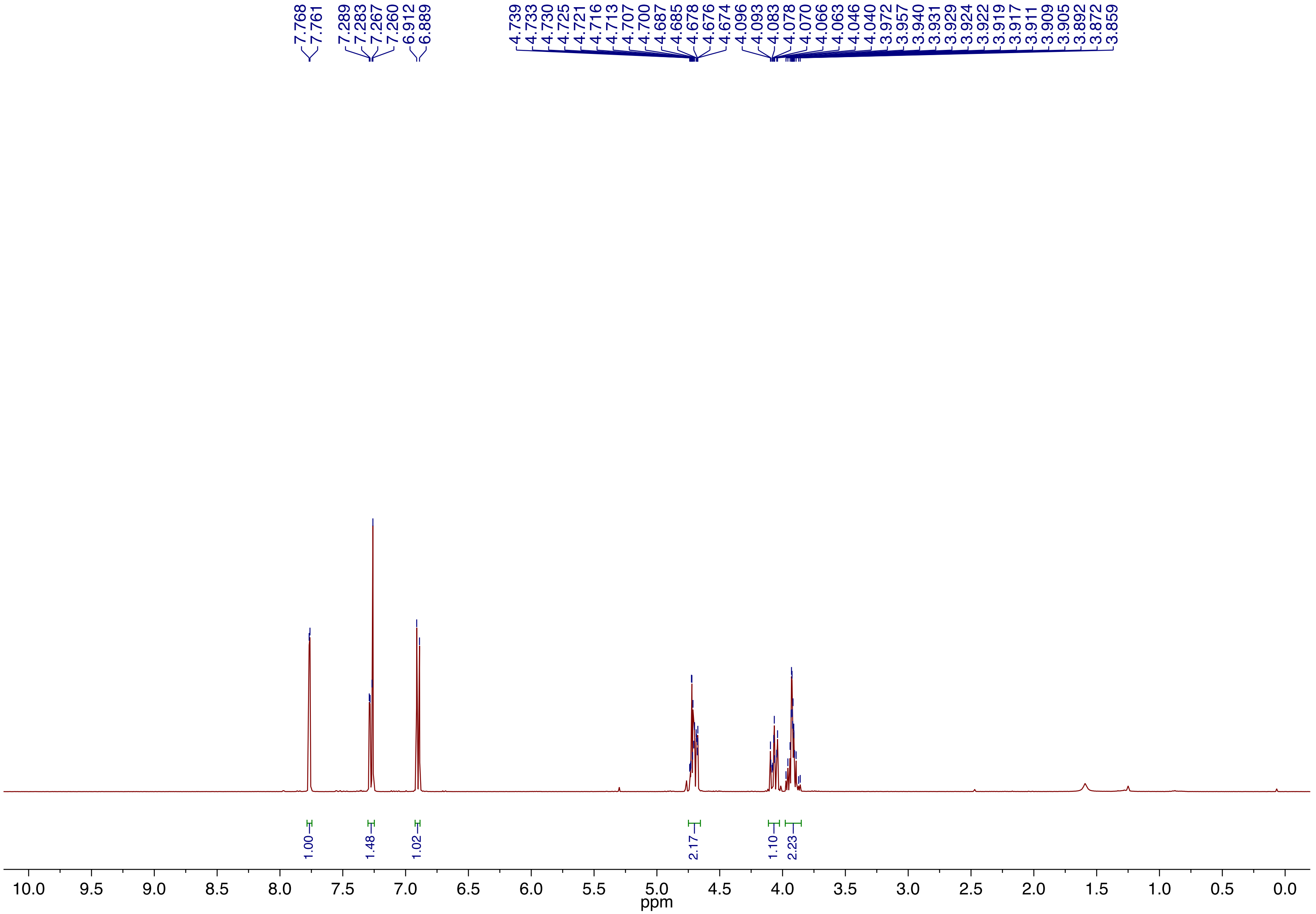

^{13}C NMR (101 MHz, $CDCl_3$)

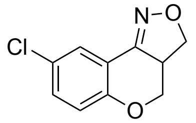

3m

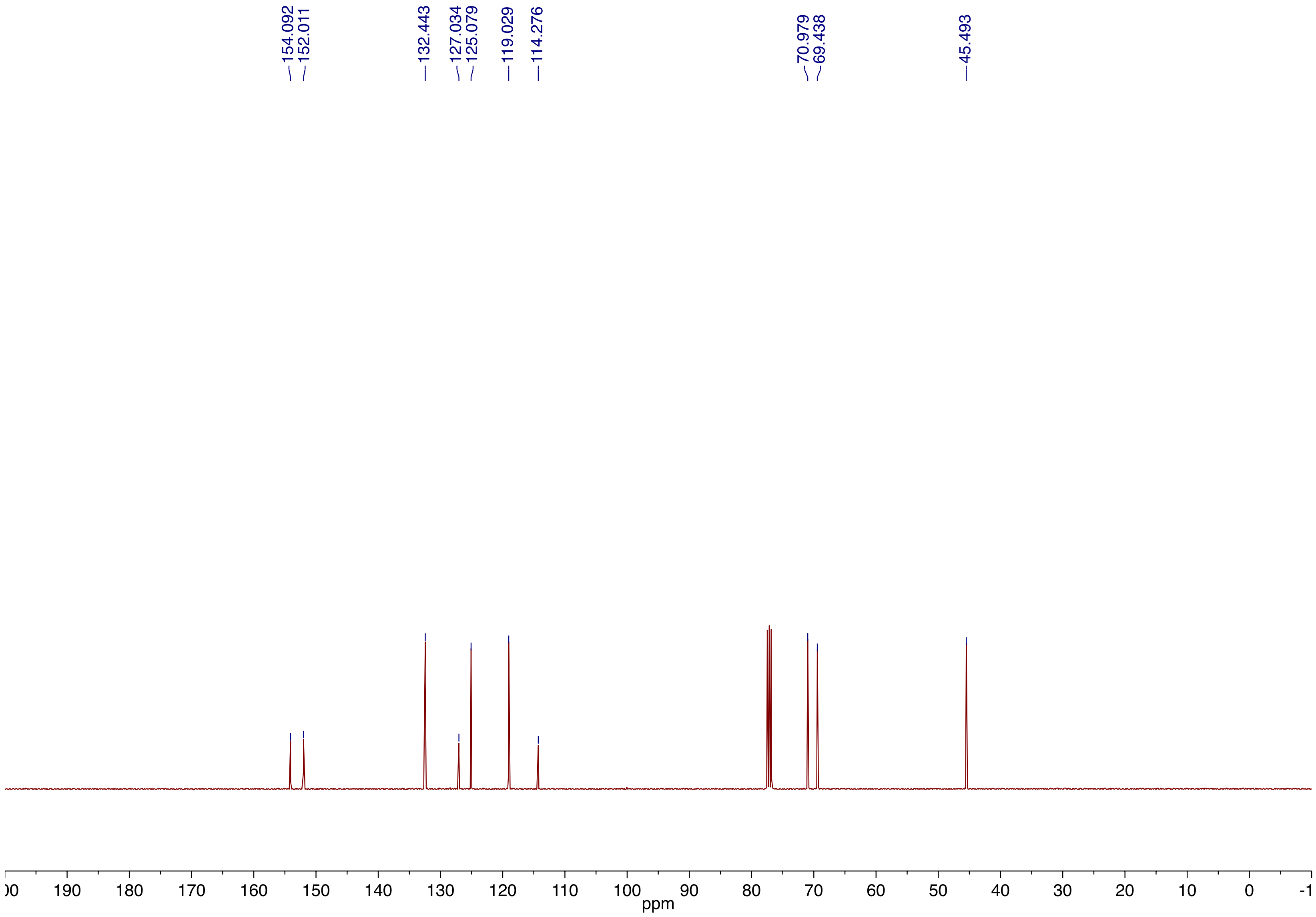

^{1}H NMR (400 MHz, $CDCl_3$)

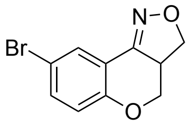

3n

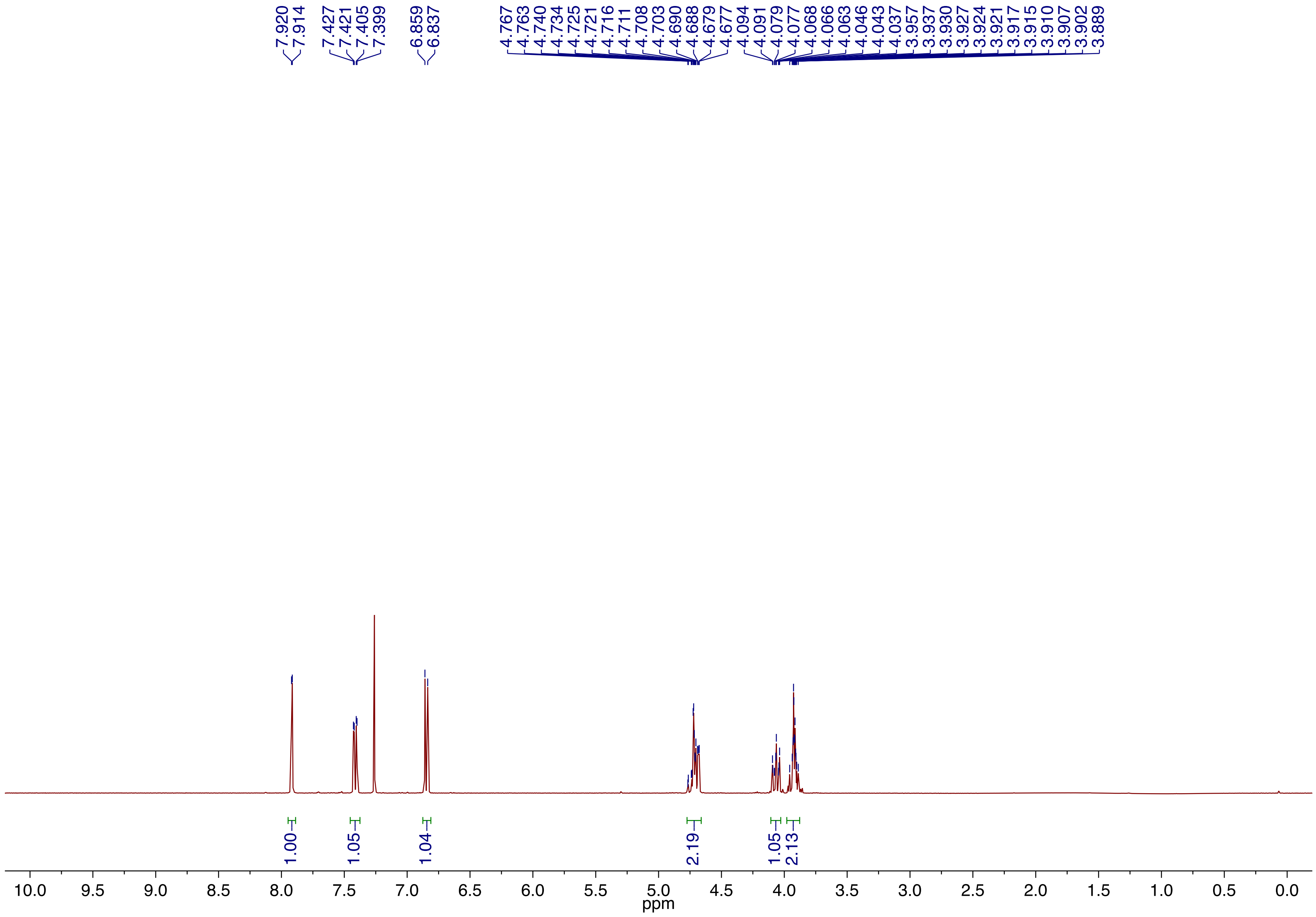

^{13}C NMR (101 MHz, $CDCl_3$)

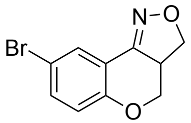

3n

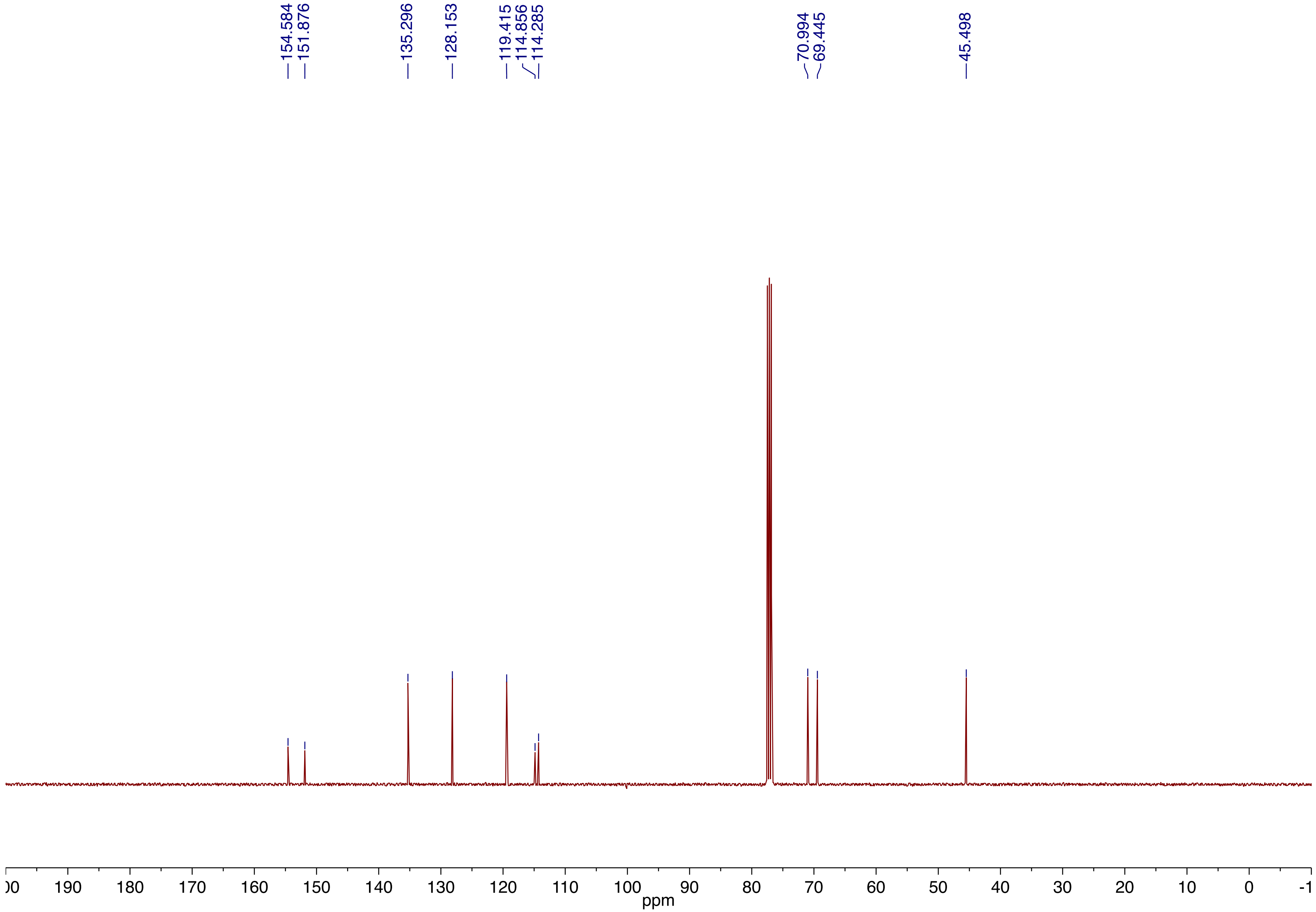

^{1}H NMR (400 MHz, $CDCl_3$)

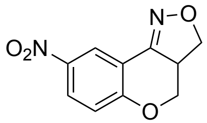

3o

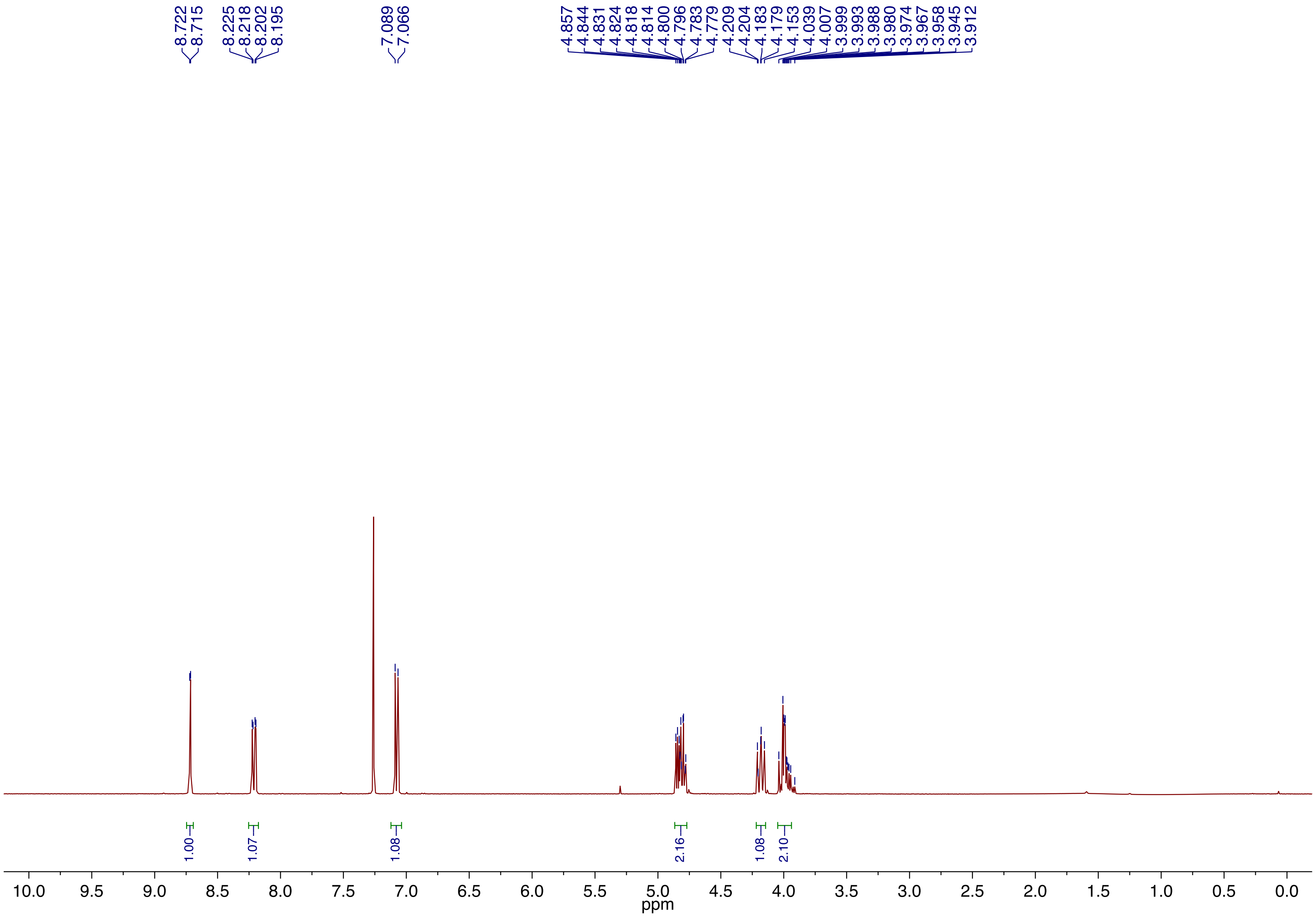

^{13}C NMR (101 MHz, $CDCl_3$)

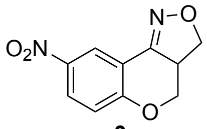

3o

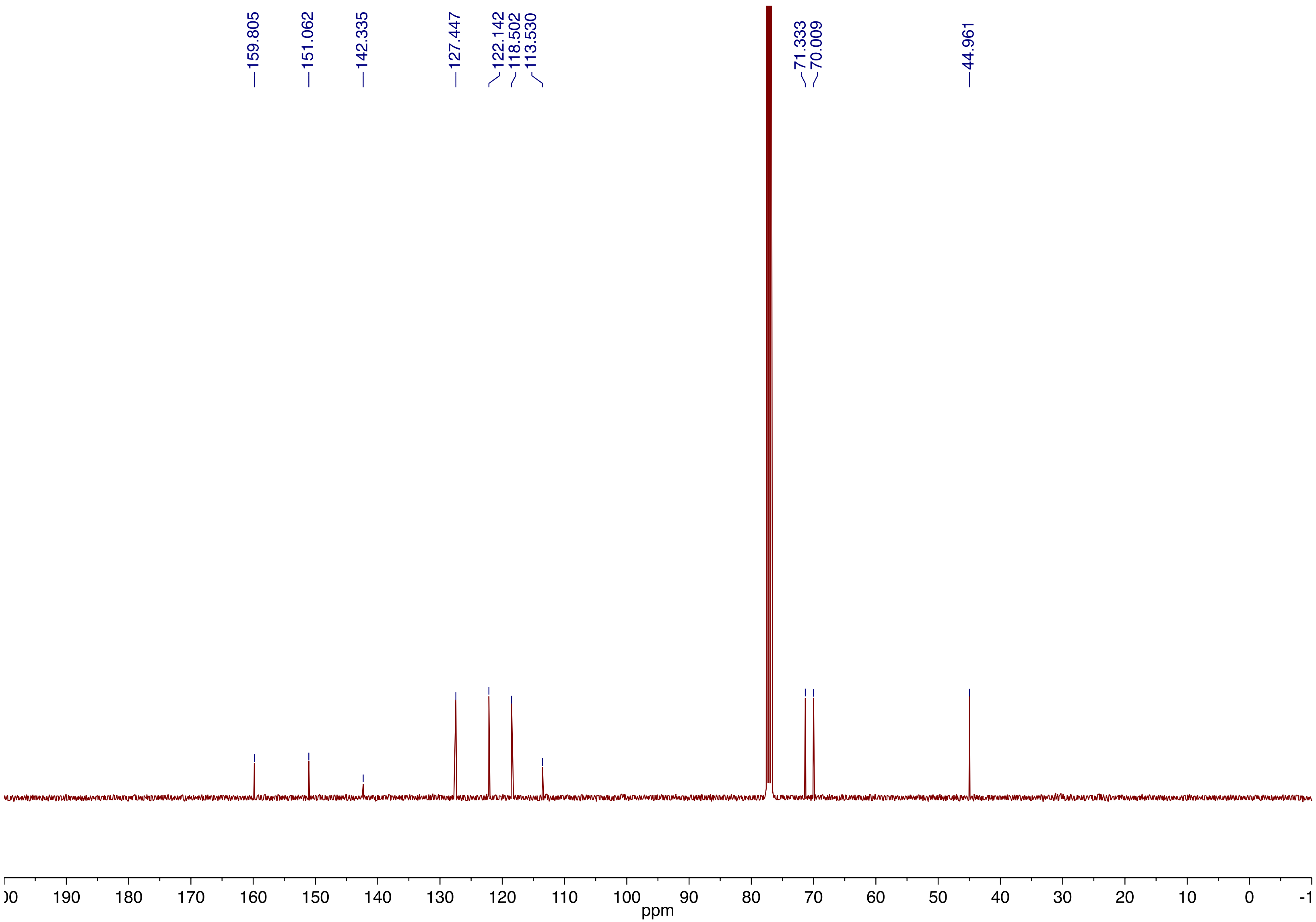

¹H NMR (400 MHz, $CDCl_3$)

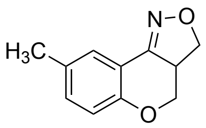

3p

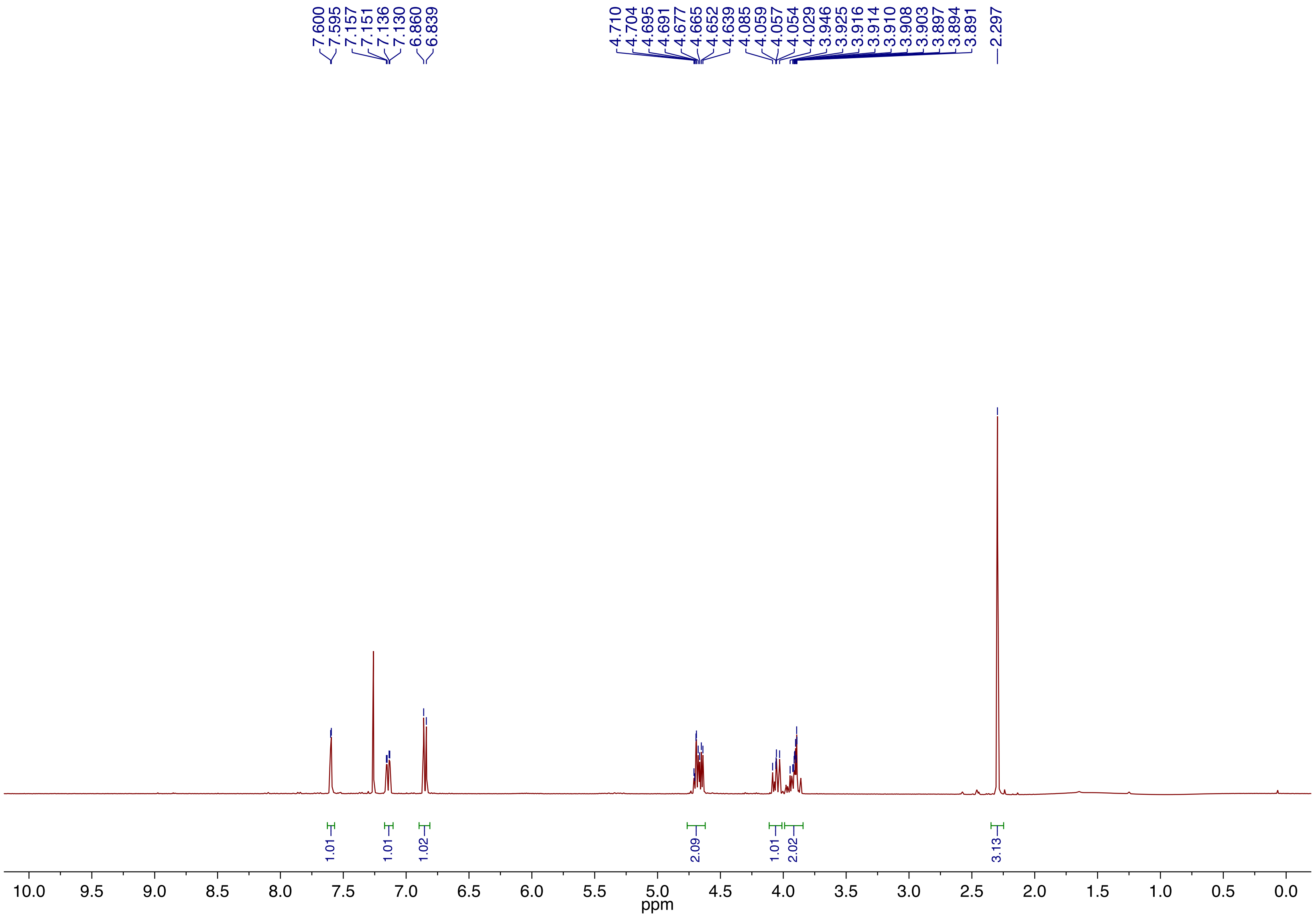

^{13}C NMR (101 MHz, $CDCl_3$)

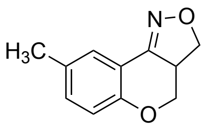

3p

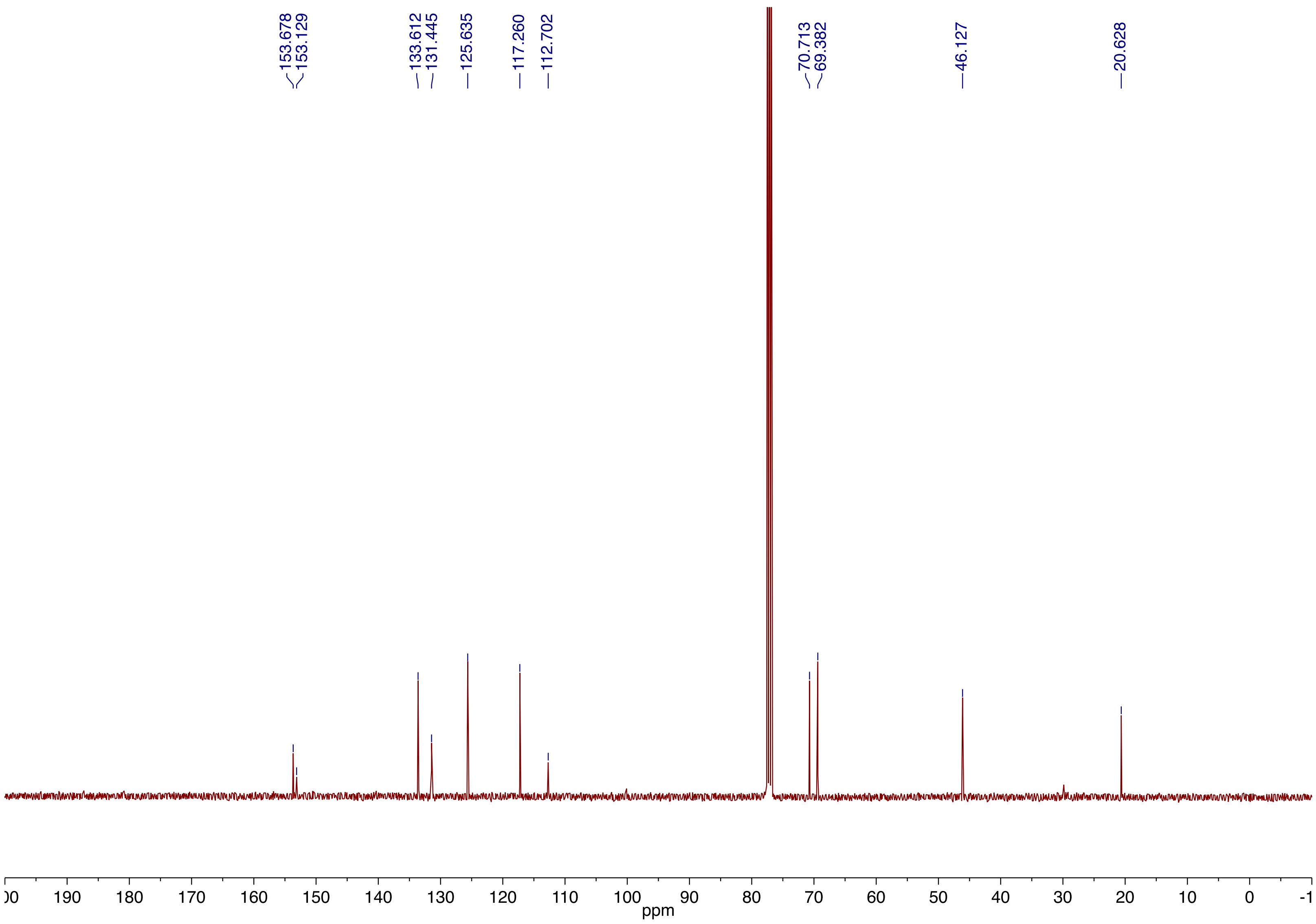

^{1}H NMR (400 MHz, $CDCl_3$)

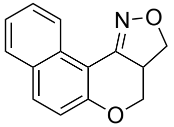

3q

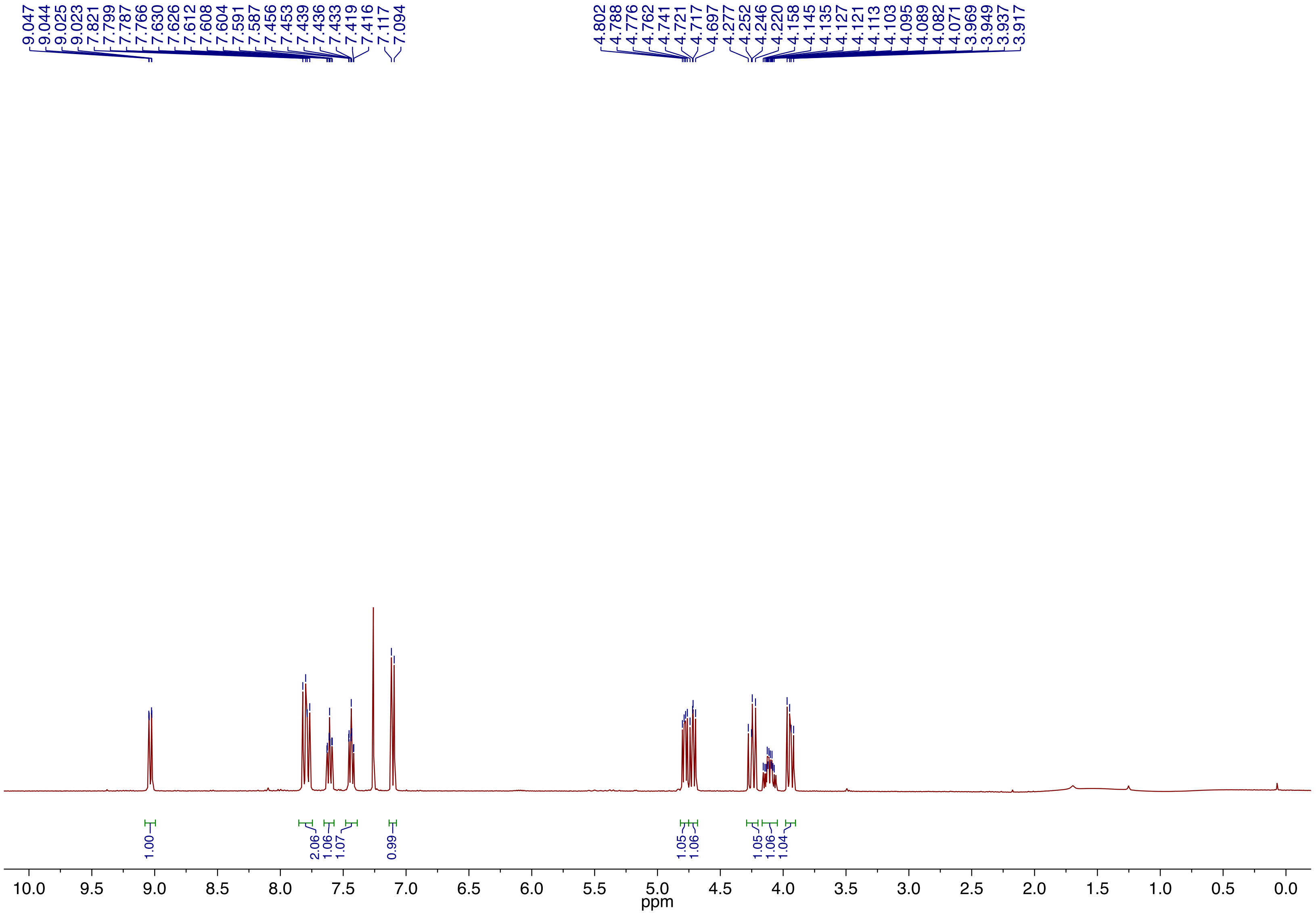

^{13}C NMR (101 MHz, $CDCl_3$)

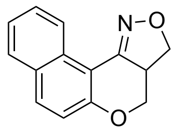

3q

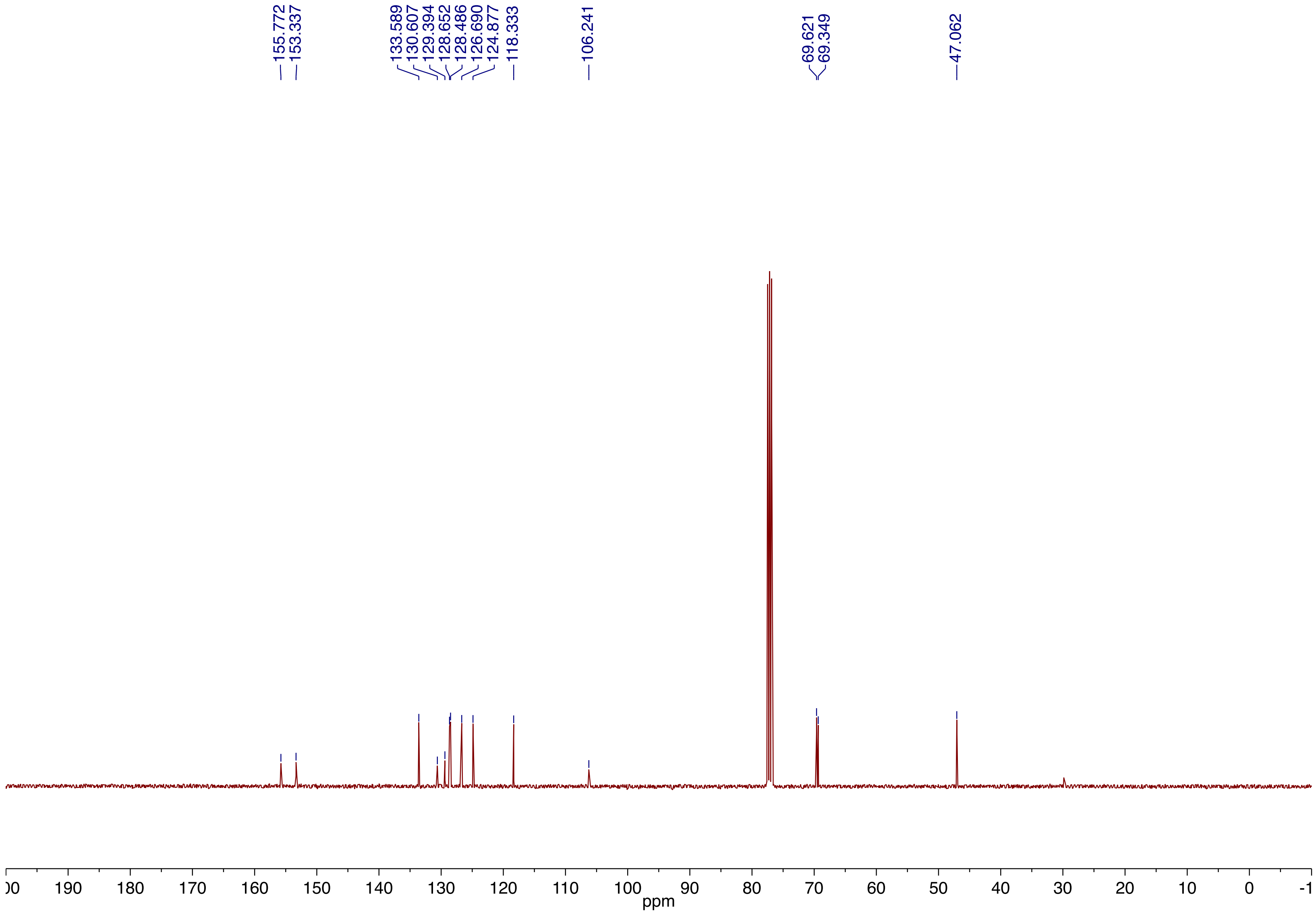

^{1}H NMR (400 MHz, $CDCl_3$)

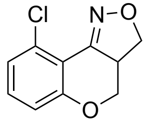

3r

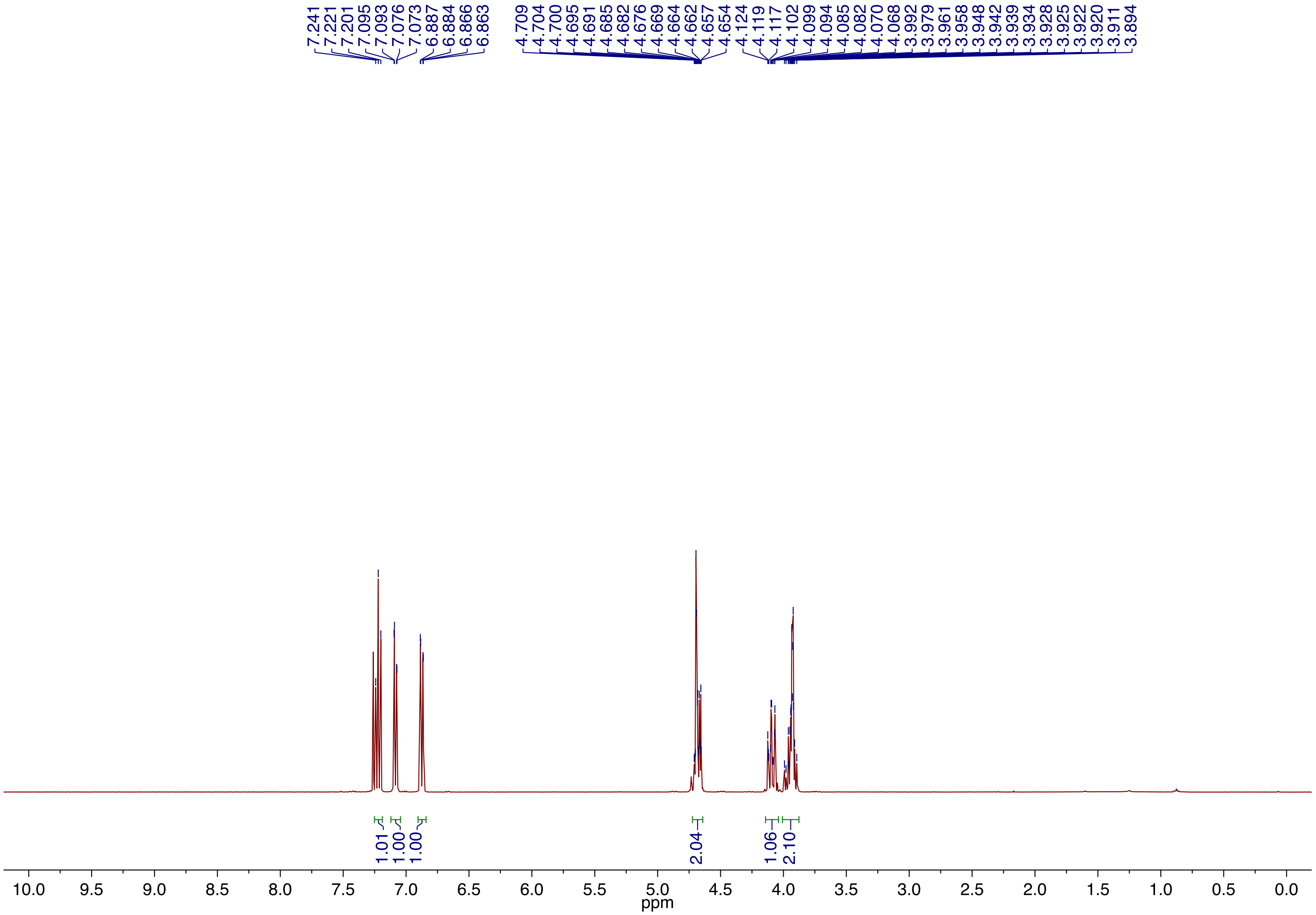

^{13}C NMR (101 MHz, $CDCl_3$)

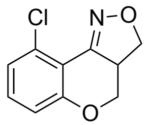

3r

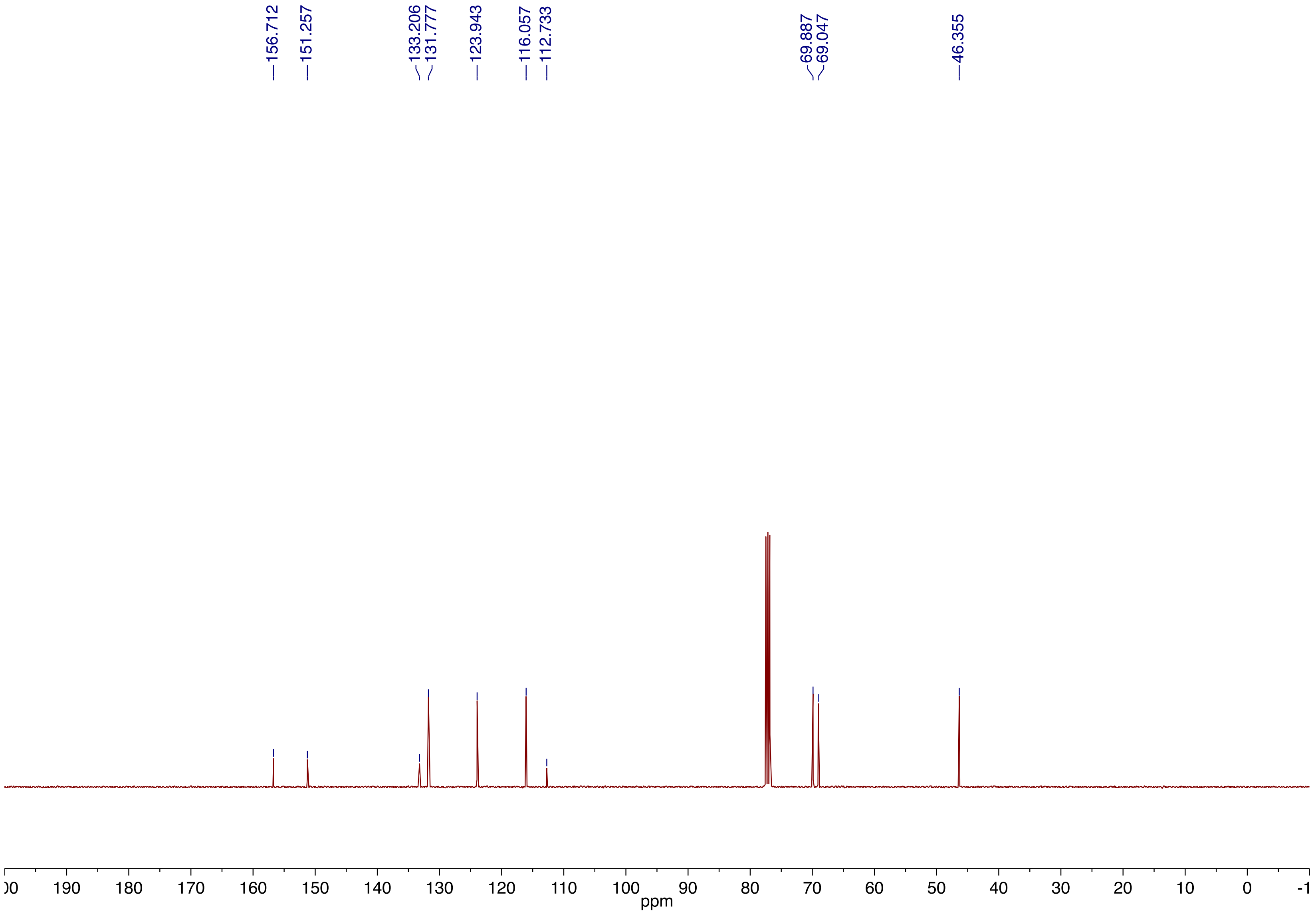

^{1}H NMR (400 MHz, $CDCl_3$)

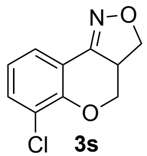

3s

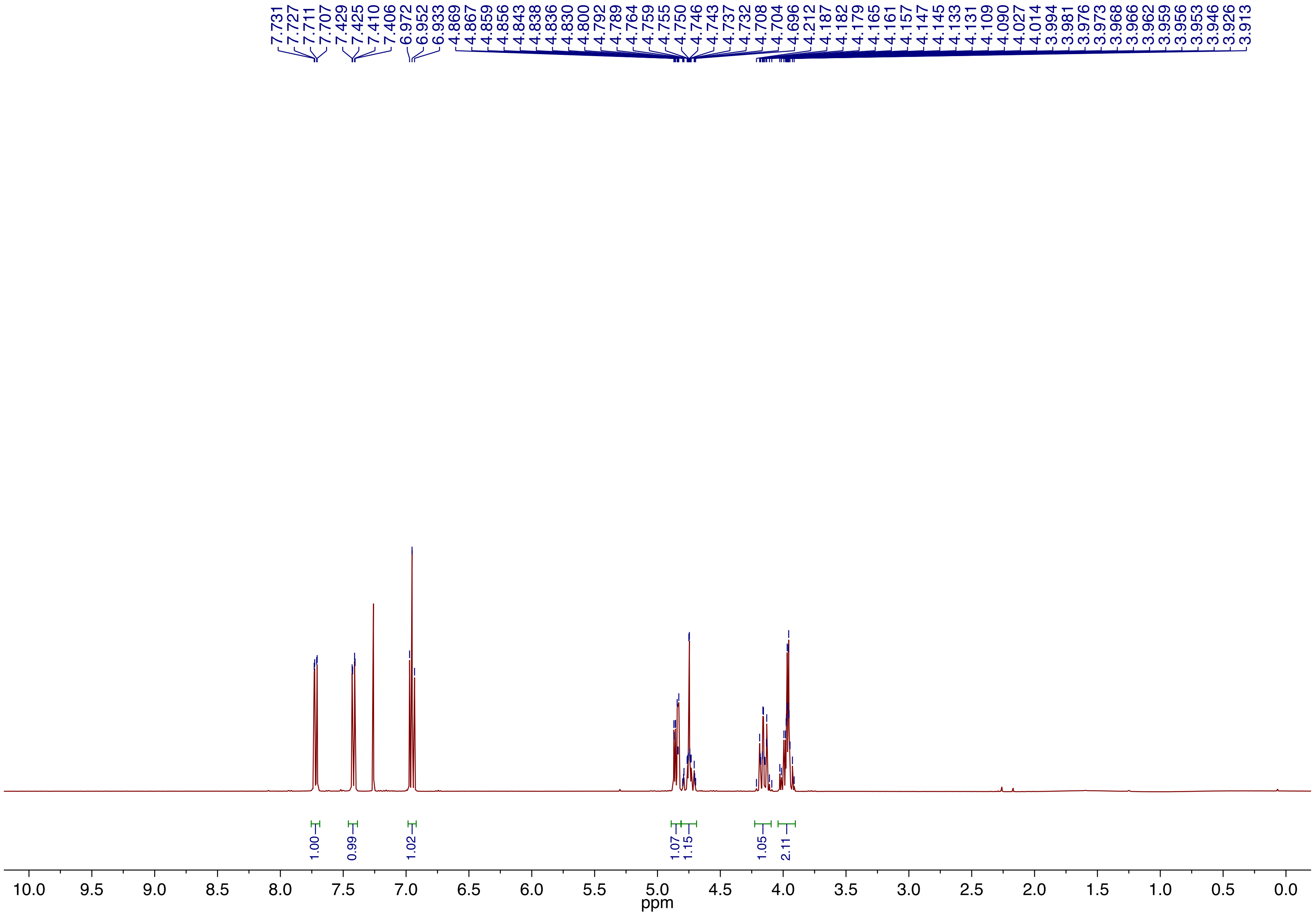

^{13}C NMR (101 MHz, $CDCl_3$)

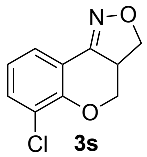

3s

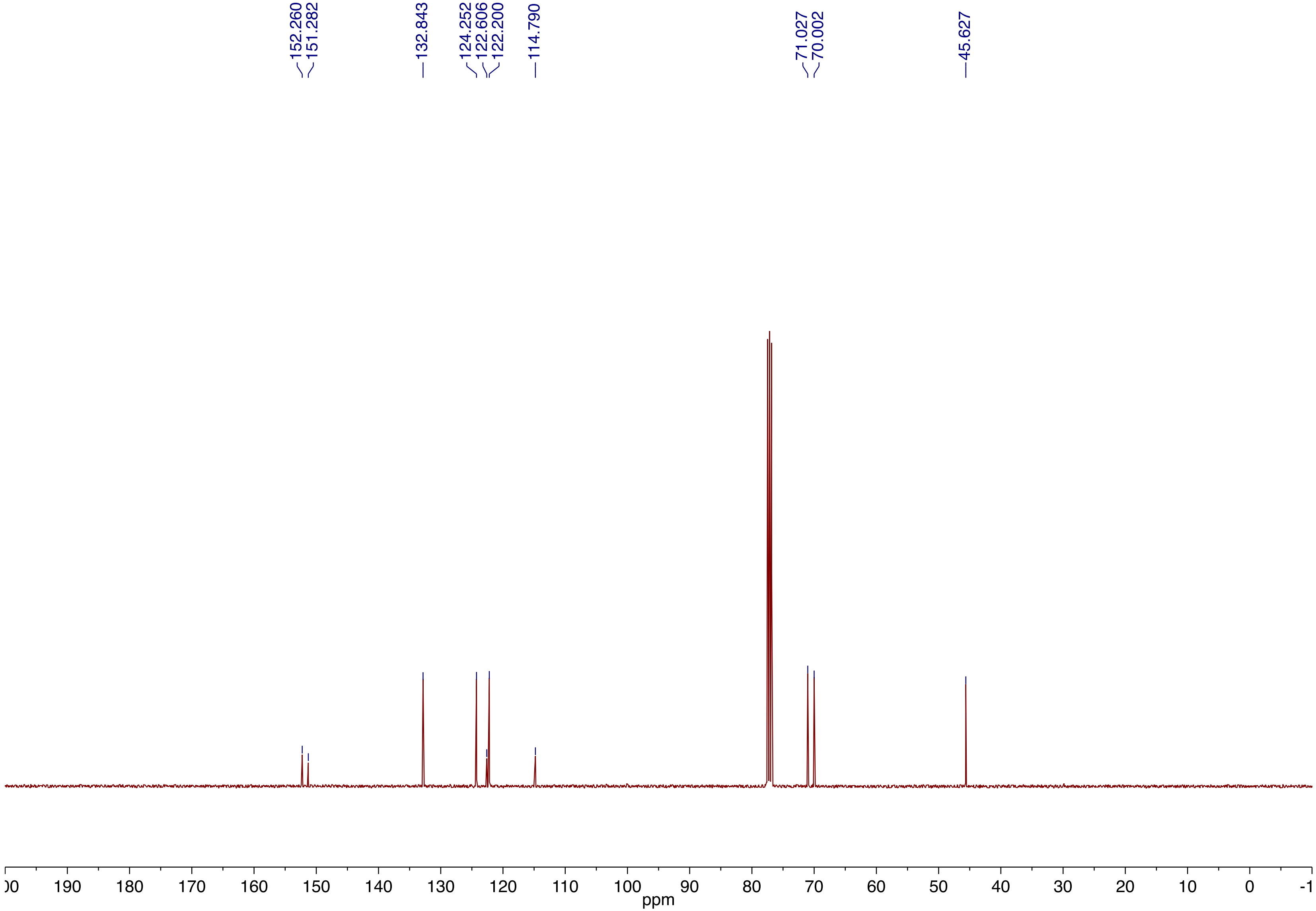

^{1}H NMR (400 MHz, $CDCl_3$)

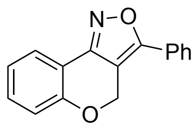

3t

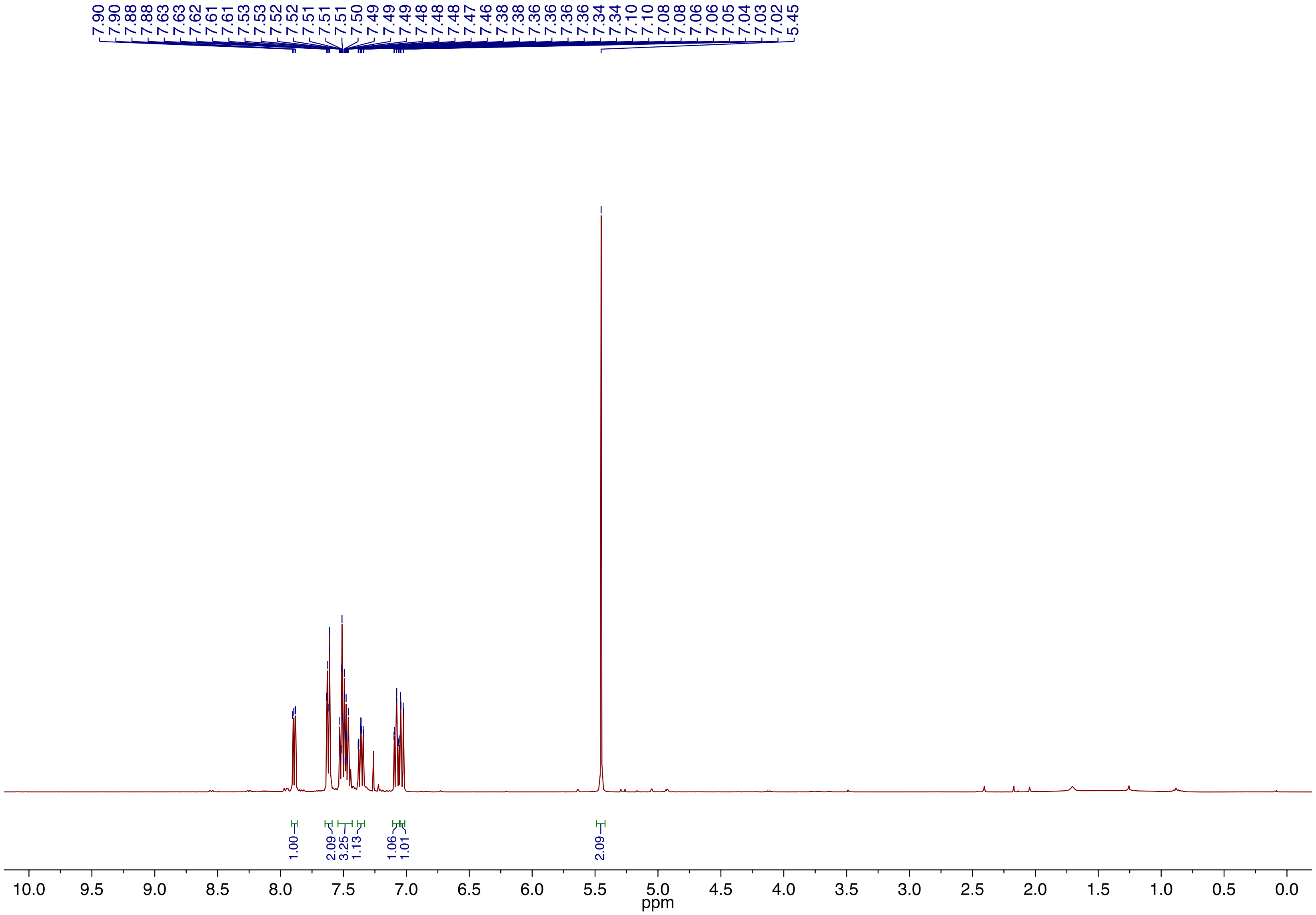

^{13}C NMR (101 MHz, $CDCl_3$)

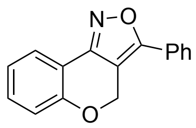

3t

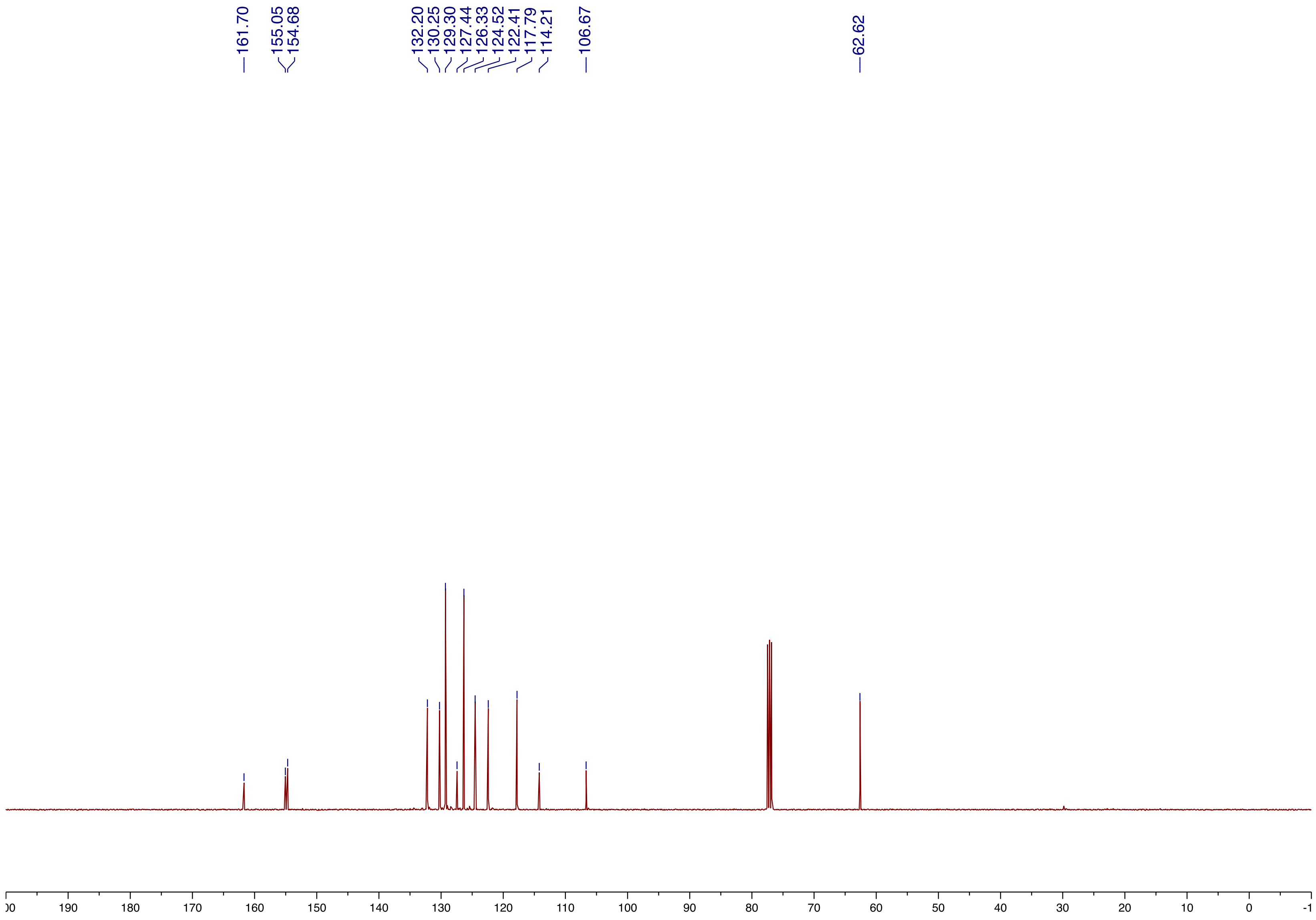

^{1}H NMR (400 MHz, $CDCl_3$)

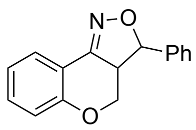

3u

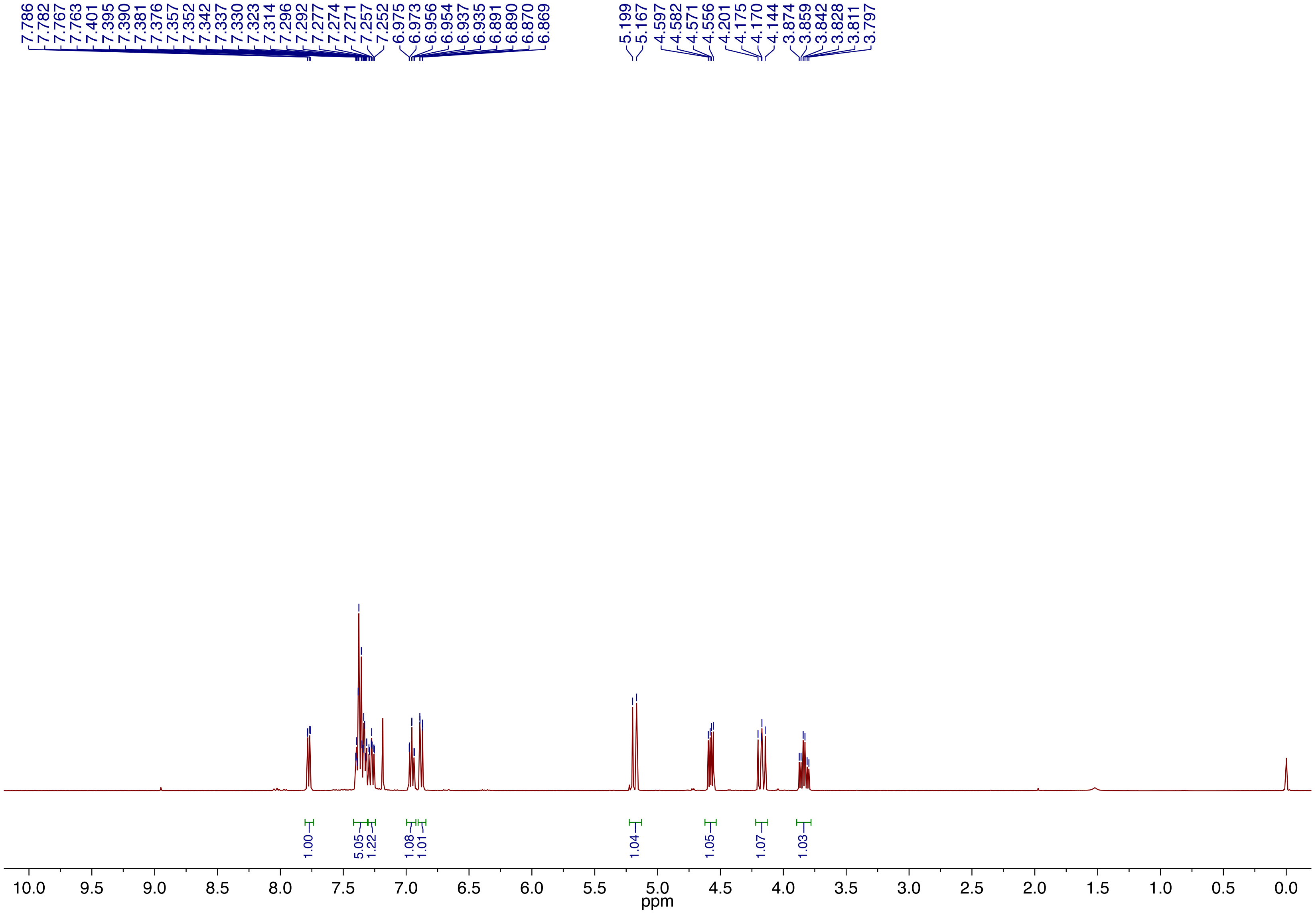

^{13}C NMR (101 MHz, $CDCl_3$)

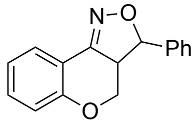

3u

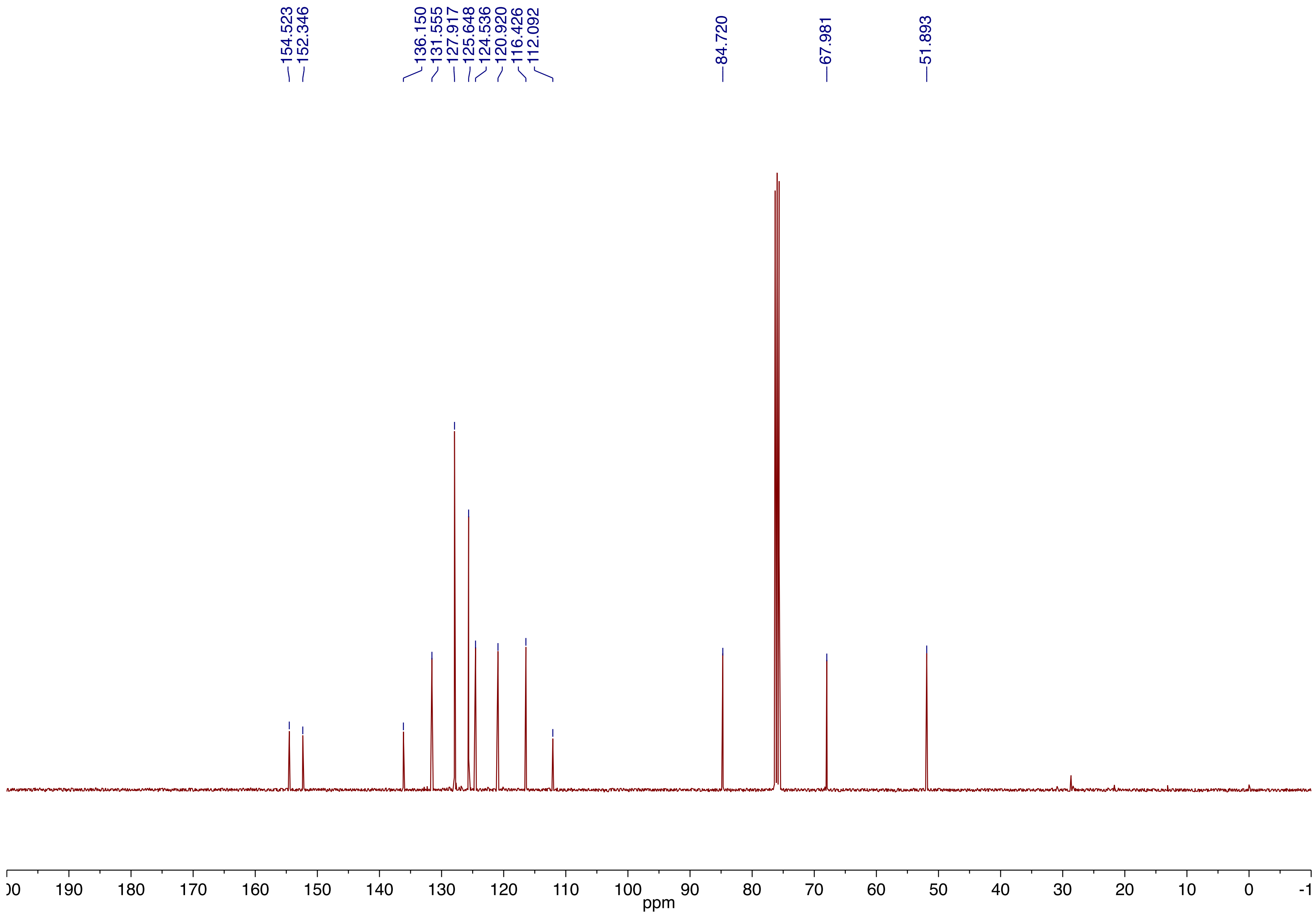

^{1}H NMR (600 MHz, $CDCl_3$)

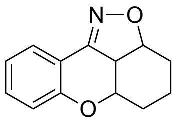

3v

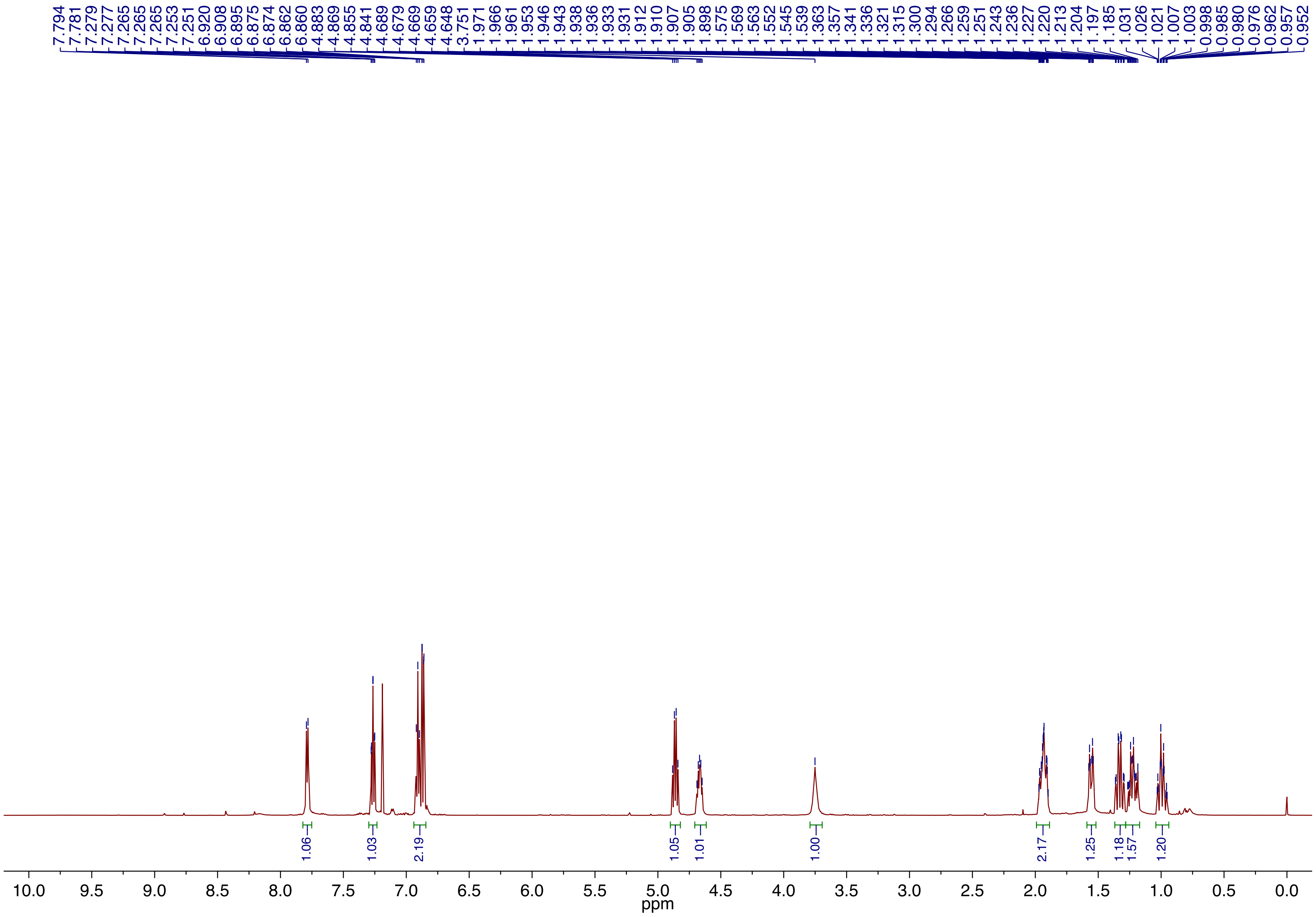

^{13}C NMR (151 MHz, $CDCl_3$)

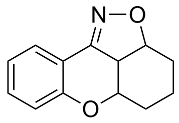

3v

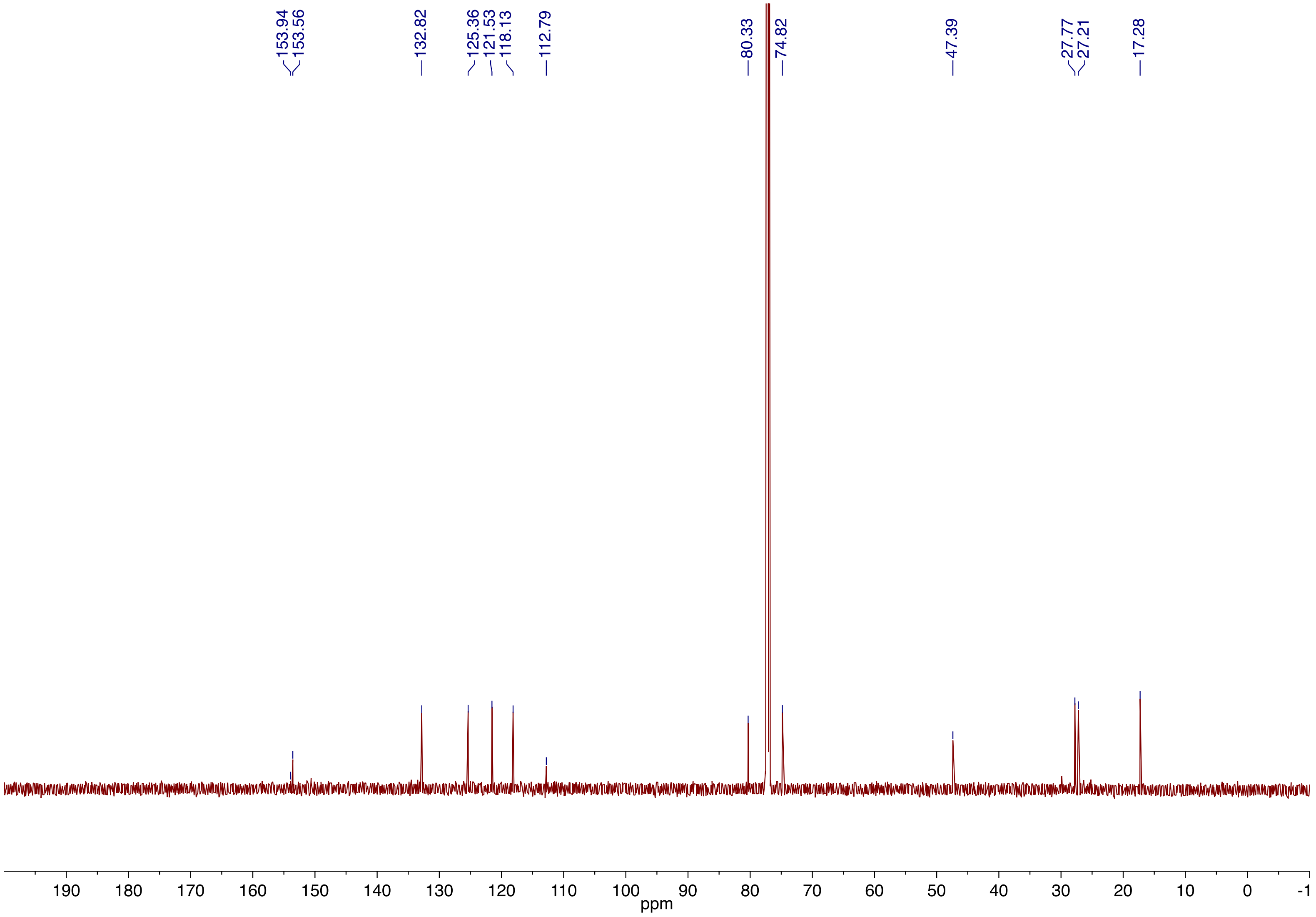

Supplement: Supplementary file 1 [file molecules-27-03860-s001.zip › molecules-1770717-supplementary.pdf]
